# Measurement Properties of Patient-Reported Outcome Measures for Diabetes: Systematic Review

**DOI:** 10.2196/25002

**Published:** 2021-08-13

**Authors:** Priscilla Jia Ling Wee, Yu Heng Kwan, Dionne Hui Fang Loh, Jie Kie Phang, Troy H Puar, Truls Østbye, Julian Thumboo, Sungwon Yoon, Lian Leng Low

**Affiliations:** 1 Duke-NUS Medical School Singapore Singapore; 2 Programme in Health Services and Systems Research Duke-NUS Medical School Singapore Singapore; 3 Department of Pharmacy National University of Singapore Singapore Singapore; 4 SingHealth Office of Regional Health Singapore Singapore; 5 Department of Rheumatology and Immunology Singapore General Hospital Singapore Singapore; 6 Department of Endocrinology Changi General Hospital Singapore Singapore; 7 Yong Loo Lin School of Medicine National University of Singapore Singapore Singapore; 8 Department of Family Medicine and Continuing Care Singapore General Hospital Singapore Singapore; 9 Post Acute and Continuing Care Outram Community Hospital SingHealth Community Hospitals Singapore Singapore

**Keywords:** systematic review, measurement properties, patient-reported outcome measures, methodological quality, level of evidence, PROMs, patient reported outcome, diabetes

## Abstract

**Background:**

The management of diabetes is complex. There is growing recognition of the use of patient-reported outcome measures (PROMs) as a standardized method of obtaining an outlook on patients’ functional status and well-being. However, no systematic reviews have summarized the studies that investigate the measurement properties of diabetes PROMs.

**Objective:**

Our aims were to conduct a systematic review of studies investigating the measurement properties of diabetes PROMs by evaluating the methodological quality and overall level of evidence of these PROMs and to categorize them based on the outcome measures assessed.

**Methods:**

This study was guided by the PRISMA (Preferred Reporting Items for Systematic Review and Meta-Analysis) guidelines. Relevant articles were retrieved from the Embase, PubMed, and PsychINFO databases. The PROMs were evaluated with the COSMIN (COnsensus-based Standards for the selection of health Measurement Instruments) guidelines.

**Results:**

A total of 363 articles evaluating the measurement properties of PROMs for diabetes in the adult population were identified, of which 238 unique PROMs from 248 studies reported in 209 articles were validated in the type 2 diabetes population. PROMs with at least a moderate level of evidence for ≥5 of 9 measurement properties include the Chinese version of the Personal Diabetes Questionnaire (C-PDQ), Diabetes Self-Management Instrument Short Form (DSMI-20), and Insulin Treatment Appraisal Scale in Hong Kong primary care patients (C-ITAS-HK), of which the C-PDQ has a “sufficient (+)” rating for >4 measurement properties. A total of 43 PROMs meet the COSMIN guidelines for recommendation for use.

**Conclusions:**

This study identified and synthesized evidence for the measurement properties of 238 unique PROMs for patients with type 2 diabetes and categorized the PROMs according to their outcome measures. These findings may assist clinicians and researchers in selecting appropriate high-quality PROMs for clinical practice and research.

**Trial Registration:**

PROSPERO International Prospective Register of Systematic Reviews CRD42020180978; https://www.crd.york.ac.uk/prospero/display_record.php?ID=CRD42020180978.

## Introduction

Diabetes is a serious and common chronic condition that affects approximately 425 million people worldwide between the ages of 20 and 79 years [1]. The management of diabetes is complex and multifaceted, as the disease is associated with various complications and imposes significant psychological and emotional burdens on the individual [2]. Successful diabetes care requires a systematic approach to support patients’ behavior change efforts, including healthy lifestyle choices, self-management, and identification of self-management problems [2]. Hence, clinical decisions made in the management of diabetes should be patient-centered, as this approach can help clinical providers identify barriers to adherence as well as motivations for self-care [2].

Recognition is growing of the usefulness of patient-reported outcome measures (PROMs) in patient-centered care and clinical decision-making [3]. PROMs are direct reports of a patient’s health status and well-being from their own perspective [4]. By providing a platform for patients to convey their disease experience, and by serving as a screening tool for underlying mental and functional problems, PROMs can bridge the gap between clinical concerns and patient perspectives, providing a more holistic assessment for enhancing diabetes care [4].

To fill this need, a considerable number of different PROMs for patients with diabetes in the adult population (>18 years of age) have been developed and revised over the last two decades. Examples include the 39-item Diabetes-39 (measuring quality of life of people with diabetes) [5] and the 20-item Problem Areas in Diabetes (PAID) Scale (measuring emotional functioning in diabetes) [6], which has since been revised to a short form 5-item scale (ie, the PAID-5 [7]). The large number of available PROMs creates challenges for clinicians or researchers to select the most appropriate high-quality PROM for their specific needs. To date, no systematic review has summarized PROMs for diabetes, whether for diabetes in general or for subpopulations of patients with diabetes (eg, patients with type 2 diabetes), nor has a review consolidated the revisions made to existing PROMs for diabetes.

Moreover, existing systematic reviews have focused on the psychometric properties of only certain categories of diabetes PROMs (eg, PROMs evaluating only health-related quality of life measures [8], PROMs for diabetes self-care [9], or PROMs in patients with diabetes associated with foot and ankle pathologies [10]) or the use of PROMs/association of PROMs with diabetes and its complications [11], even though such PROMs may be validated and applicable to a wider population of diabetes patients; for example, the Mexican version of the Diabetes Foot-Care Behavior Scale was validated in a population of patients with type 2 diabetes and not limited to patients with foot and ankle pathologies [12].

Therefore, we aimed to conduct a systematic literature review to identify studies investigating the measurement properties of PROMs validated in the population of patients with diabetes and evaluate the methodological quality and level of evidence relating to these measurement properties of PROMs. In addition, we aimed to categorize the PROMs by the type of outcome measure. This paper contains the psychometric results of the PROMs identified for patients with type 2 diabetes, and it is part of a series of papers to be published that will contain the results of the PROMs validated for (1) patients with type 1 diabetes; (2) patients with either type 1 or type 2 diabetes; and (3) patients with diabetes associated with complications such as peripheral neuropathy, retinopathy, or foot and ankle pathologies.

## Methods

### Review

This systematic review was guided by the PRISMA (Preferred Reporting Items for Systematic Review and Meta-Analyses) statement [13]. The measurement properties of each PROM were evaluated using the COSMIN (COnsensus-based Standards for the selection of health Measurement INstruments) Risk of Bias checklist [14]. The COSMIN evaluates PROM development and the following 9 measurement properties: content validity, structural validity, internal consistency, cross-cultural validity/measurement invariance, test-retest reliability, measurement error, criterion validity, hypotheses testing for construct validity, and responsiveness [14,15]. The results were used to determine the overall evidence of each PROM [16]. This systematic review has been submitted for registration on Prospero and registered on the Open Science Framework [17].

### Search Strategy

The PubMed, Embase, and PsychINFO (Ovid) databases were searched for any articles published on or before March 31, 2020. A search strategy (Tables S1-S3, Multimedia Appendix 1) of three components was used as follows [18]: disease terms (diabetes and associated terms), construct of interest (PROMs and associated variations of this term), and measurement properties (as defined under the COSMIN criteria). Where available, the sensitivity of the searches was enhanced using search filters developed by Terwee et al [19] and the PROM Group, University of Oxford [20]. The search records were downloaded into Endnote X9 (Clarivate Analytics), and any duplicates were removed.

### Article Selection

Two reviewers (PWJL and DHFL) independently screened all titles and abstracts, and a third reviewer (YHK) was consulted to make a final decision when any disagreement arose between the two reviewers as to the relevance of the articles based on the inclusion and exclusion criteria. For articles that were potentially relevant, the full-text articles were independently reviewed by the same two reviewers for inclusion and exclusion.

We included full-text original publications in English that validated PROMs for patients with diabetes mellitus and evaluated the PROMs for at least one of the nine measurement properties listed in the COSMIN guidelines. The COSMIN guidelines evaluate PROM development using the following nine measurement properties: content validity [21], structural validity, internal consistency, cross-cultural validity/measurement invariance, test-retest reliability, measurement error, criterion validity, hypotheses testing for construct validity, and responsiveness. Their definitions are presented by Mokkink et al [22].

We excluded conference abstracts and studies that focused on measurement development or that included PROMs completed by proxy or by patients ≤18 years of age. These exclusions were not used to construct the search strategy to avoid the omission of relevant studies. If only part of the study population consisted of PROMs directly reported by patients >18 years of age with diabetes, the articles were included if the results were reported separately for this group of patients. The type of study (eg, randomized controlled trial, cross-sectional study, cohort study, and registry-based study) was not part of our exclusion criteria to assess the measurement properties to ensure that this systematic literature review would be able to provide a comprehensive overview of the measurement properties of all types of PROMs.

### Data Extraction

Two reviewers (PWJL and DHFL) extracted the following data (where available) from the articles:

1. General characteristics of the study populations: sample size, age, gender, and country where the study was conducted
2. Disease characteristics of the study population: disease studied and duration of illness
3. Characteristics of the PROMs: language version used, domains assessed, number of domains and items, and response scale

### Assessment of Methodological Quality

Two reviewers (PWJL and DHFL) independently evaluated all relevant articles for methodological quality using the COSMIN Risk of Bias checklist [14], and a third reviewer (YHK) resolved any disagreement. Each measurement property was assessed based on a 4-point scale: inadequate, doubtful, adequate, or very good [14,15]. The item with the worst rating under each measurement property would determine the overall rating for the specific measurement property [23]. The assessed PROMs were then categorized according to their outcome measures.

### Assessment of Quality of Measurement Properties

The quality of measurement properties of each PROM was assessed using the quality criteria described by Terwee et al [16]. First, the measurement properties to be evaluated were identified. Next, according to the results from the study of each measurement property, a “positive (+),” “indeterminate (?),” or “negative (-)” rating was assigned [16].

### Evidence Synthesis

For each PROM, an evidence synthesis across all studies was conducted. First, we determined whether each measurement property for a PROM had overall “sufficient (+),” “insufficient (-),” “inconsistent (±),” or “indeterminate (?)” evidence. Second, we graded the quality of evidence for each measurement property of the PROM as high, moderate, low, or very low based on the guidelines from the modified Grading of Recommendations Assessment, Development and Evaluation (GRADE) approach for systematic reviews of clinical trials [15,24].

## Results

### Search Results

**Figure 1:**
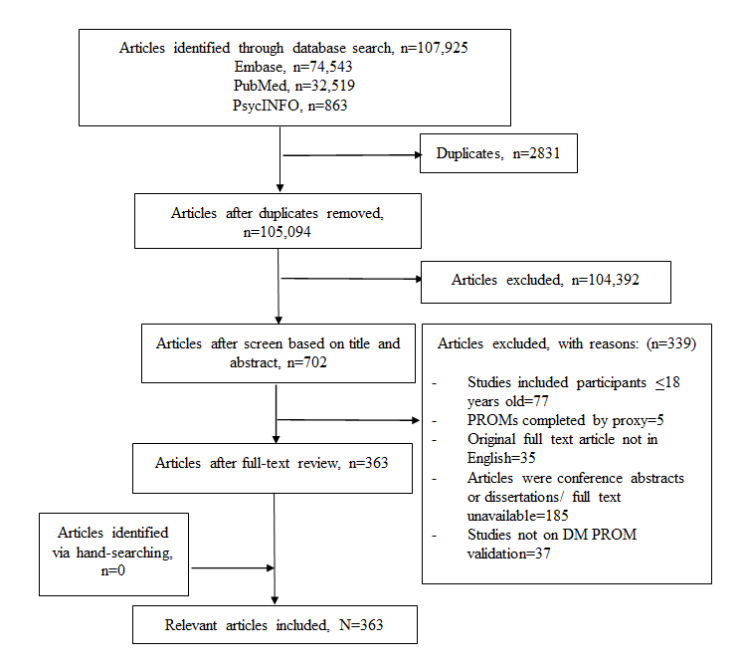
Flow chart of the systematic literature review. DM: diabetes mellitus; PROM: patient-reported outcome measure.

A total of 107,925 articles were obtained from the database search (Figure 1), of which 2831 duplicates were excluded. A review of the titles and abstracts excluded 104,392 articles. Then, after a full-text review, 339 articles were excluded for the reasons provided in Figure 1, resulting in 363 relevant articles.

Out of the 363 relevant articles, 209 articles reporting on 248 studies validated PROMs for patients with type 2 diabetes, which are the focus of the subsequent analysis in this paper. A breakdown of the 363 relevant articles identified by patient population is provided in Table 1. The results of studies assessing other diabetes subpopulations will be published elsewhere.

Out of the remaining 209 articles reporting on studies that validated PROMs for patients with type 2 diabetes, 238 unique PROMs in 35 languages from 53 countries were identified (Table 2).

**Table 1:** Breakdown of relevant articles by patient population (N=363).

Patient population	Articles, n (%)
Type 2 diabetes	209 (57.6)
Type 1 diabetes	16 (4.4)
Type 1 or Type 2 diabetes^a^	119 (32.9)
Diabetes with complications	19 (5.2)

^a^Includes articles that did not differentiate between types of diabetes. Attempts were made to contact the authors for clarification.

**Table 2:** Characteristics of the included articles.

General characteristics			Value
Unique PROMs^a^ identified, n			238
Unique countries identified, n			53^b^
Unique languages identified, n			35^c^
**Sample size,^d^ n (%)**			
	<30	6 (2.42)	
	30-49	6 (2.42)	
	50-99	24 (9.68)	
	>100	203 (81.85)	
**Mean age^d^ (years), n (%)**			
	30-39	3 (1.21)	
	40-49	10 (4.03)	
	50-59	108 (43.55)	
	60-69	80 (32.26)	
	≥70	11 (4.44)	
**Proportion of males,^e^ n (%)**			
	<0.5	114 (45.97)	
	0.5 < x <0.6	83 (33.47)	
	0.6 < x < 0.7	25 (10.08)	
	0.7 < x < 0.8	3 (1.21)	
	0.8 < x < 0.9	2 (0.81)	
**Disease characteristics: disease duration (years),^e^ n (%)**			
	0 < mean disease duration < 10	73 (29.44)	
	10 < mean disease duration < 20	67 (27.02)	
	20 < mean disease duration < 30	1 (0.40)	

^a^PROMs: patient-reported outcome measures.

^b^Some countries were not reported.

^c^Some languages were not reported.

^d^Inclusive of multiple studies reported on the same sample.

^e^Some values were reported as median and range or were not reported.

### Characteristics of the PROMs

The characteristics of the identified PROMs are presented in Table S4 in Multimedia Appendix 1. A majority of the PROMs studied were in English (27.82%), and all PROMs identified were self-administered questionnaires.

### Categories of PROMs

The 238 unique PROMs identified are categorized in Table 3. Based on the intended outcome measurements as described by the authors of the respective validation studies, the PROMs can be broadly categorized into three groups: first, general impact on quality of life questionnaires (eg, the World Health Organization Quality of Life questionnaire [WHOQOL-100] and the EuroQol 5-Dimension [EQ-5D]) (24/238,10.1%); second, questionnaires measuring diabetes-specific impacts on quality of life (eg, the 19-item Audit of Diabetes-Dependent Quality of Life [ADDQoL-19]) (42/238,17.6%), and third, questionnaires measuring specific aspects of dealing with diabetes (eg, PAID, which measures psychological impact, or the Diabetes Treatment Satisfaction Questionnaire [DTSQ], which measures satisfaction with diabetes treatment) (172/238,72.3%). The majority of the PROMs fell into the third category, and they could be further classified into as follows: general psychosocial impact (eg, social/psychological/emotional well-being of patients with diabetes), diabetes-related depression, diabetes-related distress, self-efficacy (eg, patients’ belief in their capability to organize and execute the course of action required to deal with their disease), self-management (eg, the range of activities patients must engage in on a regular basis to manage their diabetes), impact of empowerment tools (eg, the level of empowerment developed by patients as a result of educational interventions), health-promoting lifestyle behaviors (eg, in terms of physical activity or stress management), health beliefs (eg, perceived benefits of treatment), knowledge/competence, treatment experience (eg, the level of satisfaction with treatment), treatment compliance, symptoms (eg, hypoglycemia and patients’ experiences/perceptions on this), nutrition and physical activity (eg, patients’ perceptions on diet and exercise), sleep, support (eg, patients’ perspectives on the availability of support for diabetes), attitude/coping with diabetes, obstacles and problem-solving, health perception (eg, patients’ perspectives on their illness/diabetes-related health satisfaction).

**Table 3:** Patient-reported outcome measures (PROMs) organized by category.

Category	Description	PROMs^a^
General impact on quality of life	Generic PROMs that are evaluated within the population of patients with type 2 diabetes. These PROMs assess the impact of chronic illness in terms of impact on quality of life. Examples of domains assessed include physical health, psychological state, social relationships and environment, mobility, self-care, usual activities, pain/discomfort, and anxiety/depression.	WHOQOL-100^b^ [25]; WHOQOL-BREF^c^ [26]; WHOQOL-BREF (Malayalam version) [27]; WHOQOL-BREF 26 (Persian version) [28]; WHOQOL-BREF (Amharic version) [29]; RAND-12^d^ [30]; HSQ 2.0^e^ (Spanish version) [31]; HUI2^f^ [30]; HUI3^g^ [30,32]; EQ-5D^h^ [33], [34]; EQ-5D-5L^i^ [35-37]; EQ-5D-3L^j^ [38]; EQ-5D-3L (Finnish version) [38]; EQ-5D-3L (German version) [38]; EQ-5D-3L (Greek version) [38]; EQ-5D-3L (Dutch version) [38]; EQ-5D-3L (Spanish version) [38]; EQ-5D-5L (Brunei-Malay version) [39]; PACIC^k^ [40,41]; Short-version PACIC [42]; Modified-PACIC [43]; PACIC (Malay version) [44]; SF-36^l^ [45]; SF-12v2^m^ [46]
Diabetes-specific impact on quality of life	PROMs that assess the impact of diabetes on quality of life, such as impact on physical function, psychological well-being and social well-being. Examples of domains assessed include physical function, symptoms, psychological well-being, self-care management, social well-being, global judgments of health, and satisfaction with care and flexibility of treatment.	PRO-DM-Thai^n^ [47]; DQOL^o^ (Chinese version) [48,49]; DQOL (Iranian version) [50]; DQOL (Turkish version) [51]; DQOL (Malay version) [52]; IRDQOL^p^ [28]; revised version of DQOL [53]; AsianDQOL^q^ [54]; AsianDQOL (Malay version) [54]; AsianDQOL (Chinese-mandarin version) [54]; DQL-BCI^r^ (Polish version) [55]; ^s^DQOL-B [56]; QOLID^t^ [57]; J-DQOL^u^ [58]; QOL^v^ questionnaire [59]; DMQoL^w^ (Persian version) [60]; MENQOL^x^ [61]; Diabetes-39 (Arabic version) [62]; Diabetes-39 (Brazillian version) [63]; ADDQoL-19^y^ [56], [64-66]; ADDQoL-19 (Chinese version) [67]; ADDQoL-19 (Malay version) [64]; CN-ADDQoL^z^ [68]; ADDQoL (Spanish version) [69]; ADDQoL (Turkish version) [70]; Malay ADDQoL [71]; Elasy et al [72]; DHP-1^aa^ [73]; DHP-3D^ab^ [74]; DHP-5D^ac^ [74]; DCP^ad^ (Chinese version) [75]; DIMS^ae^ (Chinese version) [76]
General psychosocial impact	PROMs that assess the social/psychological/emotional well-being of patients with diabetes. Examples of domains assessed include anxiety, depressed mood, positive well-being, self-control, general health, and validity.	MDQ^af^ [77]; MDQ (Hindi version) [78]; PGWB^ag^ [33]; WBQ^ah^ [26,79]; W-BQ28^ai^ [80]; WHO-5^aj^ [81]; WHO-5 (Polish version) [82]
Diabetes-related depression	PROMs that screen for depression in patients with diabetes/monitor the presence of depressive symptoms in patients with diabetes. Examples of domains assessed include depressed affect, somatic symptoms, positive affect, and interpersonal problems.	CES-Depression^ak^ [83-86]; Depression in Diabetes Self-Rating Scale [87]; SCAD^al^ [84]; HADS^am^ [84]; DMI^an^ [84]; EDS^ao^ [88]; DCS^ap^ [89]; CUDOS-Chinese^aq^ [90]; PHQ-9^ar^ [91], [92]; PHQ-9 (Chichewa version) [93]; PHQ-9 (Romanian version) [94]
Diabetes-related distress	PROMs that screen for diabetes-related emotional distress. Examples of domains assessed include emotional burden, physician-related distress, regimen-related distress, and interpersonal distress.	CDDS-17^as^ [95]; DDS Bahasa Indonesia^at^ [96]; PAID^au^ [97]; MY-PAID-20^av^ [98]; B-PAID^aw^ [99]; PAID-K^ax^ [100]; K-PAID^ay^ [101]; K-PAID-5^az^ [101]; Turkish PAID [102]; PAID (Greek version) [103]; SG-PAID-C^ba^ [104]; PAID (Spanish version) [105]; IR-PAID-20^bb^ [106]
Self-efficacy	PROMs that assess the level of self-efficacy (ie, people’s belief in their capability to organize and execute the courses of action required to deal with prospective situations [107]) of patients with type 2 diabetes, whether in general or in dealing with specific aspects of diabetes (eg, in taking medication [108]). Examples of domains assessed include performing activities which are essential for the treatment of diabetes, self-observation, and self-regulating activities.	SE-Type 2^bc^ [107]; DMSES^bd^ [109]; K-DMSES^be^ [110]; GR-DMSES^bf^ [111]; DMSES (Brazilian version) [112]; IT-DMSES^bg^ [113]; DSEQ^bh^ (Thai version) [114]; CDMSS-11^bi^ [115]; DSCAS^bj^ [116]; DSES^bk^ [116]; K-DSES^bl^ [117]; Situational Self-Efficacy Scales (Spanish version) [31]; ESS^bm^ [118]; Self-Efficacy for Exercise 1 (Spanish version) [31]; Self-Efficacy for Exercise 2 (Spanish version) [31]; PTES^bn^ [108]
Self-management	PROMs that assess the level of diabetes self-management (ie, range of activities in which individuals must engage on a regular basis to manage their diabetes [119]). Examples of domains assessed include general diet, specific diet, exercise, medication taking, blood-glucose testing, foot care, and cigarette smoking.	SDSCA^bo^ [120]; SDSCA (Turkish version) [121]; SDSCA-G^bp^ [122]; SDSCA (Moroccan version) [123]; SDSCA-Ar^bq^ [124]; SDSCA-K^br^ [125]; INAAP-DM2^bs^ [126]; SCI-R^bt^ [127]; DSSCI^bu^ [128]; SUGAAR^bv^ [129]; D-SMART^bw^ [119]; ES-SMBPA-2D^bx^ [130]; DSMS^by^ [116]; DSMQ^bz^ (Thai version) [131]; DSMQ (Urdu version) [132]; V-DSMI^ca^ [133]; DSMI-20^cb^ [134]; DSMB-O^cc^ [135]; SMP-T2D^cd^ [136]; PAM13^ce^ [137]; Chernyak et al [138]; CIRS^cf^ (Thai version) [139]
Impact of empowerment tools	PROMs that assess the level of empowerment (ie, patients’ natural capacity and ability to become responsible for their own lives) that is discovered and developed [140] as a result of educational interventions. Examples of domains assessed include managing the psychosocial aspects of diabetes, assessing dissatisfaction, and readiness to change.	IR-DES-28^cg^ [140]; Hara et al [141]; DES-M^ch^ [142]; DES-SF^ci^ (Brazilian Portuguese version) [143]; DES-SF (Portuguese version) [144]
Health-promoting lifestyle behaviors	PROMs that assess health-promoting lifestyle behaviours of patients with diabetes. Examples of domains assessed include physical activity, risk reduction, stress management, health responsibility, enjoyment of life, and healthy diet.	T2DHPS^cj^ (Persian version) [145]; T2DHPS (Turkish version) [146]; DHPSC^ck^ (Chinese version) [147]; PDQ-11^cl^ [148]; C-PDQ^cm^ [149]
Health beliefs	PROMs that assess diabetes-specific health beliefs of patients. Examples of domains assessed include perceived benefits of and barriers to treatment and perceived severity of and vulnerability to complications.	Health Belief Measures [150]; Given Health Belief Instrument (Spanish version) [151]; Health Belief Model Scale (Turkish version) [152]; Diabetes Health Belief Measure [153]
Knowledge/ competence	PROMs that assess the level of diabetes knowledge, whether in general or for specific areas of knowledge such as nutrition knowledge. Examples of domains assessed include symptoms (eg, frequent hunger), causes and risk factors (eg, lack of physical activity), complications (eg, kidney failure), and management (eg, reduced consumption of rice).	Diabetes Questionnaire [154]; Diabetes Questionnaire (Spanish version) [154]; Diabetes Knowledge Questionnaire (Spanish version) [31]; DKQ-24^cn^ [153]; DMKT^co^ [155]; PCSD-P^cp^ [156]; Miller et al [157]; Miller and Edwards [158]; PDDC^cq^ [159]; DRNK^cr^ [160]; FCCHL^cs^ (Norwegian version) [161]; KHLS-DM^ct^ [162]; HLS-K^cu^ [163]; HLS/SNS^cv^ [164]; Ashok et al 1 [165]; Ashok et al 2 [166]; HLS-EU-Q47^cw^ [167]
Treatment experience	PROMs that assess the treatment experience in general [168] or specifically the level of satisfaction with treatment [79] or treatment burden [169] or treatment with specific modalities of treatment (eg, with pharmacotherapy [169] or insulin therapy [170]). Examples of domains assessed include efficacy, treatment burden and symptoms (side effects), diabetes worries, perceptions of insulin therapy, treatment satisfaction, and inhaler performance.	DTSQ^cx^ [79]; DTSQ (Greek version) [171]; DiabMedSat^cy^ [172]; DTBQ^cz^ [169]; ITEQ^da^ [168]; IITQ^db^ [170]; ITAS^dc^ [173]; C-ITAS-HK^dd^ [174]; BITQ^de^ (Turkish version) [175]; Ch-ASIQ^df^ [176]; MIAS^dg^ [177]; IMDSES^dh^ (Brazilian version) [178]; ITSQ^di^ [179]; OHA-Q^dj^ [180]; DMSRQ^dk^ [181]
Treatment compliance	PROMs that measure the level of compliance to treatment/adherence to medication/patients’ beliefs regarding treatment. Examples of domains assessed include emotional difficulties in compliance, physical difficulties in compliance, changing difficulties of habits in compliance, acceptance difficulties in compliance, awareness difficulties in compliance, diet difficulties in compliance, and denial difficulties in compliance.	Demirtas et al [182]; MMAS^dl^ (Thai version) [183]; modiﬁed 4-item Morisky–Green–Levine Medication Adherence Scale [184]; MMAS-8^dl^ (Korean version) [185]; MMAS-8 (Chinese version) [186]; MMAS-8 (French version) [187], [188]; MGLS^dm^ (Indonesian version) [189]; Medical Prescription Knowledge questionnaire [190]; Attitude Scale [190]; BMQ-f^dn^ [191]; MALMAS^do^ [192]; MAT OADs^dp^ [193]; MAT Insulin^dq^ [193]; ARMS-K^dr^ [194]; Diabetes Medication System Rating Questionnaire Short-Form [195]; SR-4^ds^ (French version) [187]; Zongo et al 1 [187]; Zongo et al 2 [187]
Symptoms	PROMs that assess patients’ experiences/perceptions of specific symptoms associated with diabetes (eg, hypoglycemia [196], fatigue [197]). Examples of domains assessed include symptom concern, compensatory behavior, worry, general fatigue, and physical fatigue.	HPQ^dt^ (Cyprus version) [196]; HPQ [196]; CHI^du^ (Filipino version) [198]; FH-15^dv^ (Chinese version) [199]; K-DSC-R^dw^ [200]; DSC-R^dx^ [201]; Naegeli et al [202]; FACIT^dy^-Fatigue Scale [197]
Nutrition and physical activity	PROMs that assess patients’ perspectives (eg, barriers/confidence level/knowledge) in relation to diet/nutrition and exercise/physical activity. Examples of domains assessed include satisfaction with diet, burden of diet therapy, perceived merits of diet therapy, general perception of diet, restriction of social functions, vitality, and mental health.	Barriers to Fat Reduction Scale^dz^ (Spanish version) [31]; Barriers to Exercise Checklist (Spanish version) [31]; Food Habits Questionnaire (Spanish version) [31]; DDRQOL^dz^ [203]; DDRQOL-R^ea^ [204]; Sato et al [204]; IW-SP^eb^ [205]; Motiva.Diaf-DM2 questionnaire [206]; HAPA-based PA inventory^ec^ [207]
Sleep	PROMs that assess patients’ sleep symptoms in general or specific sleep-related issues, such as obstructive sleep apnea symptoms [208].	STOP-Bang questionnaire [208]; PROMIS^ed^–Sleep Disturbance instrument [209]; PROMIS–Sleep Related Impairment instrument [209]
Support	PROMs that assess patients’ perspectives on availability of resources/support for the management of diabetes. Examples of domains assessed include individualized assessment, collaborative goal setting, enhancing skills, ongoing follow-up and support, and community resources.	RSSM-Farsi^ee^ [210]; DFBC^ef^ (Japanese version) [211]; FSS-AA T2DM^eg^ [212]; HCCQ-P^eh^ [213]; The Diabetes Family Support and Conflict Scale (Turkish version) [214]
Attitude/coping with diabetes	PROMs that assess perception toward disease, such as self-stigma (patients’ own negative attitude toward themselves [215]), relationship consciousness [216] or awareness of the psychological burden of disease [217]. Examples of domains assessed include cognitive, affective, behavioral, psychological impact of diabetes, sense of self-control, and efforts for symptom management.	SSS-J^ei^ [215]; DSAS-2^ej^ [218]; Relationship Consciousness of Japanese Patients with Type 2 Diabetes Mellitus Scale [216]; ADS^ek^ (Japanese version) [217]; ADS (Korean version) [219]; DAAS^el^ [220]; IR-DAS-3^em^ [221]; GCQ^en^ [222]; DIAB-Q^eo^ [223]; S-BRCS^ep^ [224]
Obstacles and problem-solving	PROMs that assess patients’ perspectives on obstacles to self-management/approach to manage problems in diabetes self-management/ desire to participate in medical decision-making. Examples of domains assessed include desire for discussion and desire for information, medication, self-monitoring, knowledge and beliefs, diagnosis, relationships with health care professionals, lifestyle changes, coping, and advice and support.	DPMD^eq^ [225]; DOQ^er^ [226]; DOQ (Dutch version) [227]; DOQ-30^es^ [228]; DPSS^et^ [229]
Health perception	PROMs that assess patients’ general perceptions on their illness/diabetes-related health satisfaction and knowledge of the disease or, specifically, the perception of fatalism (events are fixed such that humans are powerless to change them) [230]. Examples of domains assessed include timeline-acute/chronic, consequences, personal control, treatment control, illness coherence, emotional representation, and cause component.	IPQ-R^eu^ [231]; CHES-Q^ev^ [232]; MBIPQ^ew^ [233]; DFS^ex^ [230]

^a^PROMs: patient-reported outcome measures.

^b^WHOQOL-100: World Health Organization Quality of Life questionnaire.

^c^WHOQOL-BREF: abbreviated World Health Organization Quality of Life questionnaire.

^d^RAND-12: Veterans RAND 12-Item Health Survey.

^e^HSQ 2.0: Health Status Questionnaire 2.0.

^f^HUI2: Health Utilities Index Mark 2.

^g^HUI3: Health Utilities Index Mark 3.

^h^EQ-5D: EuroQol 5-Dimension.

^i^EQ-5D-5L: EuroQol 5-Dimension with 5-level scale.

^j^EQ-5D-3L: EuroQol 5-Dimension with 3-level scale.

^k^PACIC: Patient Assessment of Chronic Illness Care.

^l^SF-36: 36-Item Short Form Survey.

^m^SF-12v2: Short Form-12 Health Survey version 2.

^n^PRO-DM-Thai: instrument for patient-reported outcomes in Thai patients with type 2 diabetes mellitus.

^o^DQOL: Diabetes Quality-of-Life Measure.

^p^IRDQOL: Iranian Diabetes Quality of Life.

^q^AsianDQOL: Asian Diabetes Quality of Life.

^r^DQL-BCI: Diabetes Quality of Life–Brief Clinical Inventory.

^s^DQOL-B: Diabetes Quality of Life Brief Clinical Inventory.

^t^QOLID: Quality of Life Instrument for Indian Diabetes Patients.

^u^J-DQOL: Japanese version of the Diabetes Quality-Of-Life Measure.

^v^QOL: quality of life.

^w^DMQoL: Diabetes-Mellitus Specific Quality of Life.

^x^MENQOL: Menopause-specific Quality of Life.

^y^ADDQoL-19: 19-item Audit of Diabetes-Dependent Quality of Life.

^z^CN-ADDQoL: Adaptation of the ADDQoL questionnaire to people with diabetes in China.

^aa^DHP-1: Diabetes Health Profile.

^ab^DHP-3D: Diabetes Health Proﬁle–3 Dimension.

^ac^DHP-5D: Diabetes Health Proﬁle–5 Dimension.

^ad^DCP: Diabetes Care Profile.

^ae^DIMS: diabetes impact measurement scales.

^af^MDQ: Multidimensional Diabetes Questionnaire.

^ag^PGWB: Psychological General Well-Being Questionnaire.

^ah^WBQ: Well-being Questionnaire.

^ai^W-BQ28: 28-item Well-Being Questionnaire.

^aj^WHO-5: 5-item World Health Organization well-being index.

^ak^CES-Depression: Center for Epidemiological Studies Depression scale.

^al^SCAD: Silverstone Concise Assessment for Depression.

^am^HADS: Hospital Anxiety and Depression Scale.

^an^DMI: Depression in the Medically Ill Questionnaire.

^ao^EDS: Edinburgh Depression Scale.

^ap^DCS: Depressive Cognition Scale.

^aq^CUDOS-Chinese: Mandarin Chinese Version of the Clinically Useful Depression Outcome Scale.

^ar^PHQ-9: Patient Health Questionnaire-9.

^as^CDDS-17: Chinese version of the Diabetes Distress Scale.

^at^DDS Bahasa Indonesia: Indonesian Diabetes Distress Scale.

^au^PAID: Problem Areas in Diabetes scale.

^av^MY-PAID-20: Malaysian version of the Problem Areas in Diabetes scale.

^aw^B-PAID: Brazilian version of the Problem Areas in Diabetes scale.

^ax^PAID-K: Korean version of the Problem Areas in Diabetes scale.

^ay^K-PAID: Korean translation of the Problem Areas in Diabetes scale.

^az^K-PAID-5: Korean translation of the short form Problem Areas in Diabetes scale.

^ba^SG-PAID-c Chinese version of the Problem Areas in Diabetes Scale.

^bb^IR-PAID-20: Iranian version of the Problem Areas in Diabetes Scale.

^bc^SE-Type 2: self-efficacy scale for patients with type 2 diabetes mellitus.

^bd^DMSES: diabetes management self-efficacy scale.

^be^K-DMSES: Korean version of the diabetes management self-efficacy scale.

^bf^GR-DMSES: Greek version of the diabetes management self-efficacy scale.

^bg^IT-DMSES: Italian version of the diabetes management self-efficacy scale.

^bh^DSEQ: Self-Efficacy for Diabetes Scale.

^bi^CDMSS-11: Chinese version of the Diabetes Medication Self-efficacy Scale.

^bj^DSCAS: Diabetes Self-Care Agency Scale.

^bk^DSES: Diabetes Self-efficacy Scale.

^bl^K-DSES: Korean version of the Diabetes Self-efficacy Scale.

^bm^ESS: Exercise Self-efficacy Scale.

^bn^PTES: Perceived Therapeutic Efficacy Scale.

^bo^SDSCA: Summary of diabetes self-care activities measure.

^bp^SDSCA-G: German version of the Summary of diabetes self-care activities measure.

^bq^SDSCA-Ar: Arabic version of the Summary of diabetes self-care activities measure.

^br^SDSCA-K: Korean version of the Summary of diabetes self-care activities measure.

^bs^INAAP-DM2: Self-care Assessment Instrument for patients with type 2 diabetes mellitus.

^bt^SCI-R: Self-Care Inventory-Revised.

^bu^DSSCI: Diabetes Symptom Self-Care Inventory.

^bv^SUGAAR: Self-Care Utility Geriatric African-American Rating.

^bw^D-SMART: Diabetes Self-management Assessment Report Tool.

^bx^ES-SMBPA-2D: evaluation scale for self-management behavior related to physical activity of type 2 diabetic patients.

^by^DSMS: Diabetes Self-Management Scale.

^bz^DSMQ: Diabetes Self-management Questionnaire.

^ca^V-DSMI: Vietnamese version of the Diabetes Self-Management Instrument.

^cb^DSMI-20: Diabetes Self-Management Instrument Short Form.

^cc^DSMB-O: Diabetes Self-Management Behavior for Older Koreans.

^cd^SMP-T2D: self-management profile for type 2 diabetes.

^ce^PAM13: Patient Activation Measure 13.

^cf^CIRS: Chronic Illness Resources Survey.

^cg^IR-DES-28: Iranian version of the Diabetes Empowerment Scale.

^ch^DES-M: diabetes empowerment scale.

^ci^DES-SF: Diabetes Empowerment Scale–Short Form.

^cj^T2DHPS: Type 2 Diabetes and Health Promotion Scale.

^ck^DHPSC: diabetes health promotion self-care scale.

^cl^PDQ-11: Personal Diabetes Questionnaire.

^cm^C-PDQ: Chinese version of the Personal Diabetes Questionnaire.

^cn^DKQ-24: Diabetes Knowledge Questionnaire-24.

^co^DMKT: Diabetes Mellitus Knowledge Test.

^cp^PCSD-P: Persian Version of the Perceived Competence Scale for Diabetes.

^cq^PDDC: measure of perceived diabetes and dietary competence.

^cr^DRNK: diabetes-related nutrition knowledge questionnaire.

^cs^FCCHL: Functional, Communicative, and Critical Health Literacy Scale.

^ct^KHLS-DM: Korean Health Literacy Scale for Diabetes Mellitus.

^cu^HLS-K: Health Literacy Scale.

^cv^HLS/SNS: Health Literacy Scale/Subjective Numeracy Scale.

^cw^HLS-EU-Q47: European Health Literacy Survey Questionnaire.

^cx^DTSQ: Diabetes Treatment Satisfaction Questionnaire.

^cy^DiabMedSat: Diabetes Medication Satisfaction measure.

^cz^DTBQ: Diabetic Treatment Burden Questionnaire.

^da^ITEQ: insulin treatment experience questionnaire.

^db^IITQ: inhaled insulin treatment questionnaire.

^dc^ITAS: Insulin Treatment Appraisal Scale.

^dd^C-ITAS-HK: Hong Kong version of the Chinese Insulin Treatment Appraisal Scale.

^de^BITQ: Barriers to Insulin Treatment Questionnaire.

^df^Ch-ASIQ: Chinese Attitudes to Starting Insulin Questionnaire.

^dg^MIAS: Morisky Medication Adherence Scale adapted to specify insulin adherence.

^dh^IMDSES: Insulin Management Diabetes Self-Efficacy Scale.

^di^ITSQ: Insulin Treatment Satisfaction Questionnaire.

^dj^OHA-Q: Oral Hypoglycemic Agent Questionnaire.

^dk^DMSRQ: Diabetes Medication System Rating Questionnaire.

^dl^MMAS-8: 8-item Morisky Medication Adherence Scale.

^dm^MGLS: 4-item Morisky-Green-Levine Adherence Scale.

^dn^BMQ-f: French version of the Beliefs about Medicines Questionnaire.

^do^MALMAS: Malaysian Medication Adherence Scale.

^dp^MAT OADs: Measurement of Adherence to Drug Therapy in Diabetes Mellitus–Oral Antidiabetics.

^dq^MAT Insulin: Measurement of Adherence to Drug Therapy in Diabetes Mellitus–Insulin Therapy.

^dr^ARMS-K: Korean version of the Adherence to Refills and Medications Scale.

^ds^SR-4: self-report with 4 items.

^dt^HPQ: Hypoglycemia Perspectives Questionnaire.

^du^CHI: Clarke Hypoglycemia Index.

^dv^FH-15: Chinese version of the new Fear of Hypoglycemia scale.

^dw^K-DSC-R: Korean version of the Diabetes Symptom Checklist-Revised.

^dx^DSC-R: Diabetes Symptom Checklist-Revised.

^dy^FACIT: Functional Assessment of Chronic Illness Therapy.

^dz^DDRQOL: Diabetes Diet-Related Quality-of-Life scale.

^ea^DDRQOL-R: revised and short form versions of the Diabetes Diet-Related Quality of Life scale.

^eb^IW-SP: Impact of Weight on Self-Perceptions Questionnaire.

^ec^HAPA-based PA inventory: health action process approach (HAPA)–based physical activity inventory.

^ed^PROMIS: Patient-Reported Outcomes Measurement Information System.

^ee^RSSM-Farsi: Iranian version of Resources and Support for Chronic Illness Self-management scale.

^ef^DFBC: Diabetes Family Behavior Checklist.

^eg^FSS-AA T2DM: Family Support Scale Adapted for African American Women with Type 2 Diabetes Mellitus.

^eh^HCCQ-P: Persian Health Care Climate Questionnaire.

^ei^SSS-J: Japanese version of the Self-Stigma Scale.

^ej^DSAS-2: Type 2 Diabetes Stigma Assessment Scale.

^ek^ADS: Appraisal of Diabetes Scale.

^el^DAAS: Diabetes Adjustment Assessment Scale.

^em^IR-DAS-3: Iranian Diabetes Attitude Scale.

^en^GCQ: General Coping Questionnaire.

^eo^DIAB-Q: 17-item Diabetes Intention, Attitude, and Behavior Questionnaire.

^ep^S-BRCS: Spanish Brief Religious Coping Scale.

^eq^DPMD: diabetes-specific measure of patient desire to participate in medical decision making.

^er^DOQ: Diabetes Obstacles Questionnaire.

^es^DOQ-30: short version of the Diabetes Obstacles Questionnaire.

^et^DPSS: Diabetes Problem-Solving Scale.

^eu^IPQ-R: Revised Illness Perception Questionnaire.

^ev^CHES-Q: 14-item Current Health Satisfaction Questionnaire.

^ew^MBIPQ: Malay version of the Brief Illness Perception Questionnaire.

^ex^DFS: 12-item Diabetes Fatalism Scale.

### Assessment of Methodological Quality and Quality of Measurement Properties

The results from the assessment of methodological quality and quality of measurement properties of the PROMs are presented in Table S5 of Multimedia Appendix 1. In terms of validity, hypothesis testing for construct validity, structural validity, and content validity were measured for 46.8% (116/248), 49.2% (122/248), and 29.0% (72/248) of the studies, respectively. In terms of reliability, internal consistency and reliability were assessed in 79.0% (196/248) and 41.9% (104/248) of the studies, respectively.

### Evidence Synthesis

The results from the evidence synthesis of the PROMs are summarized in Table S6 in Multimedia Appendix 1. PROMs with at least a moderate level of evidence for ≥5 measurement properties include the Chinese version of the Personal Diabetes Questionnaire (C-PDQ) and the Insulin Treatment Appraisal Scale in Hong Kong primary care patients (C-ITAS-HK), of which the C-PDQ has a sufficient (+) rating for at least 4 measurement properties.

### Recommendations

According to the COSMIN guidelines [15], PROMs that have evidence for sufficient content validity and at least low-quality evidence for sufficient internal consistency can be recommended for use, and the results obtained with these PROMs can be trusted. The 43 PROMs that meet these criteria are shaded in Table S6 in grey and presented in Table S7 (Multimedia Appendix 1). They are listed below according to the categorization we have proposed in Table 3:

1. General impact on quality of life: Health Status Questionnaire 2.0 (HSQ 2.0) (Spanish version), Patient Assessment of Chronic Illness Care (PACIC)
2. Diabetes-specific impact on quality of life: instrument for patient-reported outcomes in Thai patients with type 2 diabetes mellitus (PRO-DM-Thai), Diabetes Quality-of-Life Measure (DQOL), Asian DQOL
3. Diabetes-related depression: Mandarin Chinese Version of the Clinically Useful Depression Outcome Scale (CUDOS-Chinese), Patient Health Questionnaire-9 (PHQ-9).
4. Self-efficacy: diabetes management self-efficacy scale (DMSES), Situational Self-Efficacy Scales (Spanish version), Self-Efficacy for Exercise 1 (Spanish version), Self-Efficacy for Exercise 2 (Spanish version)
5. Self-management: Diabetes Self-management Questionnaire (DSMQ), Diabetes Self-Management Instrument Short Form (DSMI-20), Chronic Illness Resources Survey (CIRS) (Thai version)
6. Impact of empowerment tools: Diabetes Empowerment Scale–Short Form (DES-SF)
7. Lifestyle behaviors: Type 2 Diabetes and Health Promotion Scale (T2DHPS), diabetes health promotion self-care scale (DHPSC) (Chinese version), C-PDQ
8. Health beliefs: Health belief model (Turkish version)
9. Knowledge/competence: Diabetes Knowledge Questionnaire (Spanish version), Diabetes Mellitus Knowledge Test (DMKT), Persian Version of Perceived Competence Scale for Diabetes (PCSD-P), Miller et al [157], the diabetes-related nutrition knowledge questionnaire (DRNK), Korean Health Literacy Scale for Diabetes Mellitus (KHLS-DM).
10. Treatment experience: C-ITAS-HK, Chinese Attitudes to Starting Insulin Questionnaire (CH-ASIQ).
11. Treatment compliance: Medical Prescription Knowledge questionnaire, Attitude Scale, Measurement of Adherence to Drug Therapy in Diabetes Mellitus–Oral Antidiabetics (MAT OADS), Measurement of Adherence to Drug Therapy in Diabetes Mellitus–Insulin Therapy (MAT insulin).
12. Symptoms: new fear of hypoglycemia scale (FH-15) (Chinese version), Functional Assessment of Chronic Illness Therapy (FACIT)–Fatigue Scale.
13. Nutrition and physical activity: Barriers to Fat Reduction Scale (Spanish version), Barriers to Exercise Checklist (Spanish version), Food Habits Questionnaire (Spanish version), health action process approach (HAPA)–based PA inventory
14. Support: Persian Health Care Climate Questionnaire (HCCQ-P), the Diabetes Family Support and Conflict Scale (Turkish version)
15. Attitude/coping with diabetes: Relationship Consciousness of Japanese Patients with Type 2 Diabetes, Diabetes Adjustment Assessment Scale (DAAS)
16. Obstacles and problem-solving: diabetes-specific measure of patient desire to participate in medical decision making (DPMD), Diabetes Obstacles Questionnaire (DOQ)

## Discussion

### Principal Findings

To the best of our knowledge, this is the first systematic review to attempt to comprehensively summarize and categorize the PROMs for type 2 diabetes and to assess their overall level of evidence based on the COSMIN guidelines. Among the 248 included studies, we identified 238 unique PROMs for patients with type 2 diabetes, with 43 unique PROMs meeting the COSMIN guidelines for recommendation for use. While our study identified a wide range of unique PROMs, based on the number of studies for each PROM (in Table S6, Multimedia Appendix 1), most of the PROMs have been validated by very few studies that evaluated them in the Type 2 diabetes population, which may bias the assessment of methodological quality and quality of measurement properties.

Nevertheless, according to the COSMIN guidelines [15], PROMs that have been validated by at least one study showing sufficient content validity and at least low quality evidence for sufficient internal consistency can be recommended for use. Our review has included recommendations for various PROM categories that should be helpful for clinicians and academics.

As illustrated in Table 3, the 43 unique PROMs identified and recommended for use measure a wide range of clinically relevant domains, ranging from impact on quality of life to specific issues such as treatment experience/treatment compliance for clinicians and researchers to apply in clinical practice. Further validation studies can also be conducted on the remaining 189 PROMs that do not meet the COSMIN guidelines for recommendation.

Measurement error was assessed in only one study, as the other studies did not report standard error of measurement, smallest detectable change, or limits of agreement as required by the COSMIN. This may be addressed in future research. In addition, although PROM translations were performed for 122 out of the 248 studies (49.2%), none of these studies assessed measurement invariance or differential item functioning; therefore, cross-cultural validity was not evaluated for any of the PROMs in this study. Further studies on measurement error and cross-cultural validity of medication adherence PROM are warranted for these studies.

### Strengths and Limitations

Our study has several strengths. We used three databases and sensitive search filters to capture as many potentially relevant articles as possible. The rigor of the study was established using the PRISMA statement and the COSMIN guidelines, which are well regarded as a consensus-based standard for evaluating the measurement properties of PROMs. The COSMIN Risk of Bias checklist employed in this study is an improvement from the original COSMIN checklist, with several improvements in the standards for evaluation [14,15]. As far as possible, we have also aimed to adopt the COSMIN guidelines for reporting the results of our evaluation. The PRISMA statement was used because it improves the transparency and clarity of the systematic review [234]. Moreover, in Table 3, we categorized all the PROMs based on their types of outcome measures. In addition to having highlighted 43 unique PROMs that are recommended for use under the COSMIN guidelines, the categorization we have proposed will provide readers with a range of options for selecting the most appropriate or robust PROM according to their required domain of assessment.

Our study has some limitations. One limitation related to this study is that the selection and evaluation of articles were subjective in nature and may have been prone to judgment bias. Further, given the scope of our study, there is inevitable potential for remaining inaccuracies in the data review/extraction process. Nevertheless, the requirement by COSMIN to have two independent reviewers and the need for a third reviewer to reach a consensus in the case of any discrepancy helps reduce the risk of judgement bias [15] and reduces the likelihood of any inaccuracies. Further, this study included only English full-text articles. Full-text articles were necessary, as they are peer-reviewed and recommended for inclusion by Terwee et al [235].

Further, in terms of the scope of our literature review, the PROMs identified may have been validated in other disease populations, or versions of the same PROMs may have been translated into other languages or culturally adapted to other populations; thus, they may have been validated in separate studies not captured by our literature review. For the validation studies of the PROMs that were identified, given the high number of studies retrieved, we were unable to hand-search all the references of the studies retrieved, and there may be additional relevant studies that were not included. Thus, such studies would be outside the scope of this review.

Finally, in comparison with previous systematic reviews on diabetes PROMs (ie, PROMs evaluating only health-related quality of life measures [8], PROMs for diabetes self-care [9], PROMs in patients with diabetes associated with foot and ankle pathologies [10], or the use of PROMs/association of PROMs with diabetes and its complications [11]), a direct comparison to the PROMs reviewed by these prior studies, which focused on diabetes complications, could not be made in this review, which focuses on reporting the results of the PROMs on type 2 diabetes (and not including complications of diabetes) identified from our review. The results of our systematic review for PROMs on the complications of diabetes will be reported separately.

### Conclusion

This review has identified 238 unique PROMs for type 2 diabetes through a systematic review and evaluated their level of evidence, adjusted using results from an assessment of methodological quality. Based on the COSMIN guidelines for evidence synthesis, PROMs with at least a moderate level of evidence for ≥5 measurement properties include the C-PDQ, DSMI-20, and the C-ITAS-HK, of which the C-PDQ has sufficient (+) ratings for at least 4 measurement properties, and based on the COSMIN guidelines, 43 unique PROMs can be recommended for use.

## Appendix

Multimedia Appendix 1 Supplementary material.

## Abbreviations

ADDQoL-19: 19-item Audit of Diabetes-Dependent Quality of Life
CH-ASIQ: Chinese Attitudes to Starting Insulin Questionnaire
CIRS: Chronic Illness Resources Survey
C-ITAS-HK: Insulin Treatment Appraisal Scale in Hong Kong primary care patients
COSMIN: COnsensus-based Standards for the selection of health Measurement Instruments
C-PDQ: Chinese version of the Personal Diabetes Questionnaire
CUDOS-Chinese: Mandarin Chinese Version of the Clinically Useful Depression Outcome Scale
DAAS: Diabetes Adjustment Assessment Scale
DES-SF: Diabetes Empowerment Scale–Short Form
DHPSC: diabetes health promotion self-care scale
DMKT: Diabetes Mellitus Knowledge Test
DMSES: diabetes management self-efficacy scale
DOQ: Diabetes Obstacles Questionnaire
DPMD: diabetes-specific measure of patient desire to participate in medical decision making
DQOL: Diabetes Quality-of-Life Measure
DRNK: diabetes-related nutrition knowledge questionnaire
DSMI-20: Diabetes Self-Management Instrument Short Form
DSMQ: Diabetes Self-management Questionnaire
DTSQ: Diabetes Treatment Satisfaction Questionnaire
ED-5Q: EuroQol 5-Dimension
FACIT: Functional Assessment of Chronic Illness Therapy
FH-15: new fear of hypoglycemia scale
GRADE: Grading of Recommendations Assessment, Development and Evaluation
HAPA: health action process approach
HCCQ-P: Persian Health Care Climate Questionnaire
HSQ 2.0: Health Status Questionnaire 2.0
KHLS-DM: Korean Health Literacy Scale for Diabetes Mellitus
MAT insulin: Measurement of Adherence to Drug Therapy in Diabetes Mellitus–Insulin Therapy
MAT OADS: Measurement of Adherence to Drug Therapy in Diabetes Mellitus–Oral Antidiabetics
PACIC: Patient Assessment of Chronic Illness Care
PAID: Problem Areas in Diabetes scale
PAID-5: short form of the Problem Areas in Diabetes scale
PCSD-P: Persian Version of Perceived Competence Scale for Diabetes
PHQ-9: Patient Health Questionnaire-9
PRISMA: Preferred Reporting Items for Systematic Review and Meta-Analysis
PRO-DM-Thai: instrument for patient-reported outcomes in Thai patients with type 2 diabetes mellitus
PROM: patient-reported outcome measure
T2DHPS: Type 2 Diabetes and Health Promotion Scale
WHOQOL-100: World Health Organization Quality of Life questionnaire
