# Supplementary material for: Measurement Properties of Patient-Reported Outcome Measures for Diabetes: Systematic Review
Source: J Med Internet Res. 2021 Aug 13;23(8):e25002. doi: 10.2196/25002 (PMC8398743; doi:10.2196/25002)
Supplement: Multimedia Appendix 1 [file jmir_v23i8e25002_app1.docx]

**Supplementary Table 1│Search Strategy for Pubmed^®^**

| **Component** | **Keywords** |
| --- | --- |
| Disease terms | 1. "Diabetes Mellitus"[Mesh] OR "diabetes mellitus”[Title/Abstract] OR “t2dm”[Title/Abstract] OR “NIDDM”[Title/Abstract] |
| Construct of interest^a^ | 1. (HR-PRO[tiab] OR HRPRO[tiab] OR HRQL[tiab] OR HRQoL[tiab] OR QL[tiab] OR QoL[tiab] OR quality of life[tw] OR life quality[tw] OR health index*[tiab] OR health indices[tiab] OR health profile*[tiab] OR health status[tw] OR ((patient[tiab] OR self[tiab] OR child[tiab] OR parent[tiab] OR carer[tiab] OR proxy[tiab]) AND ((report[tiab] OR reported[tiab] OR reporting[tiab]) OR (rated[tiab] OR rating[tiab] OR ratings[tiab]) OR based[tiab] OR (assessed[tiab] OR assessment[tiab] OR assessments[tiab]))) OR ((disability[tiab] OR function[tiab] OR functional[tiab] OR functions[tiab] OR subjective[tiab] OR utility[tiab] OR utilities[tiab] OR wellbeing[tiab] OR well being[tiab]) AND (index[tiab] OR indices[tiab] OR instrument[tiab] OR instruments[tiab] OR measure[tiab] OR measures[tiab] OR questionnaire[tiab] OR questionnaires[tiab] OR profile[tiab] OR profiles[tiab] OR scale[tiab] OR scales[tiab] OR score[tiab] OR scores[tiab] OR status[tiab] OR survey[tiab] OR surveys[tiab]))) |
| Measurement properties^b^ | 1. (instrumentation[sh] OR methods[sh] OR Validation Studies OR Comparative Study OR “psychometrics”[MeSH] OR psychometr*[tiab] OR clinimetr*[tw] OR clinometr*[tw] OR “outcome assessment, health care”[MeSH] OR outcome assessment[tiab] OR outcome measure*[tw] OR “observer variation”[MeSH] OR observer variation[tiab] OR “Health Status Indicators”[Mesh] OR “reproducibility of results”[MeSH] OR reproducib*[tiab] OR “discriminant analysis”[MeSH] OR reliab*[tiab] OR unreliab*[tiab] OR valid*[tiab] OR coefficient[tiab] OR homogeneity[tiab] OR homogeneous[tiab] OR “internal consistency”[tiab] OR (cronbach*[tiab] AND (alpha[tiab] OR alphas[tiab])) OR (item[tiab] AND (correlation*[tiab] OR selection*[tiab] OR reduction*[tiab])) OR agreement[tiab] OR precision[tiab] OR imprecision[tiab] OR “precise values”[tiab] OR test–retest[tiab] OR (test[tiab] AND retest[tiab]) OR (reliab*[tiab] AND (test[tiab] OR retest[tiab])) OR stability[tiab] OR interrater[tiab] OR inter-rater[tiab] OR intrarater[tiab] OR intra-rater[tiab] OR intertester[tiab] OR inter-tester[tiab] OR intratester[tiab] OR intra-tester[tiab] OR interobserver[tiab] OR inter-observer[tiab] OR intraobserver[tiab] OR intra-observer[tiab] OR intertechnician[tiab] OR inter-technician[tiab] OR intratechnician[tiab] OR intra-technician[tiab] OR interexaminer[tiab] OR inter-examiner[tiab] OR intraexaminer[tiab] OR intra-examiner[tiab] OR interassay[tiab] OR inter-assay[tiab] OR intraassay[tiab] OR intra-assay[tiab] OR interindividual[tiab] OR inter-individual[tiab] OR intraindividual[tiab] OR intra-individual[tiab] OR interparticipant[tiab] OR inter-participant[tiab] OR intraparticipant[tiab] OR intra-participant[tiab] OR kappa[tiab] OR kappa’s[tiab] OR kappas[tiab] OR repeatab*[tiab] OR ((replicab*[tiab] OR repeated[tiab]) AND (measure[tiab] OR measures[tiab] OR findings[tiab] OR result[tiab] OR results[tiab] OR test[tiab] OR tests[tiab])) OR generaliza*[tiab] OR generalisa*[tiab] OR concordance[tiab] OR (intraclass[tiab] AND correlation*[tiab]) OR discriminative[tiab] OR “known group”[tiab] OR factor analysis[tiab] OR factor analyses[tiab] OR dimension*[tiab] OR subscale*[tiab] OR (multitrait[tiab] AND scaling[tiab] AND (analysis[tiab] OR analyses[tiab])) OR item discriminant[tiab] OR interscale correlation*[tiab] OR error[tiab] OR errors[tiab] OR “individual variability”[tiab] OR (variability[tiab] AND (analysis[tiab] OR values[tiab])) OR (uncertainty[tiab] AND (measurement[tiab] OR measuring[tiab])) OR “standard error of measurement”[tiab] OR sensitiv*[tiab] OR responsive*[tiab] OR ((minimal[tiab] OR minimally[tiab] OR clinical[tiab] OR clinically[tiab]) AND (important[tiab] OR significant[tiab] OR detectable[tiab]) AND (change[tiab] OR difference[tiab])) OR (small*[tiab] AND (real[tiab] OR detectable[tiab]) AND (change[tiab] OR difference[tiab])) OR meaningful change[tiab] OR “ceiling effect”[tiab] OR “floor effect”[tiab] OR “Item response model”[tiab] OR IRT[tiab] OR Rasch[tiab] OR “Differential item functioning”[tiab] OR DIF[tiab] OR “computer adaptive testing”[tiab] OR “item bank”[tiab] OR “cross-cultural equivalence”[tiab]) |
|  | 1. 1 AND 2 AND 3 |

^a^Item 2 is a PROM filter developed by Mackintosh et al.

^b^Item 3 is a search filter developed by Terwee et al. to improve sensitivity of searches.

**Supplementary Table 2│Search Strategy for EMBASE^®^**

| **Component** | **Keywords** |
| --- | --- |
| Disease terms | 1. ‘diabetes mellitus’/exp OR ‘diabetes mellitus’:ab,ti OR t2dm:ab,ti OR niddm:ab,ti |
| Construct of interest^a^ | 1. 'pro':ab,ti OR 'qol':ab,ti OR 'patient reported outcome':ab,ti OR 'quality of life':ab,ti OR 'health status':ab,ti OR 'self report*':ab,ti OR 'evaluation':ab,ti OR 'status':ab,ti OR 'scale':ab,ti OR 'rating':ab,ti OR 'rated':ab,ti OR 'survey':ab,ti OR 'profile*':ab,ti OR 'questionnaire':ab,ti OR 'report*':ab,ti OR 'instrument*':ab,ti OR 'assess*':ab,ti OR 'function*':ab,ti OR 'measure*':ab,ti OR 'utilit*':ab,ti OR 'index':ab,ti OR 'indices':ab,ti OR 'wellbeing':ab,ti OR 'well being':ab,ti OR 'disability':ab,ti OR 'subjective':ab,ti |
|  |  |
| Measurement properties^b^ | 1. 'intermethod comparison'/exp OR 'data collection method'/exp OR 'validation study'/exp OR 'feasibility study'/exp OR 'pilot study'/exp OR 'psychometry'/exp OR 'reproducibility'/exp OR reproducib*:ab,ti OR "audit":ab,ti OR psychometr*:ab,ti OR clinimetr*:ab,ti OR clinometr*:ab,ti OR "observer variation"/exp OR "observer variation":ab,ti OR "discriminant analysis"/exp OR "validity"/exp OR reliab*:ab,ti OR valid*:ab,ti OR "coefficient":ab,ti OR "internal consistency":ab,ti OR (cronbach*:ab,ti AND ("alpha":ab,ti OR "alphas":ab,ti)) OR "item correlation":ab,ti OR "item correlations":ab,ti OR "item selection":ab,ti OR "item selections":ab,ti OR "item reduction":ab,ti OR "item reductions":ab,ti OR "agreement":ab,ti OR "precision":ab,ti OR "imprecision":ab,ti OR "precise values":ab,ti OR "test–retest":ab,ti OR ("test":ab,ti AND "retest":ab,ti) OR (reliab*:ab,ti AND ("test":ab,ti OR "retest":ab,ti)) OR "stability":ab,ti OR "interrater":ab,ti OR "inter-rater":ab,ti OR "intrarater":ab,ti OR "intra-rater":ab,ti OR "intertester":ab,ti OR "inter-tester":ab,ti OR "intratester":ab,ti OR "intra-tester":ab,ti OR "interobeserver":ab,ti OR "inter-observer":ab,ti OR "intraobserver":ab,ti OR "intra-observer":ab,ti OR "intertechnician":ab,ti OR "intertechnician":ab,ti OR "intratechnician":ab,ti OR "intra-technician":ab,ti OR "interexaminer":ab,ti OR "inter-examiner":ab,ti OR "intraexaminer":ab,ti OR "intraexaminer":ab,ti OR "interassay":ab,ti OR "inter-assay":ab,ti OR "intraassay":ab,ti OR "intra-assay":ab,ti OR "interindividual":ab,ti OR "inter-individual":ab,ti OR "intraindividual":ab,ti OR "intra-individual":ab,ti OR "interparticipant":ab,ti OR "inter-participant":ab,ti OR "intraparticipant":ab,ti OR "intra-participant":ab,ti OR "kappa":ab,ti OR "kappas":ab,ti OR "coefficient of variation":ab,ti OR repeatab*:ab,ti OR (replicab*:ab,ti OR "repeated":ab,ti AND ("measure":ab,ti OR "measures":ab,ti OR "findings":ab,ti OR "result":ab,ti OR "results":ab,ti OR "test":ab,ti OR "tests":ab,ti)) OR generaliza*:ab,ti OR generalisa*:ab,ti OR "concordance":ab,ti OR ("intraclass":ab,ti AND correlation*:ab,ti) OR "discriminative":ab,ti OR "known group":ab,ti OR "factor analysis":ab,ti OR "factor analyses":ab,ti OR "factor structure":ab,ti OR "factor structures":ab,ti OR "dimensionality":ab,ti OR subscale*:ab,ti OR "multitrait scaling analysis":ab,ti OR "multitrait scaling analyses":ab,ti OR "item discriminant":ab,ti OR "interscale correlation":ab,ti OR "interscale correlations":ab,ti OR ("error":ab,ti OR "errors":ab,ti AND (measure*:ab,ti OR correlat*:ab,ti OR evaluat*:ab,ti OR "accuracy":ab,ti OR "accurate":ab,ti OR "precision":ab,ti OR "mean":ab,ti)) OR "individual variability":ab,ti OR "interval variability":ab,ti OR "rate variability":ab,ti OR "variability analysis":ab,ti OR ("uncertainty":ab,ti AND ("measurement":ab,ti OR "measuring":ab,ti)) OR "standard error of measurement":ab,ti OR sensitiv*:ab,ti OR responsive*:ab,ti OR ("limit":ab,ti AND "detection":ab,ti) OR "minimal detectable concentration":ab,ti OR interpretab*:ab,ti OR (small*:ab,ti AND ("real":ab,ti OR "detectable":ab,ti) AND ("change":ab,ti OR "difference":ab,ti)) OR "meaningful change":ab,ti OR "minimal important change":ab,ti OR "minimal important difference":ab,ti OR "minimally important change":ab,ti OR "minimally important difference":ab,ti OR "minimal detectable change":ab,ti OR "minimal detectable difference":ab,ti OR "minimally detectable change":ab,ti OR "minimally detectable difference":ab,ti OR "minimal real change":ab,ti OR "minimal real difference":ab,ti OR "minimally real change":ab,ti OR "minimally real difference":ab,ti OR "ceiling effect":ab,ti OR "floor effect":ab,ti OR "item response model":ab,ti OR "irt":ab,ti OR "rasch":ab,ti OR "differential item functioning":ab,ti OR "dif":ab,ti OR "computer adaptive testing":ab,ti OR "item bank":ab,ti OR "cross-cultural equivalence":ab,ti |
|  | 1. 1 AND 2 AND 3 |

^a^Item 2 was adapted from PROM filter developed by Mackintosh et al.

^b^Item 3 is a search filter by Terwee et al. to improve sensitivity of searches.

**Supplementary Table 3│Search Strategy for PsychINFO^®^ (Ovid)**

| **Component** | **Keywords** |
| --- | --- |
| Disease terms | 1. exp diabetes mellitus/ OR ‘Diabetes Mellitus’.ti,ab. OR t2dm.ti,ab. OR NIDDM.ti,ab. |
| Construct of interest^a^ | 1. 'pro' OR 'qol' OR 'patient reported outcome' OR 'quality of life' OR 'health status' OR 'self report*' OR 'evaluation' OR 'status' OR 'scale' OR 'rating' OR 'rated' OR 'survey' OR 'profile*' OR 'questionnaire' OR 'report*' OR 'instrument*' OR 'assess*' OR 'function*' OR 'measure*' OR 'utilit*' OR 'index' OR 'indices' OR 'wellbeing' OR 'well being' OR 'disability' OR 'subjective' |
| Measurement properties^b^ | 1. 'Validation studies' OR 'Psychometr*' OR 'Measurement propert*' OR 'Validity' or 'structural validity' or 'hypothesis testing' OR 'reliability' or 'content validity' OR 'internal consistency' OR 'criterion validity' OR 'cross cultural validity' OR 'reproducibility' OR 'responsiveness' OR 'interpretability' OR 'factor analysis' OR 'coefficient' OR 'correlation' OR 'cronbach alpha' OR 'qualitative adaptation' |
|  | 1. 1 AND 2 AND 3 |

^a^Items 2 was adapted from PROM filter developed by Mackintosh et al.

^b^Items 3 was adapted from a search filter by Terwee et al.

**Supplementary Table 4│Characteristics of PROMs**

| **Category** | **PROM** | **Number of studies** | **Language versions** | **Domains assessed** | **Number of domains (items)** | **Response scale** |
| --- | --- | --- | --- | --- | --- | --- |
| General impact on quality of life | WHOQOL-100 | 1 | English | Physical health, psychological state, social relationships and environment | 4(100) | Likert scale |
|  | WHOQOL-BREF | 1 | English | Physical health, psychological health, social relationships, and environment | 4(26) | ? |
|  | WHOQOL-BREF (Malayalam version) | 1 | Malayalam | Physical health, psychological health, social relationships, and environment | 4(26) | ? |
|  | WHOQOL-BREF 26 (Persian version) | 1 | Persian | Physical health, psychological health, social relationships, and environment | 4(26) | Likert scale |
|  | WHOQOL-BREF (Amharic version) | 1 | Amharic | Physical health, psychological health, social relationships, environment, overall perception of QOL and general health | 4(26) | Likert scale |
|  | RAND-12 | 1 | English | Physical health composite and mental health composite | 2(6) | ? |
|  | HSQ 2.0 (Spanish version) | 1 | Spanish | Health perception, physical functioning, role limitations—physical health, role limitations—emotional problems, social functioning, mental health, bodily pain, and energy/fatigue | 8(39) | ? |
|  | HUI2 | 1 | English | Sensation (hearing, vision, speech), mobility, emotion, cognition, self-care, and pain | 6(15) | ? |
|  | HUI3 | 2 | English | Vision, hearing, speech, ambulation, dexterity, emotion, cognition and pain and discomfort, deﬁne health status | 8(?) | Score of-0.36-1 |
|  | EQ-5D | 1 | English | Mobility, self-care, usual activities, pain/discomfort, anxiety/depression | 5(?) | Likert scale |
|  | EQ-5D (Korean version) | 1 | Korean | ? | ? | ? |
|  | EQ-5D-5L | 3 | English | Mobility, self-care, usual activities, pain/discomfort, and anxiety/depression | 5(?) | 5 level response |
|  | EQ-5D-3L | 2 | English | Mobility, self-care, usual activities, pain/discomfort, and anxiety/depression | 5(?) | 3 level response |
|  | EQ-5D-3L (Finnish version) | 1 | Finnish | ? | ? | ? |
|  | EQ-5D-3L (German version) | 1 | German | ? | ? | ? |
|  | EQ-5D-3L (Greek version) | 1 | Greek | ? | ? | ? |
|  | EQ-5D-3L (Dutch) | 1 | Dutch | ? | ? | ? |
|  | EQ-5D-3L (Spanish) | 1 | Spanish | ? | ? | ? |
|  | EQ-5D-5L (Brunei-Malay version) | 1 | Malay | NA | 1(5) | Likert scale and VAS |
|  | PACIC | 2 | English | Patient activation, delivery system design/decision support, goal setting/tailoring, problem solving/contextual, follow-up/coordination | 5(20) | Likert scale |
|  | Short-version PACIC | 1 | English | NA | 1(11) | Likert scale |
|  | Modified-PACIC | 1 | English | Patient Activation. Delivery System Design/Decision Support, Goal Setting, Problem Solving/ Contextual Counselling, and Follow-Up/Coordination | 5(20) | Likert scale |
|  | PACIC (Malay version) | 11 | Malay | Patient activation, decision support, goal setting, problem solving and follow-up care | 5(20) | Likert scale |
|  | SF-36 |  | English | Bodily pain, general health, mental health, physical functioning, role-emotional, role-physical, social functioning, vitality | 8(36) | Likert scale |
|  | SF-12v2 |  | Chinese | Physical functioning. role physical, bodily pain, general health, vitality, social functioning, role emotional, and mental health | 8(?) | 0-100 scale |
| Diabetes-specific impact on quality of life | PRO-DM-Thai | 1 | Thai | Physical function, symptoms, psychological wellbeing, self-care management, social well-being, global judgments of health, satisfaction with care and flexibility of treatment | 7(44) | ? |
|  | DQOL (Chinese version) | 1 | Chinese | Satisfaction, impact, diabetes-related worry | 3(42) | ? |
|  | DQOL (Chinese version) | 1 | China | [Deliberately nameless] | 2(24) | Likert scale |
|  | DQOL (Iranian version) | 1 | Persian | Satisfaction with treatment, impact of treatment, worry about the future effects of diabetes, and worry about social/vocational issues | 4(46) | Likert scale |
|  | DQOL (Turkish version) | 1 | Turkish | Satisfaction, impact, diabetes worry, and social/vocational worry | 4(46) | Likert scale |
|  | DQOL (Malay version) | 1 | Malay | Diabetes life satisfaction scale, disease impact scale, disease related worries scale, and a general health questionnaire | 3(46) | Likert scale |
|  | IRDQOL | 1 | Persian | Psychological, social, physical and spiritual | 4(41) | Likert scale |
|  | revised version of DQOL | 1 | Malay | Satisfaction, impact, worry | 3(13) | Likert scale |
|  | AsianDQOL | 1 | English | Financial, diet, memory and cognition, energy, relationship | 5(21) | Likert scale |
|  | AsianDQOL (Malay version) | 1 | Malay | Financial, diet, memory and cognition, energy, relationship | 5(21) | Likert scale |
|  | AsianDQOL (Chinese-mandarin version) | 1 | Chinese-mandarin | Financial, diet, memory and cognition, energy, relationship | 5(18) | Likert scale |
|  | DQL-BCI (Polish version) | 1 | Polish | NA | 1(15) | Likert scale |
|  | DQOL-B | 1 | English | Physical function, symptoms, global judgments of health, psychological well-being, social well-being, cognitive functioning, role activities, personal constructs, satisfaction with care and flexibility of treatment | 9(15) | ? |
|  | QOLID | 1 | ? | Role limitation due to physical health (Social life, work, travelling), physical endurance, general health, treatment satisfaction, symptom botherness, financial worries, emotional/mental health, diet advise tolerance | 8(34) | Likert scale |
|  | J-DQOL | 1 | Japanese | Satisfaction with treatment, impact of treatment, worry about social/vocational issues, and diabetes worry | 5(46) | VAS |
|  | QOL questionnaire | 1 | Japanese | Degree of apprehension, degree if distress, degree of satisfaction with life and degree of satisfaction with treatments | 4(18) | Likert scale |
|  | DMQoL (Persian version) | 1 | Persian | NA | 1(10) | Likert scale |
|  | MENQOL | 1 | Malay | Vasomotor domain, psychosocial domain, physical domain, vaginal sexual domain | 4(29) | Likert scale |
|  | Diabetes-39 (Arabic version) | 1 | Arabic | Social burden, sexual functioning, anxiety/worry, diabetes control, energy/mobility | 5(39) | VAS |
|  | Diabetes-39 (Brazilian version) | 1 | Portuguese | Energy and mobility, diabetes control, anxiety and worry, social burden and sexual functioning | 5(39) | VAS |
|  | ADDQoL-19 | 2 | English | Physical function, symptoms, global judgments of health, psychological well-being, social well-being, cognitive functioning, role activities, personal constructs, satisfaction with care and flexibility of treatment | 9(19) | Likert scale |
|  | ADDQoL-19  (Chinese version) | 1 | Chinese | NA | 1(19) | Likert scale |
|  | ADDQoL-19  (Italian version) | 1 | Italian | NA | 1(19) | Score 1: +3 (excellent) to -3 (extremely bad)  Score 2: +1 (less negative impact of diabetes) to -3 (more negative impact of diabetes) |
|  | ADDQoL-19 (Malay version) | 1 | Malay | NA | 1(19) | Likert scale |
|  | ADDQoL (Chinese version) | 1 | Chinese | NA | 1(19) | Impact: -3 [greatest negative impact] to +3 [very important])  Importance: 0 (not important at all) to 2 (very important) |
|  | CN-ADDQoL | 1 | Chinese | NA | 1(19) | Impact: -3 [greatest negative impact] to +3 [very important])  Importance: 0 (not important at all) to 2 (very important) |
|  | ADDQoL (Spanish version) | 1 | Spanish | NA | 1(18) | Likert scale |
|  | ADDQoL (Turkish version) | 1 | Turkish | Employment/career, social, family, friends, sex life, sport/leisure, travel, future (own), future of family, motivation, physical activities, others fussing, enjoyment of food | 13(?) | Impact: -3 [greatest negative impact] to +3 [very important])  Importance: 0 (not important at all) to 2 (very important) |
|  | Malay ADDQOL | 1 | Malay | NA | 1(18) | Likert scale |
|  | Elasy et al. | 1 | English | Mental and social well-being | 2(18) | Likert scale |
|  | DHP-1 | 1 | English | Psychological Distress, Barriers to Activity and Disinhibited Eating | 3(32) | Likert scale |
|  | DHP-3D | 1 | English | Mood, eating and social limitations | 3(12) | ? |
|  | DHP-5D | 1 | English | Mood, eating and social limitations, hypoglycaemic attacks and vitality | 5(21) | ? |
|  | DCP (Chinese version) | 1 | Chinese | Control problems, social and personal factors, positive attitude, negative attitude, self-care ability, importance of care, self-care adherence, diet adherence, medical barriers, exercise barriers, monitoring barriers, understanding management practice, long-term care benefits, support needs, support, support attitudes | 16(234) | ? |
|  | DIMS (Chinese version) | 1 | Chinese | Symptoms, well-being, diabetes-related morale (patient attitude towards managing the disease) and social role fulfilment | 4(44) | Likert scale |
| General psychosocial impact | MDQ | 1 | French | Perceptions related to diabetes and related social support, positive and misguided reinforcing behaviors related to self-care activities, and self-efficacy and outcome expectancies. | 3(41) | Likert scale |
|  | MDQ (Hindi version) | 1 | Hindi | General perceptions of diabetes and related social support measuring interference, severity and social support | 3(41) | Likert scale |
|  | PGWB | 1 | English | Anxiety, depressed mood, positive well-being, self-control, general health and validity | 6(22) | Likert scale |
|  | WBQ | 2 | English | Depression, anxiety, energy, and positive well being | 4(22) | Likert scale |
|  | W-BQ28 | 1 | English | Generic negative well-being, generic positive wellbeing, energy, generic stress, diabetes-speciﬁc negative well-being, diabetes-speciﬁc positive well-being and diabetes-speciﬁc stress | 7(28) | Likert scale |
|  | WHO-5 well-being index | 1 | Dutch | NA | 1(5) | Likert scale |
|  | WHO-5 (Polish version) | 1 | Polish | NA | 1(5) | Likert scale |
| Diabetes-related depression | CES-Depression | 4 | English | Depressed affect, somatic symptoms, positive affect, and interpersonal problems | 1(20), 4(20), 3(14) | Likert scale |
|  | Depression in Diabetes Self-Rating Scale | 1 | Polish | NA | 1(6) | ? |
|  | SCAD | 1 | English | NA | 1(6) | ? |
|  | HADS | 1 | English | NA | 1(14) | ? |
|  | DMI | 1 | English | NA | 1(10) | Likert scale |
|  | EDS | 1 | Dutch | NA | 1(10) | Likert scale |
|  | DCS | 1 | English | NA | 1(8) | Likert scale |
|  | CUDOS-Chinese | 1 | Chinese | Affective, somatic, cognitive and behavioural | 4(18) | Likert scale |
|  | PHQ-9 | 1 | Chinese | NA | 1(9) | Likert scale |
|  | PHQ-9 | 1 | ? | Cognitive symptoms and somatic symptoms | 2(9) | Likert scale |
|  | PHQ-9 (Chichewa version) | 1 | Chichewa | NA | 1(9) | Likert scale |
|  | PHQ-9 (Romanian version) | 1 | Romanian | NA | 1(9) | Likert scale |
| Diabetes-related distress | CDDS-17 | 1 | Chinese | Emotional burden, physician-related distress, regimen related distress, and interpersonal distress | 4(17) | Likert scale |
|  | DDS Bahasa Indonesia | 1 | Bahasa Indonesian | Emotional burden, interpersonal distress, physician distress, regimen distress | 4(17) | Likert scale |
|  | PAID | 1 | English | NA | 1(20) or 1(16) | Likert scale |
|  | MY-PAID-20 | 1 | Malay | Emotional distress, treatment barriers, problems related to food and lack of social support | 4(20) | Likert scale |
|  | B-PAID | 1 | Portuguese | Emotional distress, treatment barriers, problems related to food and lack of social support | 4(20) | Likert scale |
|  | PAID-K | 1 | Korean | NA | 1(20) | Likert scale |
|  | K-PAID | 1 | Korean | 3 factor model: Diabetes-related emotional problems, food-related problems, social-support-related problems; 4 factor model: Diabetes-related emotional problems, food-related problems, social-support-related problems, treatment-related problems | 3(20) or 4(20) | Likert scale |
|  | K-PAID-5 | 1 | Korean | NA | 1(5) | Likert scale |
|  | Turkish PAID | 1 | Turkish | Diabetes distress, support-related issues | 2(20) | Likert scale |
|  | PAID (Greek version) | 1 | Greek | Emotional distress, treatment barriers, problems related to food and lack of social support | 4(20) | Likert scale |
|  | SG-PAID-C | 1 | Chinese | Emotional- and management-related problem, inability to cope with diabetes, support-related problems | 3(16) | Likert scale |
|  | PAID (Spanish version) | 1 | Spanish | NA | 1(20) | Likert scale |
|  | IR-PAID-20 | 1 | Iranian | Psychological distress in relation to diabetes management, depression- related problems and treatment barriers | 3(20) | 0-100 scale |
| Self-efficacy | SE-Type 2 | 1 | Dutch | Performing activities which are essential for the treatment of diabetes, self-observation, self-regulating activities | 3 (20) | Likert scale |
|  | DMSES | 1 | English | NA | 1(15) | Likert scale |
|  | K-DMSES | 1 | Korean | Nutrition, physical exercise/body weight, medical treatment, and blood sugar | 4(16) | Likert scale |
|  | GR-DMSES | 1 | Greek | Diet, medical therapy, medication and feet check, physical activity | 4(20) | Likert scale |
|  | DMSES (Brazilian version) | 1 | Portuguese | Specific nutrition and weight, general nutrition and medical treatment, physical exercise, and blood sugar | 4(20) | Likert scale |
|  | IT-DMSES | 1 | Italian | Disease management and lifestyles management | 2(15) | 0-10 scale |
|  | DSEQ (Thai version) | 1 | Thai | NA | 1(8) | ? |
|  | CDMSS-11 | 1 | Chinese | NA | 1(11) | ? |
|  | DSCAS | 1 | English | NA | 1(60) | ? |
|  | DSES | 1 | English | NA | 1(60) | ? |
|  | K-DSES | 1 | Korean | NA | 1(8) | 1-10 score |
|  | Situational Self-Efficacy Scales (Spanish version) | 1 | Spanish | Positive social, negative/affective, and difficulty | 3(12) | Likert scale |
|  | ESS | 1 | Dutch | NA | 1(13) | 0-100 scale |
|  | Self-Efficacy for Exercise 1 (Spanish version) | 1 | Spanish | NA | 1(5) | Likert scale |
|  | Self-Efficacy for Exercise 2 (Spanish version) | 1 | Spanish | NA | 1(8) | –100 to 100 |
|  | PTES | 1 | Persian | NA | 1(10) | Likert scale |
| Self-management | SDSCA | 1 | English | General diet, specific diet, exercise, medication taking, and blood-glucose testing, foot care, cigarette smoking | 7(11) | 8-point scale |
|  | SDSCA (Turkish version) | 1 | Turkish | Diet, exercise, blood sugar testing, foot care and medication taking | 5(21) | ? |
|  | SDSCA-G | 1 | German | Diet, exercise, blood sugar taking, footcare, smoking | 5(11) | Likert scale |
|  | SDSCA (Moroccan version) | 1 | Moroccan-arabic | Diet (general and specific), exercise, blood sugar testing, foot-care, and cigarette smoking | 5(14) | 0-7 frequency scale |
|  | SDSCA-Ar | 1 | Arabic | General diet, exercise, blood-sugar self-testing, foot care | 5(12) | 0-7 frequency scale |
|  | SDSCA-K | 1 | Korean | Diet, exercise, blood sugar testing, foot care and smoking | 5(11) | ? |
|  | INAAP-DM2 | 1 | ? | Seeking and securing appropriate multidisciplinary care, being aware of and attending to the disease and its complications, adhering to the treatment, identifying and considering/regulating the discomforts of treatment, accepting the disease and the need for healthcare, learning to live with the effects of the disease and the consequences for the lifestyle of the medical diagnosis and treatment measures | 6(131) | Likert scale |
|  | SCI-R | 1 | English | Diet, glucose monitoring, medication administration, exercise, low glucose levels, preventative/routine aspects of care | 6(15) | Likert scale |
|  | DSSCI | 1 | Spanish | NA | 1(47) | Likert scale |
|  | SUGAAR | 1 | English | NA | 1(25) | Dichotomous scale |
|  | D-SMART® | 1 | Spanish | Intermediate behavioral outcomes of exercise/physical activity, eating, medication, blood glucose monitoring, and problem solving (high and low blood sugar levels), barriers to diabetes self-management and living with diabetes (constructs of distress and support). | 7(49) | NA |
|  | ES-SMBPA-2D | 1 | Japanese | Self-management behavior to enhance physical activity in daily life, self-management behavior to maintain the level of physical activity. | 2(38) | Likert scale |
|  | DSMS | 1 | English | NA | 1(60) | ? |
|  | DSMQ (Thai version) | 1 | Thai | Glucose control, dietary control, physical activity, health-care use | 4(16) | Likert scale |
|  | DSMQ (Urdu version) | 1 | Urdu | Glucose control, dietary control, physical activity, health-care use | 4(16) | Likert scale |
|  | V-DSMI | 1 | Vietnamese | Self-regulation, self-integration, collaboration with health professionals and other signiﬁcant people, blood glucose monitoring, and adherence to recommended regimens | 5(35) | Likert scale |
|  | DSMI-20 | 1 | Chinese | Communication with health care providers, self-integration, self-monitoring of blood glucose, problem-solving | 4(35) | Likert scale |
|  | DSMB-O | 1 | Korean | Being active, healthy eating, taking medication, monitoring, problem solving, healthy coping, and reducing risks | 7(14) | Likert and dichotomous scale |
|  | SMP-T2D | 1 | English | Blood glucose monitoring, medication-taking, healthy eating, being physically active, and coping | 5(18) | 0-100 scale |
|  | PAM13 | 1 | Portuguese | ? | 1(13) | ? |
|  | *Chernyak et al.* | 1 | German | ? | ?(13) | ? |
|  | CIRS (Thai version) | 1 | Thai | Physician and health care team, family and friends, personal, neighborhood, community, media and policy and community organization | 7(63) | ? |
| Impact of empowerment tools | IR-DES-28 | 1 | Iranian | Managing the psychosocial aspects of diabetes, assessing dissatisfaction and readiness to change | 3(28) | Likert scale |
|  | Hara et al. | 1 | Japanese | Self-managed dietary behaviors, self-managed exercise behaviors, psychological impact of diabetes, patient-family communication scale | 4(30) | ? |
|  | DES-M | 1 | Malay | Managing psychosocial aspects of diabetes, assessing dissatisfaction and readiness to change, and setting and achieving diabetic goals | 3(28) | Likert scale |
|  | DES-SF (Brazilian Portuguese version) | 1 | Portuguese | Need for behavior change, development of a care plan, overcoming barriers, soliciting support, caring for oneself, managing emotions, personal motivations, and making decisions about diabetes care | 8(27) | Likert scale |
|  | DES-SF (Portuguese version) | 1 | Portuguese | NA | 1(8) | Likert scale |
| health-promoting lifestyle behaviours | T2DHPS (Persian version) | 1 | Persian | Physical activity, risk reduction, stress management, health responsibility, enjoyment of life and healthy diet | 6(28) | Likert scale |
|  | T2DHPS (Turkish version) | 1 | Turkish | Physical activity, risk reduction, stress management, enjoying life, health responsibility and a healthy diet. | 6(28) | Likert scale |
|  | DHPSC (Chinese version) | 1 | Chinese | Interpersonal relationships, personal health responsibility, diet, exercise, foot care, blood glucose self-monitoring, adherence to the recommended regimens | 7(28) | Likert scale |
|  | PDQ-11 | 1 | English | Diet-related behaviors, daily exercise routine, plans for exercising, and plans for losing weight | 4(11) | Likert scale |
|  | C-PDQ | 1 | Chinese | Diet knowledge, diet decision-making, eating problems, diet barriers, medication barriers, monitoring barriers, and exercise barriers | 7(45) | Likert scale |
| Health beliefs | Health Belief Measures | 1 | English | Perceived benefits of and barriers to treatment, perceived severity of and vulnerability to complications | 2 (24, list of diseases) | Likert scale |
|  | Given Health Belief Instrument (Spanish version) | 1 | Spanish | Total barriers, social support, impact of job on therapy, benefits of therapy | 4(25) | Score range |
|  | Health Belief Model Scale (Turkish version) | 1 | Turkish | Perceived susceptibility, perceived severity, perceived benefits, perceived barriers, recommended health-related activities | 5(36) | Likert scale |
|  | Diabetes Health Belief Measure | 1 | English/Spanish | Control, barriers, social support for diet, impact of job therapy, benefits of therapy | 4(25) | Likert scale |
| Knowledge/  competence | Diabetes Questionnaire | 1 | English | Knowledge, attitude and self-care | 3(?) | Dichotomous and likert scale |
|  | Diabetes Questionnaire (Spanish version) | 1 | English | Knowledge, attitude and self-care | 3(?) | Dichotomous and ikert scale |
|  | Diabetes Knowledge Questionnaire (Spanish version) | 1 | Spanish | NA | 1(60) | Yes/No/I don't know |
|  | DKQ-24 | 1 | English/Spanish | ? | 7(24) | Likert scale |
|  | DMKT | 1 | Indian | Symptoms (e.g., frequent hunger), causes and risk factors (e.g., lack of physical activity), complications (e.g., kidney failure), and management (e.g., reduced consumption of rice) | 4(37) | Dichotomous scale |
|  | PCSD-P | 1 | Persian | NA | 1(4) | Likert scale |
|  | Miller et al. | 1 | English | Identify dietary sources of nutrients, explain the relationship between nutrients and health, interpret claims on food labels, determine the nutrient content of products, determine appropriate amounts of products, improve decision making, discuss money-saving strategies | 7(12) | MCQ |
|  | Miller & Edwards | 1 | English | Encouraged food, discouraged food, milk/dairy; cheese, breakfast cereal, bread, pasta, rice, fresh vegetables, frozen vegetables, fruit, meat, sausage, luncheon meat, poultry, salad dressing, snack foods, frozen dinners, and frozen desserts | 16 (166) | ? |
|  | PDDC | 1 | ? | Support and communication, service logistics, technical expertise | 3(25) | ? |
|  | DRNK | 1 | English | Food portion and sizes, nutrition content of foods, healthier food choices and safety and food label reading | 4(27) | ? |
|  | FCCHL (Norwegian version) | 1 | Norwegian | Functional health literacy, communicative health literacy, and critical health literacy | 3(14) | Likert scale |
|  | KHLS-DM | 1 | Korean | ? | 3(58) | Likert scale |
|  | HLS-K | 1 | Korean | Functional, communicative, and critical health literacy | 3(14) | Likert scale |
|  | HLS/SNS | 1 | English | Functional literacy, communicative health literacy, critical health literacy, subjective numeracy | 4(22) | Likert scale |
|  | Ashok et al. 1 | 1 | ? | Knowledge, awareness | 2(15) | ? |
|  | Ashok et al. 2 | 1 | ? | Knowledge, attitude, practice | 3(15) | ? |
|  | HLS-EU-Q47 | 1 | Norwegian | Disease prevention, healthcare, health promotion, access, understand, appraise, apply | 7(47) | Likert scale |
| Treatment experience | DTSQ | 1 | English | NA | 1(8) | Likert scale |
|  | DTSQ (Greek version) | 1 | Greek | NA | 1(8) | Likert scale |
|  | DiabMedSat | 1 | English | Efficacy, treatment burden and symptoms (side effects) | 3(22) | Likert scale |
|  | DTBQ | 1 | Japanese | Implementation burden score, flexibility burden score, blood control, control burden score | 3(18) | ? |
|  | ITEQ | 1 | German | Leisure activities, psychological barriers, handling, diabetes control, dependence, weight control, sleep, and one general treatment satisfaction | 8(28) | ? |
|  | IITQ | 1 | English | Diabetes worries, perceptions of insulin therapy, treatment satisfaction and inhaler performance | 4(25) | Likert scale |
|  | ITAS | 1 | English | Positively and negatively worded items | 2(20) | Likert scale |
|  | C-ITAS-HK | 1 | Chinese | Positive and negative statements | 2(20) | Likert scale |
|  | BITQ (Turkish version) | 1 | Turkish | Fear of injections and self-testing, expectations regarding positive insulin-related outcomes, expected hardship from insulin treatment, stigmatization by insulin injections, and fear of hypoglycemia | 4(14) | Likert scale |
|  | Ch-ASIQ | 1 | Chinese | Self-image and stigmatization, factors promoting self-efficacy, fear of pain or needles, and time and family support | 4(13) | Likert scale |
|  | MIAS | 1 | English | NA | 1(8) | Likert and dichotomous scale |
|  | IMDSES (Brazilian version) | 1 | Portuguese | General management, diet, exercise, feet care, blood glucose monitoring, insulin administration and prevention, detection and treatment of hypoglycemia/hyperglycemia. | 7(28) | Likert scale |
|  | ITSQ | 1 | English | Regimen inconvenience, lifestyle flexibility, glycemic control, hypoglycemic control, and satisfaction with the insulin delivery device | 5(22) | Ordinal scale |
|  | OHA-Q | 1 | Japanese | Current status of treatment adherence, operability of treatment, social living, daily living, appreciation, somatic symptoms, satisfaction | 7(20) | Likert scale |
|  | DMSRQ | 1 | English | Convenience, negative events, interference, self-monitoring of blood glucose burden, efﬁcacy, social burden, psychological well-being, treatment satisfaction, treatment preference | 9(52) | 0-100 scale |
| Treatment compliance | Demirtas et al. | 1 | Turkish | Emotional difficulties in compliance, physical difficulties in compliance, changing difficulties of habits in compliance, acceptance difficulties in compliance, awareness difficulties in compliance, diet difficulties in compliance, denial difficulties in compliance | 7(33) | Likert scale |
|  | MMAS (Thai version) | 1 | Thai | Forgetting to take medications, stopping medications when feeling better or worse, and the complexity of the drug regime | 3(8) | Likert and dichotomous scale |
|  | modiﬁed 4-item Morisky–Green–Levine Medication Adherence Scale | 1 | English or Chinese | NA | 1(4) | Likert scale |
|  | MMAS-8 (Korean version) | 1 | Korean | NA | 1(8) | ? |
|  | MMAS-8 (Chinese version) | 1 | Chinese | NA | 1(8) | Likert and dichotomous scale |
|  | MMAS-8 (French version) | 1 | French Canadian | NA | 1(8) | Likert scale |
|  | MMAS-8 (French version) | 1 | French | Non-intentional, intentional | 2(8) | Likert scale |
|  | MGLS (Indonesian version) | 1 | Bahasa Indonesia | NA | 1(4) | ? |
|  | Medical Prescription Knowledge questionnaire | 1 | English | NA | 1(3) | Likert scale |
|  | Attitude Scale | 1 | English | Well-being/discomfort, belief about the damage caused by medication, the diabetes-treatment complications relationship, barriers/facilitators to take medication, accessibility to healthcare and medical treatment, accessibility to healthcare and medical treatment, doctor-patient agreements about treatment | 6(11) | Likert scale |
|  | BMQ-f | 1 | French | Specific and general beliefs | 2(18) | Likert scale |
|  | MALMAS | 1 | English | NA | 1(8) | Likert scale |
|  | MAT OADs | 1 | English | NA | 1(7) | Likert scale |
|  | MAT insulin | 1 | English | NA | 1(7) | Likert scale |
|  | ARMS-K | 1 | Korean | Adherence with taking medications and adherence with the refilling of prescriptions | 2(12) | Likert scale |
|  | Diabetes Medication System Rating Questionnaire Short-Form | 1 | English | Speciﬁc aspects of the medication system, factors likely to be affected by the participant’s medication system, global judgments of the medication system, and treatment preference if the respondent previously used another diabetes medication regimen | 9(19) | 0-100 |
|  | SR-4 (French version) | 1 | French Canadian | NA | 1(4) | Dichotomous scale |
|  | Zongo et al. 1 | 1 | French Canadian | NA | 1(2) | Proportion of missed pills |
|  | Zongo et al. 2 | 1 | French Canadian | NA | 1(1) | Likert scale |
| Symptoms | HPQ (Cyprus version) | 1 | Greek | Symptom concern, compensatory behavior, and worry | 3(16) | NRS |
|  | HPQ | 1 | English | Symptom concern, compensatory behavior, and worry | 3(16) | NRS |
|  | CHI (Filipino version) | 1 | Filipino | NA | 1(7) | Likert scale and VAS |
|  | FH-15 (Chinese version) | 1 | Chinese | Fear, interference, avoidance | 3(15) | Likert scale |
|  | K-DSC-R | 1 | Korean | Psychological fatigue, neuropathic pain, sensory neuropathic, cardiovascular, ophthalmologic, hyperglycemic, and hypoglycemic subscales | 7(29) | ? |
|  | DSC-R | 1 | English | Hyperglycemic, hypoglycemic, psychological cognitive, psychological-fatigue, cardiovascular, neurological pain, neurological-sensory, and ophthalmologic | 8(34) | Likert scale |
|  | Naegeli et al. | 1 | English | Cognitive distress, fatigue, hyperglycemia, and hypoglycemia | 4(15) | Likert scale |
|  | FACIT-Fatigue Scale | 1 | Turkish | General fatigue and physical fatigue | 2(13) | Likert scale |
| Nutrition and physical activity | Barriers to Fat Reduction Scale (Spanish version) | 1 | Spanish | NA | 1(9) | Likert scale |
|  | Barriers to Exercise Checklist (Spanish version) | 1 | Spanish | NA | 1(7) | Likert scale |
|  | Food Habits Questionnaire (Spanish version) | 1 | Spanish | NA | 1(18) | 4 level scale |
|  | DDRQOL | 1 | ? | Satisfaction with diet, burden of diet therapy, perceived merits of diet therapy, general perception of diet, restriction of social functions, vitality and mental health | 7(29) | ? |
|  | DDRQOL-R | 1 | Japanese | Satisfaction with diet, burden of diet therapy, perceived merits of diet therapy, general perception of diet, restriction of social functions, vitality, mental health | 7(17) | ? |
|  | Sato et al. | 1 | Japanese | Satisfaction with diet, burden of diet therapy, perceived merits of diet therapy | 3(9) | ? |
|  | IW-SP | 1 | English | NA | 1(3) | Likert scale |
|  | Motiva.Diaf-DM2 questionnaire | 1 | Spanish | 14 items assessed adherence to dietary recommendations and 6 items assessed adherence to physical activity recommendations | 2(20) | Likert scale |
|  | HAPA-based PA inventory | 1 | Persian | Risk perception, action self-efficacy, outcome expectations, behavioural intentions, action and coping planning, maintenance self-efficacy, recovery self-efficacy | 7(36) | Likert scale |
| Sleep | STOP-Bang questionnaire | 1 | Chinese | ? | ? | Dichotomous scale |
|  | PROMIS - Sleep Disturbance instrument | 1 | English | ? | ?(8) | ? |
|  | PROMIS - Sleep Related Impairment instrument | 1 | English | ? | ?(8) | ? |
| Support | RSSM-Farsi | 1 | Farsi | Individualized assessment, collaborative goal setting, enhancing skills, ongoing follow-up and support, and community resources | 5(17) | Likert scale |
|  | DFBC (Japanese version) | 1 | Japanese | Positive and negative feedback | 2(14) | Likert scale |
|  | FSS-AA T2DM | 1 | English | Parent and spouse/partner support, community and medical support, and extended family and friends support | 3(16) | Likert scale |
|  | HCCQ-P | 1 | Persian | NA | 1(15) | Likert scale |
|  | The Diabetes Family Support and Conflict Scale (Turkish version) | 1 | Turkish | Family support, family conflict | 2(10) | Likert scale |
| Attitude/coping with diabetes | SSS-J | 1 | Japanese | Cognitive, affective, behavioural | 3(39) | Likert scale |
|  | DSAS-2 | 1 | English | Treated differently, blame and judgment, self-stigma | 3(19) | Likert scale |
|  | Relationship Consciousness of Japanese Patients with Type 2 Diabetes Mellitus Scale | 1 | Japanese | Perceived beneﬁts of the relationship beneﬁt, perceived barriers to the relationship, perceived severity of diabetes, positive attitude toward the relationship with others, negative attitude toward relationships with others, perceived self-management ability | 6(36) | Likert scale |
|  | ADS (Japanese version) | 1 | Japanese | Psychological impact of diabetes, sense of self-control, efforts for symptom management | 3(7) | Likert scale |
|  | ADS (Korean version) | 1 | Korean | Psychological impact of diabetes, sense of self-control | 2(6) | Likert scale |
|  | DAAS | 1 | ? | Reshape, seek to acceptance of illness, normal life with the disease, initial self-management, comparing, initial imaging of illness, return to resources, and advanced self- management | 8(45) | Likert scale |
|  | IR-DAS-3 | 1 | Persian | Managing the psychosocial aspects of diabetes, assessing dissatisfaction and readiness to change, and setting and achieving diabetes goals | 3(33) | Likert scale |
|  | GCQ | 1 | Swedish | Self-trust, problem-reducing actions, change of values, social trust, minimization, fatalism, resignation, protest, isolation and intrusion | 10(48) | Likert scale |
|  | DIAB-Q | 1 | English | Subjective norm, attitude, PBC, intention, planning, and behavior | 6(17) | Likert scale |
|  | S-BRCS | 1 | Spanish | Positive religious coping and negative religious coping | 2(14) | Likert scale |
| Obstacles and problem-solving | DPMD | 1 | English | Desire for discussion and desire for information | 2 (11) | Likert scale |
|  | DOQ | 1 | English | Medication, self monitoring, knowledge and beliefs, diagnosis, relationships with health-care professionals, lifestyle changes, coping, and advice and support | 8(77) | Likert scale |
|  | DOQ (Dutch version) | 1 | Dutch | Medication, self monitoring, knowledge and beliefs, diagnosis, relationships with health-care professionals, lifestyle changes, coping, and advice and support | 8(113) | Likert scale |
|  | DOQ-30 | 1 | Native languages of Belgium, France, Estonia, Serbia, Slovenia, and Turkey | Relationships with medical professionals, Support from friends and family, Knowledge of the disease, Lifestyle changes, Exercising, Self-monitoring, Uncertainty about a consultation, Medication, Insulin-use | 9(30) | Likert scale |
|  | DPSS | 1 | English | Effective problem solving, impulsive problem solving, avoidant problem solving, positive transfer/learning, negative transfer/learning, positive motivational factors, negative motivational factors | 7(30 | Likert scale |
| Health perception | IPQ-R | 1 | English | Timeline-acute/chronic, consequences, personal control, treatment control, illness coherence, emotional representation, cause component | 7(?) | Likert scale |
|  | CHES-Q | 1 | English | NA | 1(14) | Likert scale |
|  | MBIPQ | 1 | Malay | NA | 1(9) | 0-10 scale |
|  | DFS | 1 | English | Emotional distress, religious and spirituality coping | 3(12) | Likert scale |

?: information was not reported.

**Abbreviations:**

VAS: visual analogue scale

NRS: numeric rating scale

WHOQOL-100: World Health Organisation quality of life questionnaire; WHOQOL-BREF/ WHOQOL-BREF 26: abbreviated World Health Organization Quality of Life; WHOQOL-BREF 26; HSQ 2.0: Health Status Questionnaire 2.0; HUI2: Health Utilities Index Mark 2; HUI3: Health Utilities Index Mark 3; EQ-5D: EuroQol 5-Dimension; EQ-5D-5L: EQ-5D with 5 level scale; EQ-5D-3L: EQ-5D with 3 level scale; PACIC: Patient Assessment of Chronic Illness Care; SF-36: 36-Item Short Form Survey; SF-12v2: Short Form-12 Health Survey version 2; MDQ: Multidimensional Diabetes Questionnaire; PDQ-11: Personal Diabetes Questionnaire; C-PDQ: Chinese version of PDQ; PRO-DM-Thai: instrument for patient-reported outcomes in Thai patients with type 2 diabetes mellitus; DQOL: Diabetes Quality-of-Life Measure; IRDQOL: Iranian diabetes quality of life; AsianDQOL: Asian Diabetes Quality of Life; DQL-BCI: Diabetes Quality of Life - Brief Clinical Inventory; DQOL-B: Diabetes Quality of Life Brief Clinical Inventory; QOLID: Quality of Life Instrument for Indian Diabetes patients; J-DQOL: Japanese version of the Diabetes Quality-Of-Life; QOL: quality of life; DMQoL: Diabetes-Mellitus Specific Quality of Life; MENQOL: Menopause-specific Quality of Life; ADDQoL-19: 19-item Audit of Diabetes-Dependent Quality of Life; CN-ADDQoL: Adaptation of the ADDQoL questionnaire to people with diabetes in China; DHP-1: Diabetes Health Profile; DHP-3D: Diabetes Health Proﬁle–3 Dimension; DHP-5D: Diabetes Health Proﬁle–5 Dimension; DCP: Diabetes Care Profile; DIMS: diabetes impact measurement scales; PGWB: Psychological General Well-Being Questionnaire; WBQ: Well-being Questionnaire; W-BQ28: 28-item Well-Being Questionnaire; CES-Depression: Center for Epidemiological Studies Depression scale; SCAD: Silverstone Concise Assessment for Depression; HADS: Hospital Anxiety and Depression Scale; DMI: Depression in the Medically Ill Questionnaire; EDS: Edinburgh Depression Scale; DCS: Depressive Cognition Scale; CUDOS-Chinese: Mandarin Chinese Version of the Clinically Useful Depression Outcome Scale; CDDS-17: Chinese version of the Diabetes Distress Scale; DDS Bahasa Indonesia: Indonesian Diabetes Distress Scale; WHO-5 well-being index: 5-item World Health Organization well-being index; PAID: Problem Areas in Diabetes; MY-PAID-20: Malaysian version of PAID; B-PAID: Brazilian version of PAID; PAID-K: Korean version of PAID; short form Korean version of PAID; IR-PAID-20: Iranian version of PAID; PHQ-9: Patient Health Questionnaire-9; SE-Type 2: self-efficacy scale for patients with type 2 diabetes mellitus; DMSES: diabetes management self-efficacy scale; K-DMSES: Korean version of DMSES; GR-DMSES Greek version of DMSES; IT-DMSES: Italian version of DMSES; CDMSS-11: Chinese version of Diabetes Medication Self-efficacy Scale; DSCAS: Diabetes Self-Care Agency Scale; SDSCA: Summary of diabetes self-care activities measure; SDSCA-G: German version of SDSCA; SDSCA-Ar: Arabic version of SDSCA; SDSCA-K: Korean version of SDSCA; INAAP-DM2: Self-care Assessment Instrument for patients with type 2 diabetes mellitus; SCI-R: Self-Care Inventory-Revised; DSSCI: Diabetes Symptom Self-Care Inventory; SUGAAR: Self-Care Utility Geriatric African-American Rating; DSES: Diabetes Self-efficacy Scale; K-DSES: Korean version of DSES; ESS: Exercise Self-efficacy Scale; D-SMART®: Diabetes Self-management Assessment Report Tool; ES-SMBPA-2D: evaluation scale for self-management behavior related to physical activity of type 2 diabetic patients; DSMS: Diabetes Self-Management Scale; DSMQ: Diabetes Self-management Questionnaire; V-DSMI: Vietnamese version of diabetes self-management instrument; DSMI-20: Diabetes Self-Management Instrument Short Form; DSMB-O: Diabetes Self-Management Behavior for Older Koreans; SMP-T2D: self-management profile for type 2 diabetes; IR-DES-28: Iranian version of Diabetes Empowerment Scale; DES-M: diabetes empowerment scale; DES-SF: Diabetes Empowerment Scale - Short Form; T2DHPS: Type 2 Diabetes and Health Promotion Scale; DHPSC: diabetes health promotion self-care scale; PAM13: Patient Activation Measure; DKQ-24: Diabetes Knowledge Questionnaire; DMKT: Diabetes Mellitus Knowledge Test; PCSD-P: Persian Version of Perceived Competence Scale for Diabetes; PDDC: measure of perceived diabetes and dietary competence; DRNK: diabetes-related nutrition knowledge questionnaire; FCCHL: Functional, Communicative, and Critical Health Literacy Scale; KHLS-DM: Korean Health Literacy Scale for Diabetes Mellitus; HLS-K: Health Literacy Scale; HLS/SNS: Health Literacy Scale/Subjective Numeracy Scale; HLS-EU-Q47: European Health Literacy Survey Questionnaire; DTSQ: Diabetes Treatment Satisfaction Questionnaire; DiabMedSat: Diabetes Medication Satisfaction measure; PTES: Perceived Therapeutic Efficacy Scale; DTBQ: Diabetic Treatment Burden Questionnaire; ITEQ: insulin treatment experience questionnaire; IITQ: inhaled insulin treatment questionnaire; ITAS: insulin treatment appraisal scale; C-ITAS-HKeq; BITQ: barriers to insulin treatment questionnaire; Ch-ASIQ: Chinese Attitudes to Starting Insulin Questionnaire; MIAS: Morisky Medication Adherence Scale adapted to specify insulin adherence; IMDSES: Insulin Management Diabetes Self-Efficacy Scale; ITSQ: Insulin Treatment Satisfaction Questionnaire; MMAS/MMAS-8: 8-item Morisky Medication Adherence Scale; MGLS: 4 items Morisky Green Levine Adherence Scale; OHA-Q: Oral Hypoglycemic Agent Questionnaire; DMSRQ: Diabetes Medication System Rating Questionnaire; BMQ-f: French version of the Beliefs about Medicines Questionnaire; MALMAS: Malaysian Medication Adherence Scale; MAT OADs: Measurement of Adherence to Drug Therapy in Diabetes Mellitus – Oral Antidiabetics; MAT insulin: Measurement of Adherence to Drug Therapy in Diabetes Mellitus – Insulin Therapy; ARMS-K: Korean version of the Adherence to Refills and Medications Scale; SR-4: self-report with 4 items; HPQ: Hypoglycemia Perspectives Questionnaire; CHI: Clarke Hypoglycemia Index; FH-15: Chinese version of the new fear of hypoglycemia scale; K-DSC-R: Korean version of Diabetes Symptom Checklist-Revised; DSC-R: Diabetes Symptom Checklist-Revised; DDRQOL: diabetes diet-related quality-of-life scale; DDRQOL-R: revised and short form versions of diabetes diet-related quality of life scale; IW-SP: Impact of Weight on Self-Perceptions Questionnaire; HAPA-based PA inventory: health action process approach (HAPA)-based PA inventory; RSSM-Farsi: Iranian version of Resources and Support for Chronic illness Self-management scale; DFBC: Diabetes Family Behavior Checklist; FSS-AA T2DM: Family Support Scale Adapted for African American Women with Type 2 Diabetes Mellitus; HCCQ-P: Persian health care climate questionnaire; SSS-J: Japanese version of the self-stigma scale; DSAS-2: Type 2 Diabetes Stigma Assessment Scale; ADS: Appraisal of Diabetes Scale; DAAS: Diabetes Adjustment Assessment Scale; IR-DAS-3: Iranian Diabetes Attitude Scale; GCQ: General Coping Questionnaire; DIAB-Q: 17-item Diabetes Intention, Attitude, and Behavior Questionnaire; S-BRCS: Spanish Brief Religious Coping Scale; DPMD: diabetes-specific measure of patient desire to participate in medical decision making; DOQ: Diabetes Obstacles Questionnaire; DOQ-30: short version of the diabetes obstacles questionnaire; DPSS: Diabetes Problem-Solving Scale; CIRS: Chronic Illness Resources Survey; IPQ-R: Revised Illness Perception Questionnaire; CHES-Q: 14-item Current Health Satisfaction Questionnaire; MBIPQ: Malay version of the brief illness perception questionnaire; DFS: 12-item diabetes fatalism scale

**Supplementary Table 5**│**Assessment of methodological quality of studies and quality of measurement properties of PROMs**

|  | |  |  | **PROM development** | **Methodological quality of studies/ quality of measurement properties of PROM** | | | | | | | | |
| --- | --- | --- | --- | --- | --- | --- | --- | --- | --- | --- | --- | --- | --- |
| **S/No.** | **PROM** | | **Reference** |  | **Contentvalidity** | **Structural validity** | **Internal consistency** | **Cross-cultural validity** | **Reliability** | **Measurement error** | **Criterion validity** | **Hypothesis testing** | **Responsiveness** |
| 1 | | WHOQOL-100 | Pibernik-Okanović, M.,2001 [26] | 0 | 0 | 0 | Very good/+ | 0 | Doubtful/? | 0 | 0 | Very good/+ | Very good/+ |
| 2 | | WHOQOL-BREF | -* | Inadequate | 0 | Adequate/? | Very good/- | 0 | Inadequate/? | 0 | 0 | Adequate/? | 0 |
|  | | WHOQOL-BREF | Kolawole, B. A. et al.,2009 [27] | 0 | 0 | 0 | Very good/- | 0 | 0 | 0 | 0 | Adequate/? | 0 |
|  | | WHOQOL-BREF (Malayalam version) | Sreedevi, A. et al., 2016 [28] | 0 | 0 | Adequate/? | Very good/- | 0 | 0 | 0 | 0 | Very good/+ | 0 |
|  | | WHOQOL-BREF 26 (Persian version) | Jahanlou, Alireza Shahab et al., 2011 [29] | Doubtful | 0 | 0 | Very good/? | 0 | Inadequate/? | 0 | 0 | 0 | 0 |
|  | | WHOQOL-BREF (Amharic version) | Reba, K. et al., 2019 [30] | Inadequate | 0 | Very good/- | Very good/- | 0 | 0 | 0 | 0 | Very good/+ | 0 |
| 4 | | RAND-12 | Maddigan, S. L. et al.,2004 [31] | 0 | 0 | 0 | 0 | 0 | 0 | 0 | 0 | Very good/+ | 0 |
| 5 | | HSQ 2.0 (Spanish version) | Morgan, Barbara S. et al.,2004 [32] | Doubtful | Doubtful/+ | 0 | Very good/- | 0 | Doubtful/? | 0 | 0 | 0 | 0 |
| 6 | | HUI2 | Maddigan, S. L. et al.,2004 [31] | 0 | 0 | 0 | 0 | 0 | 0 | 0 | 0 | Very good/+ | 0 |
| 7 | | HUI3 | -* | 0 | 0 | 0 | 0 | 0 | 0 | 0 | 0 | Very good/+ | 0 |
|  | | HUI3 | Maddigan, S. L. et al.,2004 [31] | 0 | 0 | 0 | 0 | 0 | 0 | 0 | 0 | Very good/+ | 0 |
|  | | HUI3 | Maddison, S. L. et al.,2006 [33] | 0 | 0 | 0 | 0 | 0 | 0 | 0 | 0 | Very good/+ | 0 |
| 8 | | EQ-5D | -* | 0 | 0 | 0 | Very good/+ | 0 | 0 | 0 | 0 | Very good/+ | 0 |
|  | | EQ-5D | Matza, L. S. et al.,2007 [34] | 0 | 0 | 0 | Very good/+ | 0 | 0 | 0 | 0 | Very good/+ | 0 |
|  | | EQ-5D | Lee, W. J. et al.,2012 [35] | 0 | 0 | 0 | 0 | 0 | 0 | 0 | 0 | Very good/+ | 0 |
| 9 | | EQ-5D-5L | -* | 0 | 0 | 0 | 0 | 0 | Very good/- | Very good/? | 0 | Very good/+ | 0 |
|  | | EQ-5D-5L | Al Sayah, F., et al.,2017 [36] | 0 | 0 | 0 | 0 | 0 | 0 | 0 | 0 | Very good/+ | 0 |
|  | | EQ-5D-5L | McClure, N. S. et al.,2018 [37] | 0 | 0 | 0 | 0 | 0 | 0 | Very good/? | 0 | 0 | 0 |
|  | | EQ-5D-5L | Wang, P. et al.,2016 [38] | 0 | 0 | 0 | 0 | 0 | 0 | 0 | 0 | Very good/+ | 0 |
|  | | EQ-5D-5L (Brunei-Malay version) | Koh, D. et al.,2016 [40] | 0 | 0 | 0 | 0 | 0 | Very good/- | 0 | 0 | Very good/+ | 0 |
|  | |  |  |  |  |  |  |  |  |  |  |  |  |
| 10 | | EQ-5D-3L | -* | 0 | 0 | 0 | 0 | Doubtful/- | 0 | 0 | 0 | 0 | 0 |
|  | | EQ-5D-3L | Konerding, U. et al.,2014 [39] | 0 | 0 | 0 | 0 | Very good/+ | 0 | 0 | 0 | 0 | 0 |
|  | | EQ-5D-3L (Finnish version) | Konerding, U. et al.,2014 [39] | 0 | 0 | 0 | 0 | Very good/+ | 0 | 0 | 0 | 0 | 0 |
|  | | EQ-5D-3L (German version) | Konerding, U. et al.,2014 [39] | 0 | 0 | 0 | 0 | Very good/+ | 0 | 0 | 0 | 0 | 0 |
|  | | EQ-5D-3L (Greek version) | Konerding, U. et al.,2014 [39] | 0 | 0 | 0 | 0 | Very good/+ | 0 | 0 | 0 | 0 | 0 |
|  | | EQ-5D-3L (Dutch) | Konerding, U. et al.,2014 [39] | 0 | 0 | 0 | 0 | Very good/+ | 0 | 0 | 0 | 0 | 0 |
|  | | EQ-5D-3L (Spanish) | Konerding, U. et al.,2014 [39] | 0 | 0 | 0 | 0 | Doubtful/- | 0 | 0 | 0 | 0 | 0 |
| 11 | | PACIC | -* | Adequate | Very good/+ | Adequate/? | Very good/+ | 0 | Very good/+ | 0 | Inadequate/? | 0 | 0 |
|  | | PACIC | Fan, J. et al.,2015 [41] | 0 | 0 | Very good/- | Very good/+ | 0 | 0 | 0 | 0 | 0 | 0 |
|  | | PACIC | Aung, E. et al.,2016 [42] | 0 | 0 | Adequate/? | 0 | 0 | Very good/+ | 0 | Inadequate/? | 0 | 0 |
|  | | PACIC (Malay version) | Abdul-Razak, S. et al.,2018 [45] | Adequate | Very good/+ | Adequate/? | Very good/+ | 0 | Very good/+ | 0 | 0 | 0 | 0 |
| 12 | | Short-version PACIC | Gugiu, P. C. et al.,2009 [43] | Inadequate | 0 | 0 | Very good/+ | 0 | Inadequate/- | 0 | 0 | 0 | 0 |
| 13 | | Modified-PACIC | Gugiu, C. et al.,2010 [44] | 0 | 0 | Very good/- | Very good/+ | 0 | 0 | 0 | 0 | 0 | 0 |
| 14 | | SF-36 | Hu, J, et al., 2010 [46] | 0 | 0 | 0 | Very good/+ | 0 | 0 | 0 | 0 | Very good/+ | 0 |
| 15 | | SF-12v2 | Wan, E. Y. F. et al.,2018 [47] | 0 | 0 | 0 | 0 | 0 | 0 | 0 | 0 | 0 | Very good/- |
| 16 | | MDQ | -* | Doubtful | Doubtful/? | 0 | Very good/? | 0 | Adequate/? | 0 | 0 | Very good/+ | 0 |
|  | | MDQ | France Talbot et al.,1997 [48] | Doubtful | Doubtful/? | 0 | Very good/? | 0 | 0 | 0 | 0 | Very good/+ | 0 |
|  | | MDQ (Hindi version) | Pawar, S. S. et al.,2013 [49] | Doubtful | 0 | 0 | Very good/+ | 0 | Adequate/? | 0 | 0 | 0 | 0 |
| 17 | | Diabetes Questionnaire | -* | 0 | 0 | Inadequate/? | 0 | 0 | 0 | 0 | 0 | 0 | 0 |
|  | | Diabetes Questionnaire | Gerber, B. et al.,2002 [50] | 0 | 0 | Inadequate/? | 0 | 0 | 0 | 0 | 0 | 0 | 0 |
|  | | Diabetes Questionnaire (Spanish version) | Gerber, B. et al.,2002 [50] | 0 | 0 | Inadequate/? | 0 | 0 | 0 | 0 | 0 | 0 | 0 |
| 18 | | PDQ-11 | Akohoue, S. A. et al.,2017 [51] | 0 | 0 | Adequate/? | Very good/- | 0 | 0 | 0 | 0 | 0 | 0 |
| 19 | | C-PDQ | Cheng, Li et al.,2018 [52] | Doubtful | Very good/+ | Adequate/? | Very good/+ | 0 | Very good/+ | 0 | Very good/+ | Very good/+ | 0 |
| 20 | | PRO-DM-Thai | Chuayruang, K. et al.,2015 [53] | Very good | Very good/+ | Very good/+ | Very good/+ | 0 | 0 | 0 | 0 | Very good/+ | 0 |
| 21 | | DQOL | -* | Doubtful | Doubtful/+ | Very good/- | Very good/? | 0 | Adequate/- | 0 | 0 | Very good/+ | 0 |
|  | | DQOL (Chinese version) | Alice Y. Cheng et al.,1999 [54] | 0 | 0 | 0 | Very good/? | 0 | Adequate/? | 0 | 0 | Very good/+ | 0 |
|  | | DQOL (Chinese version) | Jin, X. et al.,2017 [55] | 0 | 0 | Very good/- | Very good/+ | 0 | 0 | 0 | 0 | 0 | 0 |
|  | | DQOL (Iranian version) | Pakpour, A. H. et al.,2012 [56] | Adequate | 0 | Very good/- | Very good/+ | 0 | Adequate/- | 0 | 0 | 0 | 0 |
|  | | DQOL (Turkish version) | Yildirim, A. et al., 2007 [57] | Adequate | 0 | 0 | Very good/+ | 0 | Adequate/? | 0 | 0 | Very good/+ | 0 |
|  | | DQOL (Malay version) | Bujang, M. A. et al., 2017 [58] | Doubtful | Doubtful/+ | 0 | Very good/+ | 0 | 0 | 0 | 0 | 0 | 0 |
| 22 | | IRDQOL | Jahanlou, Alireza Shahab et al., 2011 [29] | 0 | 0 | 0 | Very good/? | 0 | Inadequate/? | 0 | 0 | 0 | 0 |
| 23 | | revised version of DQOL | Bujang, M. A. et al.,2018 [59] | Doubtful | 0 | Very good/+ | Very good/+ | 0 | 0 | 0 | 0 | 0 | 0 |
| 24 | | AsianDQOL | -* | Doubtful | Inadequate/+ | Adequate/? | Very good/+ | Doubtful/? | Adequate/? | 0 | 0 | 0 | 0 |
|  | | AsianDQOL | Goh, S. G., et al.,2015 [60] | Doubtful | Inadequate/+ | Very good/+ | Very good/+ | Doubtful/? | Adequate/? | 0 | 0 | 0 | 0 |
|  | | AsianDQOL (Malay version) | Goh, S. G., et al.,2015 [60] | Doubtful | Inadequate/+ | Very good/+ | Very good/+ | Very good/? | Adequate/? | 0 | 0 | 0 | 0 |
|  | | AsianDQOL (Chinese-mandarin version) | Goh, S. G., et al.,2015 [60] | Doubtful | Inadequate/+ | Adequate/? | Very good/+ | Doubtful/? | Adequate/? | 0 | 0 | 0 | 0 |
| 25 | | DQL-BCI (Polish version) | Dudzińska, M. et al.,2014 [61] | 0 | 0 | Inadequate/? | Very good/+ | 0 | Adequate/? | 0 | 0 | Very good/+ | 0 |
| 26 | | DQOL-B | Magwood, G. S.; Jenkins, C. et al.,2009 [62] | Very good | Doubtful/? | 0 | 0 | 0 | 0 | 0 | 0 | 0 | 0 |
| 27 | | QOLID | Nagpal, J. et al.,2010 [63] | Adequate | 0 | 0 | Very good/- | 0 | 0 | 0 | 0 | Very good/+ | 0 |
| 28 | | J-DQOL | Sato, F. et al.,2014 [64] | Inadequate | 0 | Inadequate/? | Very good/+ | 0 | Very good/- | 0 | 0 | Very good/? | 0 |
| 29 | | QOL questionnaire | Oobe, M. et al., 2007 [65] | Inadequate | 0 | 0 | Very good/+ | 0 | 0 | 0 | 0 | 0 | 0 |
| 30 | | DMQoL (Persian version) | Saffari, M. et al.,2019 [66] | 0 | 0 | Very good/- | Very good/+ | 9 | Very good/+ | 0 | 0 | Very good/+ | 0 |
| 31 | | MENQOL | Hasan, S. S. et al.,2014 [67] | 0 | 0 | 0 | Very good/+ | 0 | 0 | 0 | 0 | Very good/+ | 0 |
| 32 | | Diabetes-39 | -* | Inadequate | 0 | 0 | Very good/- | 0 | 0 | 0 | 0 | Very good/+ | 0 |
|  | | Diabetes-39 (Arabic version) | Khader, Y. S. et al.,2008[68] | Inadequate | 0 | 0 | Very good/+ | 0 | 0 | 0 | 0 | Very good/+ | 0 |
|  | | Diabetes-39 (Brazilian version) | de Queiroz, Flavia Alline et al.,2009[69] | Adequate | 0 | 0 | Very good/- | 0 | 0 | 0 | 0 | Very good/+ | 0 |
| 33 | | ADDQoL-19 | -* | Adequate | Doubtful/? | Inadequate/? | Very good/+ | 0 | Very good/+ | 0 | 0 | Very good/- | 0 |
|  | | ADDQoL-19 | Magwood, G. S. et al.,2009[62] | Very good | Doubtful/? | 0 | 0 | 0 | 0 | 0 | 0 | 0 | 0 |
|  | | ADDQoL-19 | Jannoo, Z. et al.,2015[70] | 0 | 0 | Very good/- | Very good/+ | 0 | 0 | 0 | 0 | 0 | 0 |
|  | | ADDQoL-19 | Fung, C. S. et al.,2016[71] | 0 | 0 | 0 | Very good/+ | 0 | 0 | 0 | 0 | Very good/- | 0 |
|  | | ADDQoL-19 | Abbatecola, A. M. et al.,2015[72] | 0 | 0 | Inadequate/? | Very good/+ | 0 | 0 | 0 | 0 | Very good/+ | 0 |
|  | | ADDQoL-19 (Malay version) | Jannoo, Z. et al.,2015[70] | 0 | 0 | Very good/- | Very good/+ | 0 | 0 | 0 | 0 | 0 | 0 |
|  | | ADDQoL (Chinese version) | Soon, S. S. et al., 2010[73] | Adequate | 0 | 0 | Very good/+ | 0 | Very good/+ | 0 | 0 | Very good/+ | 0 |
| 34 | | ADDQoL | -* | Inadequate | 0 | Adequate/? | Very good/+ | Adequate/? | Very good/+ | 0 | Very good/+ | 0 | 0 |
|  | | CN-ADDQoL | Kong, D. et al.,2011[74] | Adequate | 0 | Adequate/? | Very good/+ | 0 | 0 | 0 | 0 | 0 | 0 |
|  | | ADDQoL (Spanish version) | Lemon, S. C. et al.,2011[75] | 0 | 0 | 0 | Very good/+ | 0 | 0 | 0 | Very good/+ | 0 | 0 |
|  | | ADDQoL (Turkish version) | Demirci, H. et al.,2012[76] | 0 | 0 | 0 | Very good/+ | Adequate/? | 0 | 0 | 0 | 0 | 0 |
|  | | Malay ADDQOL | Kamarul Imran, M. et al.,2007[77] | Inadequate | 0 | Adequate/? | Very good/+ | 0 | Very good/+ | 0 | 0 | 0 | 0 |
| 35 | | *Elasy et al.* | Elasy, T. A. et al.,2000[78] | Very good | Doubtful/? | 0 | Very good/+ | 0 | 0 | 0 | 0 | Very good/+ | 0 |
| 36 | | DHP-1 | Meadows, K. A. et al.,2009[79] | 0 | 0 | 0 | Very good/? | 0 | 0 | 0 | 0 | Very good/+ | 0 |
| 37 | | DHP-3D | Mulhern, B. et al.,2017[80] | 0 | 0 | Inadequate/? | 0 | 0 | 0 | 0 | 0 | 0 | 0 |
| 38 | | DHP-5D | Mulhern, B. et al.,2017[80] | Inadequate | 0 | Doubtful/? | 0 | 0 | 0 | 0 | 0 | 0 | 0 |
| 39 | | DCP (Chinese version) | Li, J. et al.,2015[81] | Doubtful | 0 | 0 | Very good/- | 0 | 0 | 0 | Very good/- | Very good/+ | 0 |
| 40 | | DIMS (Chinese version) | Zhong Liu et al.,2006[82] | Doubtful | 0 | 0 | Very good/- | 0 | Inadequate/- | 0 | 0 | Very good/+ | 0 |
| 41 | | PGWB | Matza, L. et al.,2007[34] | 0 | 0 | 0 | Very good/+ | 0 | 0 | 0 | 0 | Very good/+ | 0 |
| 42 | | WBQ | -* | 0 | 0 | 0 | Very good/- | 0 | 0 | 0 | 0 | Adequate/? | 0 |
|  | | WBQ | Kolawole, B. A. et al.,2004[83] | 0 | 0 | 0 | Very good/+ | 0 | 0 | 0 | 0 | 0 | 0 |
|  | | WBQ | Kolawole, B. A. et al., 2009[27] | 0 | 0 | 0 | Very good/- | 0 | 0 | 0 | 0 | Adequate/? | 0 |
| 43 | | W-BQ28 | Speight, J. et al.,2012[84] | 0 | 0 | Very good/+ | Very good/+ | 0 | 0 | 0 | 0 | Very good/+ | 0 |
| 44 | | CES-Depression | -* | 0 | Doubtful/? | Adequate/? | Very good/? | 0 | Adequate/? | 0 | Very good/+ | Very good/+ | 0 |
|  | | CES-Depression | Rankin, S. H. et al.,1993[85] | 0 | Doubtful/? | 0 | Very good/? | 0 | 0 | 0 | 0 | Very good/+ | 0 |
|  | | CES-Depression | McHale, M. et al.,2008[86] | 0 | 0 | 0 | 0 | 0 | 0 | 0 | Very good/+ | 0 | 0 |
|  | | CES-Depression | Zhang, Y. Ting et al.,2015[87] | 0 | 0 | Adequate/? | Very good/+ | 0 | Adequate/? | 0 | 0 | Very good/+ | 0 |
|  | | CES-Depression | Carter, Jasmine et al.,2016[88] | 0 | 0 | Very good/+ | 0 | 0 | 0 | 0 | 0 | 0 | 0 |
| 45 | | Depression in Diabetes Self-Rating Scale | Kokoszka, A.,2008[89] | Doubtful | 0 | 0 | Adequate/? | 0 | Inadequate/? | 0 | 0 | 0 | 0 |
| 46 | | SCAD | McHale, M. et al.,2008[86] | 0 | 0 | 0 | 0 | 0 | 0 | 0 | Very good/- | 0 | 0 |
| 47 | | HADS | McHale, M. et al.,2008[86] | 0 | 0 | 0 | 0 | 0 | 0 | 0 | Very good/+ | 0 | 0 |
| 48 | | DMI | McHale, M. et al.,2008[86] | 0 | 0 | 0 | 0 | 0 | 0 | 0 | Very good/+ | 0 | 0 |
| 49 | | EDS | de Cock, Evi S. A. et al.,2011[90] | 0 | 0 | Very good/+ | 0 | 0 | 0 | 0 | 0 | 0 | 0 |
| 50 | | DCS | Zauszniewski, Jaclene A. et al.,2001[91] | 0 | 0 | Inadequate/? | Very good/+ | 0 | 0 | 0 | 0 | Very good/+ | 0 |
| 51 | | CUDOS-Chinese | Hsu, Lan-Fang et al.,2014[92] | Doubtful | Inadequate/+ | Very good/- | Very good/+ | 0 | Very good/+ | 0 | Very good/+ | Very good/+ | 0 |
| 52 | | CDDS-17 | Ting, R. Z. et al.,2011[93] | 0 | 0 | Adequate/? | Very good/+ | 0 | Adequate/? | 0 | 0 | 0 | 0 |
| 53 | | DDS Bahasa Indonesia | Farm, B. A. S. et al.,2017[94] | Doubtful | 0 | Inadequate/? | Very good/+ | 0 | 0 | 0 | 0 | 0 | 0 |
| 54 | | WHO-5 index | -* | Inadequate | 0 | Adequate/? | Very good/+ | 0 | 0 | 0 | 0 | Very good/+ | 0 |
|  | | WHO-5 index | Hajos, T. R. et al.,2013[95] | 0 | 0 | Very good/+ | Very good/+ | 0 | 0 | 0 | 0 | 0 | 0 |
|  | | WHO-5 index (Polish version) | Cichoń, E. et al.,2020[96] | Inadequate | 0 | Adequate/? | Very good/+ | 0 | 0 | 0 | 0 | Very good/+ | 0 |
| 55 | | PAID | Venkataraman, K. et al.,2015[97] | 0 | 0 | Very good/+ | Very good/+ | 0 | 0 | 0 | 0 | Very good/+ | 0 |
| 56 | | MY-PAID-20 | Jannoo, Z. et al,2019[98] | 0 | 0 | Very good/- | Very good/+ | 0 | 0 | 0 | Very good/+ | 0 | 0 |
| 57 | | B-PAID | Gross, C. C. et al.,2007[99] | 0 | 0 | 0 | Very good/- | 0 | 0 | 0 | 0 | Very good/+ | 0 |
| 58 | | PAID-K | Eom, Y. S. et al,2011[100] | Doubtful | 0 | 0 | Very good/? | 0 | 0 | 0 | 0 | 0 | 0 |
| 59 | | K-PAID | Lee, E. H. et al.,2014[101] | 0 | 0 | Very good/- | Very good/- | 0 | Very good/- | 0 | 0 | Very good/- | 0 |
| 60 | | K-PAID-5 | Lee, E. H. et al.,2014[101] | 0 | 0 | Very good/+ | Very good/+ | 0 | Very good/+ | 0 | 0 | Very good/+ | 0 |
| 61 | | Turkish PAID | Huis In, T. Veld E. M. J. et al.,2011[102] | 0 | 0 | 0 | Very good/+ | 0 | 0 | 0 | 0 | Very good/+ | 0 |
| 62 | | PAID (Greek version) | Papathanasiou, A. et al.,2014[103] | Inadequate | 0 | Adequate/? | Very good/+ | 0 | Very good/+ | 0 | Very good/+ | 0 | 0 |
| 63 | | SG-PAID-C | Siaw, M. et al.,2016[104] | 0 | 0 | Adequate/? | Very good/- | 0 | 0 | 0 | 0 | Very good/+ | 0 |
| 64 | | PAID (Spanish version) | Welch, G. et al.,2007[105] | 0 | 0 | Very good/- | 0 | Inadequate/? | 0 | 0 | 0 | 0 | 0 |
| 65 | | IR-PAID-20 | Arzaghi, S. M. et al.,2011[106] | 0 | 0 | Doubtful/? | Very good/+ | 0 | Inadequate/? | 0 | Inadequate/? | 0 | 0 |
| 66 | | PHQ-9 | -* | 0 | Very good/+ | Very good/+ | Very good/+ | 0 | Adequate/? | 0 | Very good/+ | Very good/+ | 0 |
|  | | PHQ-9 | Zhang, Yuying et al.,2013[107] | 0 | 0 | 0 | Very good/+ | 0 | Adequate/? | 0 | Very good/+ | 0 | 0 |
|  | | PHQ-9 | Janssen, E. P. et al.,2016[108] | 0 | 0 | Very good/+ | Very good/+ | 0 | 0 | 0 | Very good/+ | 0 | 0 |
|  | | PHQ-9 (Chichewa version) | Udedi, M. et al.,2019[109] | 0 | Very good/+ | 0 | Very good/+ | 0 | 0 | 0 | Very good/+ | 0 | 0 |
|  | | PHQ-9 (Romanian version) | Lupascu, N. et al.,2019[110] | 0 | 0 | 0 | Very good/+ | 0 | Adequate/? | 0 | 0 | Very good/+ | 0 |
| 67 | | SE-Type 2 | Bijl, J. V. et al.,1999[111] | 0 | Very good/? | 0 | Very good/ - | 0 | Very good/+ | 0 | 0 | Adequate/? | 0 |
| 68 | | DMSES | -* | Inadequate | Doubtful/? | Inadequate/? | Very good/- | 0 | Very good/+ | 0 | Very good/+ | Very good/+ | 0 |
|  | | DMSES | Sturt, Jackie et al.,2010[112] | 0 | Very good/+ | 0 | Very good/+ | 0 | Very good/+ | 0 | Very good/+ | Very good/+ | 0 |
|  | | K-DMSES | Lee, E. H. et al.,2015[113] | Inadequate | Adequate/+ | Very good/- | Very good/+ | 0 | Very good/+ | 0 | 0 | Very good/+ | 0 |
|  | | GR-DMSES | Fappa, E. et al.,2016[114] | Adequate | Very good/+ | Inadequate/? | Very good/+ | 0 | Very good/+ | 0 | 0 | 0 | 0 |
|  | | DMSES (Brazillian version) | Pace, A. E. et al.,2017[115] | Adequate | 0 | Very good/- | Very good/- | 0 | 0 | 0 | 0 | Very good/+ | 0 |
|  | | IT-DMSES | Messina, R. et al.,2018[116] | Doubtful | Doubtful/? | Inadequate/? | Very good/+ | 0 | 0 | 0 | 0 | Very good/+ | 0 |
| 69 | | DSEQ (Thai version) | Thojampa, S.,2018[117] | Doubtful | Adequate/+ | Very good/+ | 0 | 0 | 0 | 0 | 0 | 0 | 0 |
| 70 | | CDMSS-11 | Zheng, J. et al.,2018[118] | Doubtful | Inadequate/? | Adequate/? | Very good/+ | 0 | Very good/+ | 0 | 0 | 0 | 0 |
| 71 | | DSCAS | Sousa, Valmi D. et al.,2009[119] | Doubtful | Adequate/+ | 0 | 0 | 0 | 0 | 0 | 0 | 0 | 0 |
| 72 | | SDSCA | -* | Inadequate | Adequate/+ | Inadequate/? | Inadequate/? | 0 | Adequate/? | 0 | 0 | 0 | Inadequate/? |
|  | | SDSCA | Tolbert D.J. et al., 2000[120] | 0 | 0 | 0 | Inadequate/? | 0 | Adequate/? | 0 | 0 | 0 | Inadequate/? |
|  | | SDSCA (Turkish version) | Kav, Sultan et al.,2010[121] | Inadequate | Adequate/+ | Inadequate/? | Very good/- | 0 | 0 | 0 | 0 | 0 | 0 |
| 73 | | SDSCA | -* | Inadequate | Inadequate/+ | Adequate/? | Very good/- | 0 | Adequate/? | 0 | 0 | Very good/+ | 0 |
|  | | SDSCA-G | Kamradt, M. et al.,2014[122] | Doubtful | 0 | Very good/+ | Very good/- | 0 | 0 | 0 | 0 | Very good/+ | 0 |
|  | | SDSCA (Moroccan version) | Adarmouch, L. et al.,2016[123] | Doubtful | 0 | Adequate/? | Very good/- | 0 | Adequate/? | 0 | 0 | 0 | 0 |
|  | | SDSCA-Ar | Sukkarieh-Haraty, O. et al.,2016[124] | Inadequate | Inadequate/+ | Adequate/? | Very good/- | 0 | 0 | 0 | 0 | Very good/+ | 0 |
|  | | SDSCA-K | Choi, E. J. et al., 2011[125] | Doubtful | Doubtful/? | Very good/- | Very good/- | 0 | 0 | 0 | 0 | 0 | 0 |
| 74 | | INAAP-DM2 | Mendonça, S. C. B. et al.,2017[126] | Adequate | Very good/+ | 0 | 0 | 0 | 0 | 0 | 0 | 0 | 0 |
| 75 | | SCI-R | Khagram, L. et al.,2013[127] | 0 | 0 | Very good/+ | Very good/+ | 0 | 0 | 0 | 0 | Very good/+ | 0 |
| 76 | | DSSCI | García, A. A.,2011[128] | Adequate | 0 | 0 | Very good/? | 0 | Inadequate/? | 0 | 0 | Very good/+ | 0 |
| 77 | | SUGAAR | McCaskill, G. M. et al.,2016[129] | 0 | Very good/+ | 0 | 0 | 0 | 0 | 0 | 0 | Very good/+ | 0 |
| 78 | | DSES | -* | Doubtful | Adequate/+ | Very good/? | Very good/+ | 0 | Very good/- | 0 | 0 | 0 | 0 |
|  | | DSES | Sousa, Valmi D. et al.,2009[119] | Doubtful | Adequate/+ | 0 | 0 | 0 | 0 | 0 | 0 | 0 | 0 |
|  | | K-DSES | Chang, Sun Ju et al.,2014[130] | Adequate | 0 | Very good/? | Very good/+ | 0 | Very good/- | 0 | 0 | 0 | 0 |
| 79 | | Situational Self-Efficacy Scales (Spanish version) | Morgan, Barbara S. et al.,2004[32] | Doubtful | Doubtful/+ | 0 | Very good/? | 0 | Doubtful/? | 0 | 0 | 0 | 0 |
| 80 | | ESS | van der Heijden, M. M. et al.,2014[131] | 0 | 0 | Very good/- | Very good/+ | 0 | 0 | 0 | 0 | Very good/+ | 0 |
| 81 | | Self-Efficacy for Exercise 1 (Spanish version) | Morgan, Barbara S. et al.,2004[32] | Doubtful | Doubtful/+ | 0 | Very good/? | 0 | Doubtful/? | 0 | 0 | 0 | 0 |
| 82 | | Self-Efficacy for Exercise 2 (Spanish version) | Morgan, Barbara S. et al.,2004[32] | Doubtful | Doubtful/+ | 0 | Very good/? | 0 | Doubtful/? | 0 | 0 | 0 | 0 |
| 83 | | D-SMART® | Fain, J. A., 2007[132] | Doubtful | 0 | 0 | Very good/? | 0 | Adequate/? | 0 | 0 | 0 | 0 |
| 84 | | ES-SMBPA-2D | Nakawatase, Y. et al.,2007[133] | Adequate | Adequate/? | 0 | Very good/- | 0 | Very good/- | 0 | 0 | Very good/+ | 0 |
| 85 | | DSMS | Sousa, Valmi D. et al.,2009[119] | Doubtful | Adequate/+ | 0 | 0 | 0 | 0 | 0 | 0 | 0 | 0 |
| 86 | | DSMQ | -* | Doubtful | Adequate/+ | Very good/+ | Very good/+ | 0 | 0 | 0 | 0 | Very good/+ | 0 |
|  | | DSMQ (Thai version) | Thojampa, S. et al.,2017[134] | Adequate | Adequate/+ | 0 | Very good/+ | 0 | 0 | 0 | 0 | 0 | 0 |
|  | | DSMQ (Urdu version) | Bukhsh, A. et al.,2017[135] | Doubtful | 0 | Very good/+ | Very good/+ | 0 | 0 | 0 | 0 | Very good/+ | 0 |
| 87 | | V-DSMI | Dao-Tran, T. H. et al.,2017[136] | Inadequate | 0 | Very good/- | Very good/+ | 0 | 0 | 0 | 0 | 0 | 0 |
| 88 | | DSMI-20 | Lee, C. L. et al.,2016[137] | Adequate | Very good/+ | Adequate/? | Very good/+ | 0 | Adequate/? | 0 | 0 | Very good/+ | 0 |
| 89 | | DSMB-O | Seo, K. et al.,2017[138] | Doubtful | Adequate/+ | 0 | 0 | 0 | Very good/- | 0 | 0 | 0 | 0 |
| 90 | | SMP-T2D | Peyrot, M. et al.,2012[139] | Adequate | 0 | 0 | Very good/+ | 0 | Adequate/? | 0 | 0 | Very good/+ | Very good/+ |
| 91 | | IR-DES-28 | Mahjouri, M. Y. et al.,2012[140] | 0 | 0 | Inadequate/? | Very good/+ | 0 | Adequate/? | 0 | 0 | Very good/+ | 0 |
| 92 | | Hara et al. | Hara, Y. et al.,2014[141] | 0 | 0 | 0 | Very good/- | 0 | Adequate/- | 0 | 0 | Very good/+ | 0 |
| 93 | | DES-M | Ching, S. M. et al.,2020[142] | Adequate | 0 | Very good/? | Very good/- | 0 | 0 | 0 | 0 | Very good/- | 0 |
| 94 | | DES-SF | -* | Adequate | Very good/+ | Very good/- | Very good/- | 0 | Very good/- | 0 | 0 | 0 | 0 |
|  | | DES-SF (Brazilian Portuguese version) | Chaves, F. F. et al.,2017[143] | Adequate | Very good/+ | 0 | Very good/- | 0 | Very good/- | 0 | 0 | 0 | 0 |
|  | | DES-SF (Portuguese version) | Sousa, M. R. et al.,2019[144] | 0 | 0 | Very good/- | Very good/+ | 0 | Very good/- | 0 | 0 | 0 | 0 |
| 95 | | T2DHPS | -* | Inadequate | Very good/+ | Inadequate/? | Very good/- | 0 | Adequate/? | 0 | 0 | Very good/+ | 0 |
|  | | T2DHPS (Persian version) | Saffari, M. et al.,2015[145] | Inadequate | 0 | Inadequate/? | Very good/+ | 0 | Adequate/? | 0 | 0 | Very good/+ | 0 |
|  | | T2DHPS (Turkish version) | Yildiz, Esra et al.,2018[146] | Doubtful | Very good/+ | Very good/- | Very good/- | 0 | 0 | 0 | 0 | 0 | 0 |
| 96 | | DHPSC (Chinese version) | Wang, R. H. et al.,2012[147] | 0 | Very good/+ | Very good/- | Very good/+ | 0 | Very good/+ | 0 | 0 | 0 | 0 |
| 97 | | Health Belief Measures | Lewis, K. S. et al.,1990[148] | Inadequate | Doubtful/? | 0 | Very good/+ | 0 | 0 | 0 | 0 | Very good/- | 0 |
| 98 | | Given Health Belief Instrument (Spanish version) | Brown, S. A. et al., 2002[149] | Very good | Doubtful/? | 0 | Very good/- | 0 | 0 | 0 | 0 | 0 | 0 |
| 99 | | Health belief model scale (Turkish version) | Kartal, Asiye et al.,2007[150] | 0 | Adequate/+ | 0 | Very good/+ | 0 | Very good/+ | 0 | 0 | Very good/+ | 0 |
| 100 | | Diabetes Health Belief Measure | Lujan, J.,2008[151] | 0 | 0 | 0 | Very good/- | 0 | Inadequate/? | 0 | 0 | 0 | 0 |
| 101 | | PAM13 | Laranjo, L. et al.,2018[152] | Very good | 0 | Very good/+ | 0 | Very good/+ | 0 | 0 | 0 | 0 | 0 |
| 102 | | *Chernyak et al.* | Chernyak, N. et al.,2017[153] | Very good | Very good/+ | 0 | 0 | 0 | 0 | 0 | 0 | Very good/+ | 0 |
| 103 | | Diabetes Knowledge Questionnaire (Spanish version) | Morgan, Barbara S.et al.,2004[32] | Doubtful | Doubtful/+ | 0 | Very good/? | 0 | Doubtful/? | 0 | 0 | 0 | 0 |
| 104 | | DKQ-24 | Lujan, J.,2008[151] | 0 | 0 | 0 | Very good/+ | 0 | Inadequate/? | 0 | 0 | 0 | 0 |
| 105 | | DMKT | Padhy, M. et al.,2019[154] | Doubtful | Very good/+ | 0 | Very good/+ | 0 | 0 | 0 | 0 | Very good/+ | 0 |
| 106 | | PCSD-P | Matin, H. et al.,2019[155] | Doubtful | Very good/+ | Very good/- | Very good/+ | 0 | Very good/+ | 0 | 0 | 0 | 0 |
| 107 | | *Miller et al.* | Miller, Carla K. et al., 2000[156] | Doubtful | Adequate/+ | 0 | Very good/? | 0 | Doubtful/? | 0 | 0 | Very good/+ | 0 |
| 108 | | *Miller & Edwards* | Miller, C. et al., 2002[157] | Doubtful | Adequate/+ | 0 | 0 | 0 | 0 | 0 | Very good/+ | 0 | Very good/+ |
| 109 | | PDDC | Samuel-Hodge, C. D. et al., 2002[158] | Inadequate | 0 | 0 | Very good/? | 0 | Adequate/? | 0 | 0 | Very good/+ | 0 |
| 110 | | DRNK | Han, C. Y. et al., 2019[159] | Adequate | Very good/+ | 0 | Very good/- | 0 | Very good/+ | 0 | 0 | Very good/+ | 0 |
| 111 | | FCCHL (Norwegian version) | Finbråten, H. S. et al.,2018[160] | Doubtful | 0 | Very good/- | 0 | 0 | 0 | 0 | 0 | 0 | 0 |
| 112 | | KHLS-DM | Kang, Soo Jin et al., 2018[161] | Doubtful | Adequate/+ | Very good/+ | Very good/+ | 0 | Inadequate/? | 0 | 0 | 0 | 0 |
| 113 | | HLS-K | Lee, E. H. et al.,2018[162] | Inadequate | 0 | Very good/- | Very good/+ | 0 | 0 | 0 | 0 | Very good/+ | 0 |
| 114 | | HLS/SNS | Luo, H. et al.,2018[163] | 0 | 0 | Very good/- | Very good/+ | 0 | 0 | 0 | Very good/- | 0 | 0 |
| 115 | | Ashok et al. 1 | Ashok Kumar, M. et al.,2019[164] | 0 | 0 | Inadequate/? | Very good/+ | 0 | Very good/+ | 0 | 0 | Very good/+ | 0 |
| 116 | | Ashok et al. 2 | Ashok Kumar, M. et al.,2019[165] | 0 | 0 | Inadequate/? | Very good/+ | 0 | Very good/+ | 0 | 0 | Very good/+ | 0 |
| 117 | | HLS-EU-Q47 | Finbråten, H. S. et al.,2017[166] | Doubtful | 0 | Very good/- | Very good/+ | 0 | 0 | 0 | 0 | 0 | 0 |
| 118 | | DTSQ | -* | 0 | 0 | Inadequate/+ | Very good/+ | 0 | 0 | 0 | 0 | 0 | 0 |
|  | | DTSQ | Kolawole, B. A. et al.,2004[83] | 0 | 0 | 0 | Very good/+ | 0 | 0 | 0 | 0 | 0 | 0 |
|  | | DTSQ (Greek version) | Kontodimopoulos, N. et al.,2012[167] | 0 | 0 | Inadequate/+ | Very good/+ | 0 | 0 | 0 | 0 | 0 | 0 |
| 119 | | DiabMedSat | Brod, M. et al.,2009[168] | 0 | 0 | 0 | 0 | 0 | 0 | 0 | 0 | 0 | Very good/+ |
| 120 | | PTES | Azami, G, et al.,2018[169] | Inadequate | Doubtful/? | Very good/+ | Very good/+ | 0 | 0 | 0 | 0 | 0 | 0 |
|  | |  |  |  |  |  |  |  |  |  |  |  |  |
| 121 | | Demirtas et al. | Demirtaş, A. et al.,2017[170] | Adequate | Doubtful/? | Adequate/? | Very good/+ | 0 | Inadequate/? | 0 | Very good/+ | 0 | 0 |
| 122 | | FACIT-Fatigue Scale | Çinar, D. et al.,2018[171] | Doubtful | Adequate/+ | Very good/? | Very good/+ | 0 | Inadequate/? | 0 | 0 | 0 | 0 |
| 123 | | DTBQ | Ishii, H. et al.,2018[172] | 0 | 0 | Inadequate/? | Very good/+ | 0 | Very good/+ | 0 | 0 | 0 | 0 |
| 124 | | ITEQ | Moock, J. et al., 2010[173] | Very good | 0 | 0 | Adequate/- | 0 | 0 | 0 | 0 | Very good/+ | 0 |
| 125 | | IITQ | Rubin, R. R. et al.,2010[174] | 0 | 0 | 0 | Very good/+ | 0 | Very good/- | 0 | 0 | 0 | 0 |
| 126 | | ITAS | Soek, F. J. et al.,2007[175] | Inadequate | 0 | 0 | Very good/- | 0 | 0 | 0 | 0 | Very good/+ | 0 |
| 127 | | C-ITAS-HK | Lee, K. P.,2018[176] | Adequate | Very good/+ | Adequate/? | Very good/- | 0 | Adequate/? | 0 | 0 | Very good/+ | 0 |
| 128 | | BITQ (Turkish version) | Saritas, S. C. et al.,2019[177] | Doubtful | 0 | Very good/- | Very good/+ | 0 | Adequate/? | 0 | 0 | 0 | 0 |
| 129 | | Ch-ASIQ | Fu, S. N. et al.,2013[178] | Doubtful | Very good/+ | Adequate/? | Very good/- | 0 | 0 | 0 | 0 | 0 | 0 |
| 130 | | MIAS | Osborn, C. Y. et al.,2016[179] | 0 | 0 | 0 | Very good/- | 0 | 0 | 0 | Very good/+ | Very good/+ | 0 |
| 131 | | IMDSES (Brazilian version) | Dos Santos, R. B. P. et al.,2018[180] | 0 | 0 | 0 | Very good/+ | 0 | 0 | 0 | Very good/- | Very good/+ | 0 |
| 132 | | ITSQ | Anderson, Roger T. et al.,2004[181] | Inadequate | 0 | 0 | Very good/+ | 0 | Adequate/- | 0 | 0 | Very good/+ | 0 |
| 133 | | MMAS (Thai version) | Sakthong, P. et al., 2009[182] | Doubtful | 0 | Very good/? | Very good/- | 0 | Very good/+ | 0 | 0 | 0 | 0 |
| 134 | | modiﬁed 4-item Morisky–Green–Levine Medication Adherence Scale | Wang, Y. et al., 2012[183] | 0 | 0 | Inadequate/? | Very good/- | 0 | 0 | 0 | 0 | 0 | 0 |
| 135 | | MMAS-8 | -* | Adequate | 0 | Adequate/? | Very good/- | 0 | Very good/+ | 0 | Very good/? | Very good/- | 0 |
|  | | MMAS-8 (Korean version) | Lee, W. Y. et al.,2013[184] | Adequate | 0 | Very good/- | Very good/- | 0 | Very good/+ | 0 | Very good/? | 0 | 0 |
|  | | MMAS-8 (Chinese version) | Wang, J. et al.,2013[185] | 0 | 0 | Adequate/? | Very good/- | 0 | Very good/+ | 0 | 0 | Very good/+ | 0 |
|  | | MMAS-8 (French version) | Zongo, A. et al.,2016[186] | 0 | 0 | 0 | 0 | 0 | 0 | 0 | 0 | Very good/- | 0 |
|  | | MMAS-8 (French version) | Zongo, A. et al., 2016[187] | 0 | 0 | Adequate/? | Very good/- | 0 | 0 | 0 | 0 | 0 | 0 |
| 136 | | MGLS (Indonesian version) | Kristina, S. A. et al.,2019[188] | Doubtful | 0 | 0 | Very good/- | 0 | Very good/+ | 0 | Very good/+ | Very good/+ | 0 |
| 137 | | OHA-Q | Ishii, H. et al.,2012[189] | 0 | 0 | Inadequate/? | Very good/+ | 0 | 0 | 0 | 0 | 0 | 0 |
| 138 | | DMSRQ | Peyrot, M. et al.,2012[190] | 0 | 0 | Doubtful/? | Very good/+ | 0 | Inadequate/? | 0 | 0 | Very good/+ | 0 |
| 139 | | Medical Prescription Knowledge questionnaire | Prado-Aguilar, C. A. et al., 2009[191] | Doubtful | Very good/+ | 0 | Adequate/? | 0 | 0 | 0 | Very good/+ | 0 | 0 |
| 140 | | Attitude Scale | Prado-Aguilar, C. A. et al., 2009[191] | Doubtful | Very good/+ | 0 | Adequate/? | 0 | 0 | 0 | Very good/+ | 0 | 0 |
| 141 | | BMQ-f | Fall, E. et al.,2014[192] | Inadequate | 0 | Very good/- | Very good/+ | 0 | 0 | 0 | 0 | 0 | 0 |
| 142 | | MALMAS | Chung, Wen Wei et al.,2015[193] | 0 | 0 | 0 | Very good/- | 0 | Adequate/? | 0 | 0 | Very good/+ | 0 |
| 143 | | MAT OADs | Fischetti, Natalie Ann,2014[194] | 0 | Doubtful/+ | 0 | Very good/+ | 0 | 0 | 0 | Very good/+ | 0 | 0 |
| 144 | | MAT insulin | Fischetti, Natalie Ann,2014[194] | 0 | Doubtful/+ | 0 | Very good/- | 0 | 0 | 0 | Very good/+ | 0 | 0 |
| 145 | | ARMS-K | Kim, Chun-Ja et al.,2016[195] | Inadequate | 0 | Adequate/? | Very good.+ | 0 | 0 | 0 | 0 | Very good/+ | 0 |
| 146 | | Diabetes Medication System Rating Questionnaire Short-Form | Peyrot, M. et al.,2014[196] | Inadequate | 0 | 0 | Very good/+ | 0 | Inadequate/? | 0 | Very good/+ | Very good/+ | 0 |
| 147 | | SR-4 (French version) | Zongo, A. et al.,2016[186] | 0 | 0 | 0 | 0 | 0 | 0 | 0 | 0 | Very good/- | 0 |
| 148 | | *Zongo et al. 1* | Zongo, A. et al.,2016[186] | 0 | 0 | 0 | 0 | 0 | 0 | 0 | 0 | Very good/- | 0 |
| 149 | | *Zongo et al. 2* | Zongo, A. et al.,2016[186] | 0 | 0 | 0 | 0 | 0 | 0 | 0 | 0 | Very good/- | 0 |
| 150 | | HPQ | -* | 0 | 0 | Very good/- | Very good/+ | 0 | 0 | 0 | 0 | Very good/+ | 0 |
|  | | HPQ (Cyprus version) | Kawata, A. K. et al.,2016[197] | 0 | 0 | Very good/+ | Very good/+ | 0 | 0 | 0 | 0 | Very good/+ | 0 |
|  | | HPQ | Kawata, A. K. et al.,2016[197] | 0 | 0 | Very good/- | Very good/+ | 0 | 0 | 0 | 0 | Very good/+ | 0 |
| 151 | | CHI (Filipino version) | De Mesa, U. R. et al.,2017[198] | Doubtful | 0 | 0 | Very good/- | 0 | 0 | 0 | Very good/- | 0 | 0 |
| 152 | | FH-15 (Chinese version) | Liu, Y. Q. et al.,2018[199] | 0 | Adequate/+ | Very good/- | Very good/+ | 0 | Adequate/? | 0 | 0 | 0 | 0 |
| 153 | | K-DSC-R | Lee, E. H. et al.,2014[200] | 0 | 0 | Very good/+ | Very good/- | 0 | 0 | 0 | 0 | Very good/+ | 0 |
| 154 | | DSC-R | Arbuckle, R. A. et al.,2009[201] | 0 | 0 | Very good/+ | Very good/- | 0 | 0 | 0 | 0 | 0 | Inadequate/? |
| 155 | | *Naegeli et al.* | Naegeli, A. N. et al.,2010[202] | 0 | 0 | Very good/+ | Very good/+ | 0 | Very good/- | 0 | 0 | Very good/+ | 0 |
| 156 | | Barriers to Fat Reduction Scale (Spanish version) | Morgan, Barbara S. et al.,2004[32] | Doubtful | Doubtful/+ | 0 | Very good/- | 0 | Doubtful/? | 0 | 0 | 0 | 0 |
| 157 | | Barriers to Exercise Checklist (Spanish version) | Morgan, Barbara S. et al.,2004[32] | Doubtful | Doubtful/+ | 0 | Very good/? | 0 | Doubtful/? | 0 | 0 | 0 | 0 |
| 158 | | Food Habits Questionnaire (Spanish version) | Morgan, Barbara S. et al.,2004[32] | Doubtful | Doubtful/+ | 0 | Very good/? | 0 | Doubtful/? | 0 | 0 | 0 | 0 |
| 159 | | DDRQOL | Sato, E. et al.,2004[203] | Inadequate | 0 | Very good/? | Very good/+ | 0 | Very good/- | 0 | 0 | Very good/+ | 0 |
| 160 | | DDRQOL-R | Sato, E. et al.,2017[204] | Adequate | 0 | Adequate/? | Very good/+ | 0 | Very good/- | 0 | Very good/+ | 0 | 0 |
| 161 | | *Sato et al.* | Sato, E. et al.,2017[204] | Adequate | 0 | 0 | Very good/+ | 0 | Very good/- | 0 | Very good/+ | 0 | 0 |
| 162 | | IW-SP | Hayes, R. P. et al.,2015[205] | Doubtful | 0 | Inadequate/? | Very good/+ | 0 | Very good/+ | 0 | 0 | Very good/+ | 0 |
| 163 | | Motiva.Diaf-DM2 questionnaire | Payo, Ruben Martin et al.,2018[206] | 0 | 0 | Very good/- | Very good/+ | 0 | Adequate/? | 0 | 0 | 0 | 0 |
| 164 | | HAPA-based PA inventory | Rohani, H., et al.,2016[207] | 0 | Very good/+ | Adequate/+ | Very good/- | 0 | Very good/+ | 0 | 0 | 0 | 0 |
| 165 | | STOP-Bang questionnaire | Teng, Y. et al.,2018[208] | 0 | 0 | 0 | 0 | 0 | 0 | 0 | Very good/+ | 0 | 0 |
| 166 | | PROMIS - Sleep Disturbance instrument | Donovan, L. M. et al.,2020[209] | 0 | 0 | 0 | 0 | 0 | 0 | 0 | 0 | Very good/- | Very good/? |
| 167 | | PROMIS - Sleep Related Impairment instrument | Donovan, L. M. et al.,2020[209] | 0 | 0 | 0 | 0 | 0 | 0 | 0 | 0 | Very good/- | Very good/? |
| 168 | | RSSM-Farsi | Shakibazadeh, E. et al.,2012[210] | Doubtful | 0 | Adequate/- | Very good/- | 0 | Very good/+ | 0 | 0 | 0 | 0 |
| 169 | | DFBC (Japanese version) | Hara, Y. et al.,2013[211] | Doubtful | 0 | Adequate/- | Very good/+ | 0 | Very good/+ | 0 | 0 | 0 | 0 |
| 170 | | FSS-AA T2DM | Littlewood, K. et al.,2015[212] | Doubtful | 0 | Adequate/? | Very good/+ | 0 | Adequate/? | 0 | 0 | Very good/+ | 0 |
| 171 | | HCCQ-P | Matin, H. et al., 2019[213] | Doubtful | Very good/+ | Very good/- | Very good/+ | 0 | Very good/+ | 0 | 0 | 0 | 0 |
| 172 | | The Diabetes Family Support and Conflict Scale (Turkish version) | Sofulu, F. et al.,2017[214] | Doubtful | Adequate/+ | Adequate/? | Very good/+ | 0 | 0 | 0 | 0 | 0 | 0 |
| 173 | | SSS-J | Kato, A. et al.,2014[215] | Doubtful | Doubtful/? | Very good/- | Very good/+ | 0 | Adequate/? | 0 | 0 | Very good/+ | 0 |
| 174 | | DSAS-2 | Browne, J. L et al.,2016[216] | Inadequate | 0 | Inadequate/? | Very good/+ | 0 | 0 | 0 | 0 | Very good/+ | 0 |
| 175 | | Relationship Consciousness of Japanese Patients with Type 2 Diabetes Mellitus Scale | Koike, M. et al.,2019[217] | Doubtful | Adequate/+ | Adequate/? | Very good/+ | 0 | 0 | 0 | 0 | Very good/+ | 0 |
| 176 | | ADS | -* | Inadequate | 0 | Inadequate/? | Very good/+ | 0 | Inadequate/? | 0 | 0 | Very good/+ | 0 |
|  | | ADS (Japanese version) | Hara, Y. et al.,2011[218] | Inadequate | 0 | 0 | Very good/- | 0 | Inadequate/? | 0 | 0 | 0 | 0 |
|  | | ADS (Korean version) | Lee, E. H. et al.,2015[219] | Inadequate | 0 | Inadequate/? | Very good/+ | 0 | 0 | 0 | 0 | Very good/+ | 0 |
| 177 | | DAAS | Ebrahimi, H. et al.,2016[220] | Doubtful | Adequate/+ | Adequate/? | Very good/+ | 0 | 0 | 0 | 0 | 0 | 0 |
| 178 | | IR-DAS-3 | Mahjouri, M. Y. et al.,2011[221] | 0 | 0 | 0 | Very good/? | 0 | Inadequate/? | 0 | 0 | 0 | 0 |
| 179 | | GCQ | Persson, Lars-Olof et al.,2013[222] | 0 | 0 | Adequate/? | Very good/+ | 0 | 0 | 0 | 0 | Very good/+ | 0 |
| 180 | | DIAB-Q | Traina, S. B. et al.,2016[223] | Very good | 0 | Very good/+ | Very good/- | 0 | Very good/- | 0 | 0 | Very good/+ | 0 |
| 181 | | S-BRCS | Martinez, Nelda C. et al.,2011[224] | Inadequate | 0 | Adequate/? | Very good+ | 0 | Doubtful/? | 0 | 0 | 0 | 0 |
| 182 | | DPMD | Golin C.E. et al., 2001[225] | Doubtful | Adequate/+ | 0 | Very good/+ | 0 | Adequate/? | 0 | 0 | Very good/- | 0 |
| 183 | | DOQ | -* | Doubtful | Adequate/+ | 0 | Very good/- | 0 | 0 | 0 | Very good/+ | Very good/+ | 0 |
|  | | DOQ | Hearnshaw, H. et al., 2007[226] | Doubtful | Adequate/+ | 0 | Very good/+ | 0 | 0 | 0 | Very good/+ | Very good/+ | 0 |
|  | | DOQ (Dutch version) | Vandekerckhove, M. et al.,2009[227] | Doubtful | 0 | 0 | Very good/- | 0 | 0 | 0 | 0 | Very good/+ | 0 |
| 184 | | DOQ-30 | Pilv, L. et al.,2016[228] | 0 | 0 | Adequate/? | Very good/- | 0 | 0 | 0 | 0 | 0 | 0 |
| 185 | | DPSS | Hill-Briggs, Felicia et al.,2007[229] | 0 | 0 | Inadequate/? | Very good/+ | 0 | 0 | 0 | 0 | Very good/+ | 0 |
| 186 | | CIRS (Thai version) | Manit, A. et al.,2011[230] | Adequate | Adequate/+ | 0 | Very good/? | 0 | 0 | 0 | 0 | 0 | 0 |
| 187 | | IPQ-R | Abubakari, Abdul-Razak et al.,2012[231] | 0 | 0 | Very good/- | Very good/- | 0 | Inadequate/- | 0 | 0 | 0 | 0 |
| 188 | | CHES-Q | Traina, S. B. et al.,2015[232] | Inadequate | 0 | Very good/- | 0 | 0 | Adequate/? | 0 | 0 | Very good/+ | 0 |
| 189 | | MBIPQ | Chew, B. H. et al.,2017[233] | 0 | 0 | 0 | 0 | Inadequate/? | Very good/- | 0 | 0 | Very good/+ | 0 |
| 190 | | DFS | Egede, L. E. et al.,2010[234] | Doubtful | 0 | 0 | Very good/+ | 0 | 0 | 0 | 0 | Very good/+ | 0 |

0: Measurement property was not assessed by the study/ not applicable to be assessed

*Pooled results of different language versions of the same PROM

**Abbreviations:**

WHOQOL-100: World Health Organisation quality of life questionnaire; WHOQOL-BREF/ WHOQOL-BREF 26: abbreviated World Health Organization Quality of Life; WHOQOL-BREF 26; HSQ 2.0: Health Status Questionnaire 2.0; HUI2: Health Utilities Index Mark 2; HUI3: Health Utilities Index Mark 3; EQ-5D: EuroQol 5-Dimension; EQ-5D-5L: EQ-5D with 5 level scale; EQ-5D-3L: EQ-5D with 3 level scale; PACIC: Patient Assessment of Chronic Illness Care; SF-36: 36-Item Short Form Survey; SF-12v2: Short Form-12 Health Survey version 2; MDQ: Multidimensional Diabetes Questionnaire; PDQ-11: Personal Diabetes Questionnaire; C-PDQ: Chinese version of PDQ; PRO-DM-Thai: instrument for patient-reported outcomes in Thai patients with type 2 diabetes mellitus; DQOL: Diabetes Quality-of-Life Measure; IRDQOL: Iranian diabetes quality of life; AsianDQOL: Asian Diabetes Quality of Life; DQL-BCI: Diabetes Quality of Life - Brief Clinical Inventory; DQOL-B: Diabetes Quality of Life Brief Clinical Inventory; QOLID: Quality of Life Instrument for Indian Diabetes patients; J-DQOL: Japanese version of the Diabetes Quality-Of-Life; QOL: quality of life; DMQoL: Diabetes-Mellitus Specific Quality of Life; MENQOL: Menopause-specific Quality of Life; ADDQoL-19: 19-item Audit of Diabetes-Dependent Quality of Life; CN-ADDQoL: Adaptation of the ADDQoL questionnaire to people with diabetes in China; DHP-1: Diabetes Health Profile; DHP-3D: Diabetes Health Proﬁle–3 Dimension; DHP-5D: Diabetes Health Proﬁle–5 Dimension; DCP: Diabetes Care Profile; DIMS: diabetes impact measurement scales; PGWB: Psychological General Well-Being Questionnaire; WBQ: Well-being Questionnaire; W-BQ28: 28-item Well-Being Questionnaire; CES-Depression: Center for Epidemiological Studies Depression scale; SCAD: Silverstone Concise Assessment for Depression; HADS: Hospital Anxiety and Depression Scale; DMI: Depression in the Medically Ill Questionnaire; EDS: Edinburgh Depression Scale; DCS: Depressive Cognition Scale; CUDOS-Chinese: Mandarin Chinese Version of the Clinically Useful Depression Outcome Scale; CDDS-17: Chinese version of the Diabetes Distress Scale; DDS Bahasa Indonesia: Indonesian Diabetes Distress Scale; WHO-5 well-being index: 5-item World Health Organization well-being index; PAID: Problem Areas in Diabetes; MY-PAID-20: Malaysian version of PAID; B-PAID: Brazilian version of PAID; PAID-K: Korean version of PAID; short form Korean version of PAID; IR-PAID-20: Iranian version of PAID; PHQ-9: Patient Health Questionnaire-9; SE-Type 2: self-efficacy scale for patients with type 2 diabetes mellitus; DMSES: diabetes management self-efficacy scale; K-DMSES: Korean version of DMSES; GR-DMSES Greek version of DMSES; IT-DMSES: Italian version of DMSES; CDMSS-11: Chinese version of Diabetes Medication Self-efficacy Scale; DSCAS: Diabetes Self-Care Agency Scale; SDSCA: Summary of diabetes self-care activities measure; SDSCA-G: German version of SDSCA; SDSCA-Ar: Arabic version of SDSCA; SDSCA-K: Korean version of SDSCA; INAAP-DM2: Self-care Assessment Instrument for patients with type 2 diabetes mellitus; SCI-R: Self-Care Inventory-Revised; DSSCI: Diabetes Symptom Self-Care Inventory; SUGAAR: Self-Care Utility Geriatric African-American Rating; DSES: Diabetes Self-efficacy Scale; K-DSES: Korean version of DSES; ESS: Exercise Self-efficacy Scale; D-SMART®: Diabetes Self-management Assessment Report Tool; ES-SMBPA-2D: evaluation scale for self-management behavior related to physical activity of type 2 diabetic patients; DSMS: Diabetes Self-Management Scale; DSMQ: Diabetes Self-management Questionnaire; V-DSMI: Vietnamese version of diabetes self-management instrument; DSMI-20: Diabetes Self-Management Instrument Short Form; DSMB-O: Diabetes Self-Management Behavior for Older Koreans; SMP-T2D: self-management profile for type 2 diabetes; IR-DES-28: Iranian version of Diabetes Empowerment Scale; DES-M: diabetes empowerment scale; DES-SF: Diabetes Empowerment Scale - Short Form; T2DHPS: Type 2 Diabetes and Health Promotion Scale; DHPSC: diabetes health promotion self-care scale; PAM13: Patient Activation Measure; DKQ-24: Diabetes Knowledge Questionnaire; DMKT: Diabetes Mellitus Knowledge Test; PCSD-P: Persian Version of Perceived Competence Scale for Diabetes; PDDC: measure of perceived diabetes and dietary competence; DRNK: diabetes-related nutrition knowledge questionnaire; FCCHL: Functional, Communicative, and Critical Health Literacy Scale; KHLS-DM: Korean Health Literacy Scale for Diabetes Mellitus; HLS-K: Health Literacy Scale; HLS/SNS: Health Literacy Scale/Subjective Numeracy Scale; HLS-EU-Q47: European Health Literacy Survey Questionnaire; DTSQ: Diabetes Treatment Satisfaction Questionnaire; DiabMedSat: Diabetes Medication Satisfaction measure; PTES: Perceived Therapeutic Efficacy Scale; DTBQ: Diabetic Treatment Burden Questionnaire; ITEQ: insulin treatment experience questionnaire; IITQ: inhaled insulin treatment questionnaire; ITAS: insulin treatment appraisal scale; C-ITAS-HK: Insulin Treatment Appraisal Scale in Hong Kong primary care patients; BITQ: barriers to insulin treatment questionnaire; Ch-ASIQ: Chinese Attitudes to Starting Insulin Questionnaire; MIAS: Morisky Medication Adherence Scale adapted to specify insulin adherence; IMDSES: Insulin Management Diabetes Self-Efficacy Scale; ITSQ: Insulin Treatment Satisfaction Questionnaire; MMAS/MMAS-8: 8-item Morisky Medication Adherence Scale; MGLS: 4 items Morisky Green Levine Adherence Scale; OHA-Q: Oral Hypoglycemic Agent Questionnaire; DMSRQ: Diabetes Medication System Rating Questionnaire; BMQ-f: French version of the Beliefs about Medicines Questionnaire; MALMAS: Malaysian Medication Adherence Scale; MAT OADs: Measurement of Adherence to Drug Therapy in Diabetes Mellitus – Oral Antidiabetics; MAT insulin: Measurement of Adherence to Drug Therapy in Diabetes Mellitus – Insulin Therapy; ARMS-K: Korean version of the Adherence to Refills and Medications Scale; SR-4: self-report with 4 items; HPQ: Hypoglycemia Perspectives Questionnaire; CHI: Clarke Hypoglycemia Index; FH-15: Chinese version of the new fear of hypoglycemia scale; K-DSC-R: Korean version of Diabetes Symptom Checklist-Revised; DSC-R: Diabetes Symptom Checklist-Revised; DDRQOL: diabetes diet-related quality-of-life scale; DDRQOL-R: revised and short form versions of diabetes diet-related quality of life scale; IW-SP: Impact of Weight on Self-Perceptions Questionnaire; HAPA-based PA inventory: health action process approach (HAPA)-based PA inventory; RSSM-Farsi: Iranian version of Resources and Support for Chronic illness Self-management scale; DFBC: Diabetes Family Behavior Checklist; FSS-AA T2DM: Family Support Scale Adapted for African American Women with Type 2 Diabetes Mellitus; HCCQ-P: Persian health care climate questionnaire; SSS-J: Japanese version of the self-stigma scale; DSAS-2: Type 2 Diabetes Stigma Assessment Scale; ADS: Appraisal of Diabetes Scale; DAAS: Diabetes Adjustment Assessment Scale; IR-DAS-3: Iranian Diabetes Attitude Scale; GCQ: General Coping Questionnaire; DIAB-Q: 17-item Diabetes Intention, Attitude, and Behavior Questionnaire; S-BRCS: Spanish Brief Religious Coping Scale; DPMD: diabetes-specific measure of patient desire to participate in medical decision making; DOQ: Diabetes Obstacles Questionnaire; DOQ-30: short version of the diabetes obstacles questionnaire; DPSS: Diabetes Problem-Solving Scale; CIRS: Chronic Illness Resources Survey; IPQ-R: Revised Illness Perception Questionnaire; CHES-Q: 14-item Current Health Satisfaction Questionnaire; MBIPQ: Malay version of the brief illness perception questionnaire; DFS: 12-item diabetes fatalism scale

**Supplementary Table 6. Evidence synthesis of measurement properties for each PROM (based on COSMIN Risk of Bias checklist).**

|  | | **Overall level of evidence for measurement properties** | | | | | | | | | |
| --- | --- | --- | --- | --- | --- | --- | --- | --- | --- | --- | --- |
| **PROM** | **No. of studies** | **PROM development** | **Content validity** | **Structural validity** | **Internal consistency** | **Cross-cultural validity\ Measurement invariance** | **Reliability** | **Measurement error** | **Criterion validity** | **Hypotheses testing for construct validity** | **Responsiveness** |
| **General impact on quality of life** | | | | | | | | | | |  |
| WHOQOL-100 | 1 | very low | 0 | 0 | +/moderate | 0 | ?/very low | 0 | 0 | +/moderate | +/moderate |
| WHOQOL-BREF* | 4 | very low | 0 | ?/moderate | -/moderate | 0 | ?/very low | 0 | 0 | ?/low | 0 |
| WHOQOL-BREF | 1 | 0 | 0 | 0 | -/moderate | 0 | 0 | 0 | 0 | ?/low | 0 |
| WHOQOL-BREF (Malayalam version) | 1 | 0 | 0 | ?/moderate | -/high | ^a^ | 0 | 0 | 0 | +/high | 0 |
| WHOQOL-BREF 26 (Persian version) | 1 | low | 0 | 0 | ?/high | ^a^ | ?/very low | 0 | 0 | 0 | 0 |
| WHOQOL-BREF (Amharic version) | 1 | very low | 0 | -/high | -/high | ^a^ | 0 | 0 | 0 | +/high | 0 |
| RAND-12 | 1 | 0 | 0 | 0 | 0 | 0 | 0 | 0 | 0 | +/high | 0 |
| HSQ 2.0 (Spanish version) | 1 | very low | +/very low | 0 | -/low | ^a^ | ?/very low | 0 | 0 | 0 | 0 |
| HUI2 | 1 | 0 | 0 | 0 | 0 | 0 | 0 | 0 | 0 | +/high | 0 |
| HUI3 | 2 | 0 | 0 | 0 | 0 | 0 | 0 | 0 | 0 | +/high | 0 |
| EQ-5D* | 2 | 0 | 0 | 0 | +/high | 0 | 0 | 0 | 0 | +/high | 0 |
| EQ-5D | 1 | 0 | 0 | 0 | +/high | 0 | 0 | 0 | 0 | +/high | 0 |
| EQ-5D (Korean version) | 1 | 0 | 0 | 0 | 0 | 0 | 0 | 0 | 0 | +/high | 0 |
| EQ-5D-5L* | 4 | 0 | 0 | 0 | 0 | 0 | -/high | ?/high | 0 | ?high | 0 |
| EQ-5D-5L | 3 | 0 | 0 | 0 | 0 | 0 | 0 | ?/high | 0 | ?high | 0 |
| EQ-5D-5L (Brunei-Malay version) | 1 | 0 | 0 | 0 | 0 | 0 | -/high | 0 | 0 | +/high | 0 |
| EQ-5D-3L* | 7 | 0 | 0 | 0 | 0 | -/low | 0 | 0 | 0 | -/high | 0 |
| EQ-5D-3L | 2 | 0 | 0 | 0 | 0 | +/high | 0 | 0 | 0 | -/high | 0 |
| EQ-5D-3L (Finnish version) | 1 | 0 | 0 | 0 | 0 | +/high | 0 | 0 | 0 | 0 | 0 |
| EQ-5D-3L (German version) | 1 | 0 | 0 | 0 | 0 | +/high | 0 | 0 | 0 | 0 | 0 |
| EQ-5D-3L (Greek version) | 1 | 0 | 0 | 0 | 0 | +/high | 0 | 0 | 0 | 0 | 0 |
| EQ-5D-3L (Dutch) | 1 | 0 | 0 | 0 | 0 | +/high | 0 | 0 | 0 | 0 | 0 |
| EQ-5D-3L (Spanish) | 1 | 0 | 0 | 0 | 0 | -/low | 0 | 0 | 0 | 0 | 0 |
| PACIC* | 3 | moderate | +/high | ?/low | +/high | 0 | +/high | 0 | ?/very low | 0 | 0 |
| PACIC | 2 | 0 | 0 | +/moderate | +/high | 0 | +/high | 0 | ?/very low | 0 | 0 |
| PACIC (Malay version) | 1 | moderate | +/high | ?/low | +/high | ^a^ | +/high | 0 | 0 | 0 | 0 |
| Short-version PACIC | 1 | very low | 0 | 0 | +/high | 0 | -/very low | 0 | 0 | 0 | 0 |
| Modified-PACIC | 1 | 0 | 0 | -/high | +/high | 0 | 0 | 0 | 0 | 0 | 0 |
| SF-36 | 1 | 0 | 0 | 0 | +/moderate | 0 | 0 | 0 | 0 | +/moderate | 0 |
| SF-12v2 | 1 | 0 | 0 | 0 | 0 | 0 | 0 | 0 | 0 | 0 | -/high |
| **Diabetes-specific impact on quality of life** | | | | | | | | | | |  |
| PRO-DM-Thai | 1 | high | +/high | +/high | +/high | ^a^ | 0 | 0 | 0 | +/high | 0 |
| DQOL* | 5 | low | +/low | -/high | ?/moderate | 0 | -/low | 0 | 0 | +/moderate | 0 |
| DQOL (Chinese version) | 1 | 0 | 0 | 0 | ?/moderate | ^a^ | ?/low | 0 | 0 | +/moderate | 0 |
| DQOL (Chinese version) | 1 | 0 | 0 | -/high | +/high | ^a^ | 0 | 0 | 0 | 0 | 0 |
| DQOL (Iranian version) | 1 | moderate | 0 | -/high | +/high | ^a^ | -/low | 0 | 0 | 0 | 0 |
| DQOL (Turkish version) | 1 | moderate | 0 | 0 | +/high | ^a^ | ?/low | 0 | 0 | +/high | 0 |
| DQOL (Malay version) | 1 | low | +/low | 0 | +/high | 0 | 0 | 0 | 0 | 0 | 0 |
| IRDQOL | 1 | 0 | 0 | 0 | ?/high | 0 | ?/very low | 0 | 0 | 0 | 0 |
| revised version of DQOL | 1 | low | 0 | +/high | +/high | 0 | 0 | 0 | 0 | 0 | 0 |
| AsianDQOL* | 3 | very low | +/very low | ?/low | +/moderate | ?/very low | ?/very low | 0 | 0 | 0 | 0 |
| AsianDQOL | 1 | low | +/very low | +/high | +/high | ?/low | ?/low | 0 | 0 | 0 | 0 |
| AsianDQOL (Malay version) | 1 | low | +/very low | +/high | +/high | ?/high | ?/low | 0 | 0 | 0 | 0 |
| AsianDQOL (Chinese-mandarin version) | 1 | very low | +/very low | ?/low | +/moderate | ?/very low | ?/very low | 0 | 0 | 0 | 0 |
| DQL-BCI (Polish version) | 1 | 0 | 0 | ?/very low | +/high | ^a^ | ?/low | 0 | 0 | +/high | 0 |
| DQOL-B | 1 | moderate | ?/very low | 0 | 0 | 0 | 0 | 0 | 0 | 0 | 0 |
| QOLID | 1 | moderate | 0 | 0 | -/high | 0 | 0 | 0 | 0 | +/high | 0 |
| J-DQOL | 1 | very low | 0 | ?/very low | +/high | ^a^ | -/high | 0 | 0 | ?/high | 0 |
| QOL questionnaire | 1 | very low | 0 | 0 | +/moderate | 0 | 0 | 0 | 0 | 0 | 0 |
| DMQoL (Persian version) | 1 | 0 | 0 | -/high | +/high | ^a^ | +/high | 0 | 0 | +/high | 0 |
| MENQOL | 1 | 0 | 0 | 0 | +/high | 0 | 0 | 0 | 0 | +/high | 0 |
| Diabetes-39* | 2 | very low | 0 | 0 | -/moderate | ^a^ | 0 | 0 | 0 | +/moderate+/moderate | 0 |
| Diabetes-39 (Arabic version) | 1 | very low | 0 | 0 | +/high | ^a^ | 0 | 0 | 0 | +/high | 0 |
| Diabetes-39 (Brazilian version) | 1 | low | 0 | 0 | -/moderate | ^a^ | 0 | 0 | 0 | +/moderate | 0 |
| ADDQoL-19* | 5 | moderate | ?/very low | ?/very low | +/high | 0 | 0 | 0 | 0 | -/high | 0 |
| ADDQoL-19 | 2 | moderate | ?/very low | -/high | +/high | 0 | 0 | 0 | 0 | +/high | 0 |
| ADDQoL-19 (Chinese version) | 1 | 0 | 0 | 0 | +/high | 0 | 0 | 0 | 0 | -/high | 0 |
| ADDQoL-19 (Italian version) | 1 | 0 | 0 | ?/very low | +/high | 0 | 0 | 0 | 0 | +/high | 0 |
| ADDQoL-19 (Malay version) | 1 | 0 | 0 | -/high | +/high | ^a^ | 0 | 0 | 0 | 0 | 0 |
| ADDQoL* | 5 | very low | 0 | ?/moderate | +/moderate | ^a^ | +/moderate | 0 | 0 | +/moderate | 0 |
| ADDQoL (Chinese version) | 1 | low | 0 | 0 | +/moderate | ^a^ | +/moderate | 0 | 0 | +/moderate | 0 |
| CN-ADDQoL | 1 | moderate | 0 | ?/moderate | +/high | ^a^ | 0 | 0 | 0 | 0 | 0 |
| ADDQoL (Spanish version) | 1 | 0 | 0 | 0 | +/high | ^a^ | 0 | 0 | +/high | 0 | 0 |
| ADDQoL (Turkish version) | 1 | 0 | 0 | 0 | +/high | ?/moderate | 0 | 0 | 0 | 0 | 0 |
| Malay ADDQOL | 1 | very low | 0 | ?/moderate | +/high | ^a^ | +/high | 0 | 0 | 0 | 0 |
| *Elasy et al.* ^b^ | 1 | high | ?/low | 0 | +/high | 0 | 0 | 0 | 0 | +/high | 0 |
| DHP-1 | 1 | 0 | 0 | 0 | ?/high | 0 | 0 | 0 | 0 | +/high | 0 |
| DHP-3D | 1 | 0 | 0 | ?/very low | 0 | 0 | 0 | 0 | 0 | 0 | 0 |
| DHP-5D | 1 | very low | 0 | ?/low | 0 | 0 | 0 | 0 | 0 | 0 | 0 |
| DCP (Chinese version) | 1 | low | 0 | 0 | -/high | ^a^ | 0 | 0 | -/high | +/high | 0 |
| DIMS (Chinese version) | 1 | low | 0 | 0 | -/high | ^a^ | -/very low | 0 | 0 | +/high | 0 |
| **General psychosocial impact** | | | | | | | | | | |  |
| MDQ* | 2 | low | ?/very low | 0 | ?/high | 0 | ?/low | 0 | 0 | +/high | 0 |
| MDQ | 1 | low | ?/very low | 0 | ?/high | 0 | 0 | 0 | 0 | +/high | 0 |
| MDQ (Hindi version) | 1 | low | 0 | 0 | +/high | ^a^ | ?/low | 0 | 0 | 0 | 0 |
| PGWB | 1 | 0 | 0 | 0 | +/high | 0 | 0 | 0 | 0 | +/high | 0 |
| WBQ | 2 | 0 | 0 | 0 | +/moderate | 0 | 0 | 0 | 0 | ?/low | 0 |
| W-BQ28 | 1 | 0 | 0 | +/high | +/high | 0 | 0 | 0 | 0 | +/high | 0 |
| WHO-5 index* | 2 | very low | 0 | ?/moderate | +/high | 0 | 0 | 0 | 0 | +/high | 0 |
| WHO-5 index | 1 | 0 | 0 | +/high | +/high | 0 | 0 | 0 | 0 | 0 | 0 |
| WHO-5 index (Polish version) | 1 | very low | 0 | ?/moderate | +/high | ^a^ | 0 | 0 | 0 | +/high | 0 |
| **Diabetes-related depression** | | | | | | | | | | |  |
| CES-Depression | 4 | 0 | ?/low | +/moderate | +/high | 0 | ?/moderate | 0 | +/high | +/high | 0 |
| Depression in Diabetes Self-Rating Scale | 1 | low | 0 | 0 | ?/moderate | 0 | ?/very low | 0 | 0 | 0 | 0 |
| SCAD | 1 | 0 | 0 | 0 | 0 | 0 | 0 | 0 | -/high | 0 | 0 |
| HADS | 1 | 0 | 0 | 0 | 0 | 0 | 0 | 0 | +/high | 0 | 0 |
| DMI | 1 | 0 | 0 | 0 | 0 | 0 | 0 | 0 | +/high | 0 | 0 |
| EDS | 1 | 0 | 0 | +/high | 0 | 0 | 0 | 0 | 0 | 0 | 0 |
| DCS | 1 | 0 | 0 | ?/very low | +/moderate | 0 | 0 | 0 | 0 | +/moderate | 0 |
| CUDOS-Chinese | 1 | low | +/very low | -/high | +/high | ^a^ | +/high | 0 | +/high | +/high | 0 |
| PHQ-9* | 4 | 0 | +/high | 0 | +/high | 0 | ?/moderate | 0 | +/high | +/high | 0 |
| PHQ-9 | 1 | 0 | 0 | 0 | +/high | 0 | ?/moderate | 0 | +/high | 0 | 0 |
| PHQ-9 | 1 | 0 | +/high | 0 | +/high | 0 | 0 | 0 | +/high | 0 | 0 |
| PHQ-9 (Chichewa version) | 1 | 0 | +/high | 0 | +/high | 0 | 0 | 0 | +/high | 0 | 0 |
| PHQ-9 (Romanian version) | 1 | 0 | 0 | 0 | +/high | 0 | ?/moderate | 0 | 0 | +/high | 0 |
| **Diabetes-related distress** | | | | | | | | | | |  |
| CDDS-17 | 1 | 0 | 0 | ?/moderate | +/high | ^a^ | ?/moderate | 0 | 0 | 0 | 0 |
| DDS Bahasa Indonesia | 1 | low | 0 | ?/very low | +/high | ^a^ | 0 | 0 | 0 | 0 | 0 |
| PAID* | 3 | very low | 0 | -/moderate | +/high | ?/very low | +/high | 0 | +/high | +/high | 0 |
| PAID | 1 | 0 | 0 | +/high | +/high | 0 | 0 | 0 | 0 | +/high | 0 |
| PAID (Spanish version) | 1 | 0 | 0 | -/moderate | 0 | ?/very low | 0 | 0 | 0 | 0 | 0 |
| PAID (Greek version) | 1 | very low | 0 | ?/moderate | +/high | ^a^ | +/high | 0 | +/high | 0 | 0 |
| MY-PAID-20 | 1 | 0 | 0 | -/high | +/high | 0 | 0 | 0 | +/high | 0 | 0 |
| B-PAID | 1 | 0 | 0 | 0 | -/high | ^a^ | 0 | 0 | 0 | +/high | 0 |
| PAID-K | 1 | low | 0 | 0 | ?/high | ^a^ | 0 | 0 | 0 | 0 | 0 |
| K-PAID | 1 | 0 | 0 | -/high | -/high | ^a^ | -/high | 0 | 0 | -/high | 0 |
| K-PAID-5 | 1 | 0 | 0 | +/high | +/high | ^a^ | +/high | 0 | 0 | +/high | 0 |
| Turkish PAID | 1 | 0 | 0 | 0 | +/high | ^a^ | 0 | 0 | 0 | +/high | 0 |
| SG-PAID-C | 1 | 0 | 0 | ?/moderate | -/high | ^a^ | 0 | 0 | 0 | +/high | 0 |
| IR-PAID-20 | 1 | 0 | 0 | ?/low | +/high | ^a^ | ?/very low | 0 | ?/very low | 0 | 0 |
| **Self-efficacy** | | | | | | | | | | |  |
| SE-Type 2 | 1 | 0 | ?/moderate | 0 | -/moderate | 0 | +/moderate | 0 | 0 | ?/low | 0 |
| DMSES* | 5 | very low | ?/low | ?/very low | +/moderate | 0 | +/moderate | 0 | +/moderate | +/moderate | 0 |
| DMSES | 1 | 0 | +/moderate | 0 | +/moderate | 0 | +/moderate | 0 | +/moderate | +/moderate | 0 |
| K-DMSES | 1 | very low | ?/moderate | -/high | +/high | ^a^ | +/high | 0 | 0 | +/high | 0 |
| GR-DMSES | 1 | moderate | +/high | ?/moderate | +/high | ^a^ | +/high | 0 | 0 | 0 | 0 |
| DMSES (Brazilian version) | 1 | moderate | 0 | -/high | -/high | ^a^ | 0 | 0 | 0 | +/high | 0 |
| IT-DMSES | 1 | low | ?/low | ?/very low | +/high | ^a^ | 0 | 0 | 0 | +/high | 0 |
| DSEQ (Thai version) | 1 | very low | +/very low | +/low | 0 | ^a^ | 0 | 0 | 0 | 0 | 0 |
| CDMSS-11 | 1 | low | ?/very low | ?/moderate | +/high | ^a^ | +/high | 0 | 0 | 0 | 0 |
| DSCAS | 1 | very low | +/very low | 0 | 0 | 0 | 0 | 0 | 0 | 0 | 0 |
| DSES | 1 | very low | +/very low | 0 | 0 | 0 | 0 | 0 | 0 | 0 | 0 |
| K-DSES | 1 | moderate | 0 | ?/high | +/high | ^a^ | -/high | 0 | 0 | 0 | 0 |
| Situational Self-Efficacy Scales (Spanish version) | 1 | very low | +/very low | 0 | ?/low | ^a^ | ?/very low | 0 | 0 | 0 | 0 |
| ESS | 1 | 0 | 0 | -/high | +/high | ^a^ | 0 | 0 | 0 | +/high | 0 |
| Self-Efficacy for Exercise 1 (Spanish version) | 1 | very low | +/very low | 0 | ?/low | ^a^ | ?/very low | 0 | 0 | 0 | 0 |
| Self-Efficacy for Exercise 2 (Spanish version) | 1 | very low | +/very low | 0 | ?/low | ^a^ | ?/very low | 0 | 0 | 0 | 0 |
| PTES | 1 | very low | ?/low | +/high | +/high | ^a^ | 0 | 0 | 0 | 0 | 0 |
| **Self-management** | | | | | | | | | | |  |
| SDSCA* | 6 | very low | +/very low | -/very low | ?/very low | 0 | ?/low | 0 | 0 | 0 | ?/very low |
| SDSCA | 1 | 0 | 0 | 0 | ?/very low | 0 | ?/moderate | 0 | 0 | 0 | ?/very low |
| SDSCA (Turkish version) | 1 | very low | +/moderate | -/very low | -/high | ^a^ | 0 | 0 | 0 | 0 | 0 |
| SDSCA-G | 1 | low | 0 | +/high | -/high | ^a^ | 0 | 0 | 0 | +/high | 0 |
| SDSCA (Moroccan version) | 1 | very low | 0 | ?/low | -/moderate | ^a^ | ?/low | 0 | 0 | 0 | 0 |
| SDSCA-Ar | 1 | very low | +/very low | ?/moderate | -/high | ^a^ | 0 | 0 | 0 | +/high | 0 |
| SDSCA-K | 1 | low | ?/low | -/high | -/high | ^a^ | 0 | 0 | 0 | 0 | 0 |
| INAAP-DM2 | 1 | very low | +/low | 0 | 0 | 0 | 0 | 0 | 0 | 0 | 0 |
| SCI-R | 1 | 0 | 0 | +/high | +/high | 0 | 0 | 0 | 0 | +/high | 0 |
| DSSCI | 1 | low | 0 | 0 | ?/moderate | ^a^ | ?/very low | 0 | 0 | +/moderate | 0 |
| SUGAAR | 1 | 0 | +/high | 0 | 0 | 0 | 0 | 0 | 0 | +/high | 0 |
| D-SMART® | 1 | low | 0 | 0 | ?/high | ^a^ | ?/moderate | 0 | 0 | 0 | 0 |
| ES-SMBPA-2D | 1 | moderate | ?/moderate | 0 | -/high | ^a^ | Very good/- | 0 | 0 | +/high | 0 |
| DSMS | 1 | very low | +/very low | 0 | 0 | 0 | 0 | 0 | 0 | 0 | 0 |
| DSMQ* | 2 | moderate | +/moderate | 0 | +/high | ^a^ | 0 | 0 | 0 | 0 | 0 |
| DSMQ (Thai version) | 1 | moderate | +/moderate | 0 | +/high | ^a^ | 0 | 0 | 0 | 0 | 0 |
| DSMQ (Urdu version) | 1 | low | 0 | +/high | +/high | ^a^ | 0 | 0 | 0 | +/high | 0 |
| V-DSMI | 1 | very low | 0 | -/high | +/high | ^a^ | 0 | 0 | 0 | 0 | 0 |
| DSMI-20 | 1 | low | +/high | ?/moderate | +/high | ^a^ | ?/moderate | 0 | 0 | +/high | 0 |
| DSMB-O | 1 | very low | +/moderate | 0 | 0 | ^a^ | -/high | 0 | 0 | 0 | 0 |
| SMP-T2D | 1 | moderate | 0 | 0 | +/high | 0 | ?/moderate | 0 | 0 | +/high | +/high |
| PAM13 | 1 | high | 0 | +/high | 0 | +/high | 0 | 0 | 0 | 0 | 0 |
| *Chernyak et al.* ^b^ | 1 | high | +/high | 0 | 0 | 0 | 0 | 0 | 0 | +/high | 0 |
| CIRS (Thai version) | 1 | low | +/low | 0 | ?/moderate | ^a^ | 0 | 0 | 0 | 0 | 0 |
| **Impact of empowerment tools** | | | | | | | | | | |  |
| IR-DES-28 | 1 | 0 | 0 | ?/very low | +/high | ^a^ | ?/moderate | 0 | 0 | +/high | 0 |
| *Hara et al.* ^b^ | 1 | 0 | 0 | 0 | -/high | 0 | -/moderate | 0 | 0 | +/high | 0 |
| DES-M | 1 | moderate | 0 | ?/high | -/high | ^a^ | 0 | 0 | 0 | -/high | 0 |
| DES-SF* | 2 | moderate | +/high | -/high | -/high | ^a^ | -/high | 0 | 0 | 0 | 0 |
| DES-SF (Brazilian Portuguese version) | 1 | moderate | +/high | 0 | -/high | ^a^ | -/high | 0 | 0 | 0 | 0 |
| DES-SF (Portuguese version) | 1 | 0 | 0 | -/high | +/high | ^a^ | -/high | 0 | 0 | 0 | 0 |
| **Health-promoting lifestyle behaviours** | | | | | | | | | | |  |
| T2DHPS* | 2 | very low | +/high | ?/very low | -/high | ^a^ | ?/moderate | 0 | 0 | +/high | 0 |
| T2DHPS (Persian version) | 1 | very low | 0 | ?/very low | +/high | ^a^ | ?/moderate | 0 | 0 | +/high | 0 |
| T2DHPS (Turkish version) | 1 | low | +/high | -/high | -/high | ^a^ | 0 | 0 | 0 | 0 | 0 |
| DHPSC (Chinese version) | 1 | 0 | +/high | -/high | +/high | ^a^ | +/high | 0 | 0 | 0 | 0 |
| PDQ-11 | 1 | 0 | ?/moderate | -/high | 0 | 0 | 0 | 0 | 0 | 0 |  |
| C-PDQ | 1 | low | +/high | ?/moderate | +/high | ^a^ | +/high | 0 | +/high | +/high | 0 |
| **Health beliefs** | | | | | | | | | | |  |
| Health Belief Measures | 1 | very low | ?/low | 0 | +/high | 0 | 0 | 0 | 0 | +/high | 0 |
| Given Health Belief Instrument (Spanish version) | 1 | high | ?/low | 0 | -/high | ^a^ | 0 | 0 | 0 | 0 | 0 |
| Health belief model scale (Turkish version) | 1 | 0 | +/moderate | 0 | +/high | ^a^ | +/high | 0 | 0 | +/high | 0 |
| Diabetes Health Belief Measure | 1 | 0 | 0 | 0 | -/high | 0 | ?/very low | 0 | 0 | 0 | 0 |
| **Knowledge/competence** | | | | | | | | | | |  |
| Diabetes Questionnaire* | 2 | 0 | 0 | ?/very low | 0 | 0 | 0 | 0 | 0 | 0 | 0 |
| Diabetes Questionnaire | 1 | 0 | 0 | ?/very low | 0 | 0 | 0 | 0 | 0 | 0 | 0 |
| Diabetes Questionnaire (Spanish version) | 1 | 0 | 0 | ?/very low | 0 | ^a^ | 0 | 0 | 0 | 0 | 0 |
| Diabetes Knowledge Questionnaire (Spanish version) | 1 | very low | +/very low | 0 | ?/low | ^a^ | ?/very low | 0 | 0 | 0 | 0 |
| DKQ-24 | 1 | 0 | 0 | 0 | +/high | 0 | ?/very low | 0 | 0 | 0 | 0 |
| DMKT | 1 | low | +/high | 0 | +/high | ^a^ | 0 | 0 | 0 | +/high | 0 |
| PCSD-P | 1 | low | +/high | -/high | +/high | ^a^ | +/high | 0 | 0 | 0 | 0 |
| *Miller et al.* ^b^ | 1 | very low | +/very low | 0 | ?/low | 0 | ?/very low | 0 | 0 | +/low | 0 |
| *Miller & Edwards*^b^ | 1 | low | +/moderate | 0 | 0 | 0 | 0 | 0 | +/high | 0 | +/high |
| PDDC | 1 | very low | 0 | 0 | ?/high | 0 | ?/moderate | 0 | 0 | +/high | 0 |
| DRNK | 1 | very low | +/low | 0 | -/low | 0 | +/low | 0 | 0 | +/low | 0 |
| FCCHL (Norwegian version) | 1 | low | 0 | -/high | 0 | ^a^ | 0 | 0 | 0 | 0 | 0 |
| KHLS-DM | 1 | low | +/moderate | +/high | +/high | ^a^ | ?/very low | 0 | 0 | 0 | 0 |
| HLS-K | 1 | very low | 0 | -/high | +/high | ^a^ | 0 | 0 | 0 | +/high | 0 |
| HLS/SNS | 1 | 0 | 0 | -/high | +/high | 0 | 0 | 0 | -/high | 0 | 0 |
| *Ashok et al. 1*^b^ | 1 | 0 | 0 | ?/very low | +/low | 0 | +/low | 0 | 0 | +/low | 0 |
| *Ashok et al. 2*^b^ | 1 | 0 | 0 | ?/very low | +/low | 0 | +/low | 0 | 0 | +/low | 0 |
| HLS-EU-Q47 | 1 | low | 0 | -/high | +/high | 0 | 0 | 0 | 0 | 0 | 0 |
| **Treatment experience** | | | | | | | | | | |  |
| DTSQ | 2 | 0 | 0 | +/moderate | +/moderate | 0 | 0 | 0 | 0 | 0 | 0 |
| DTSQ | 1 | 0 | 0 | 0 | +/moderate | 0 | 0 | 0 | 0 | 0 | 0 |
| DTSQ (Greek version) | 1 | 0 | 0 | +/moderate | +/high | ^a^ | 0 | 0 | 0 | 0 | 0 |
| DiabMedSat | 1 | 0 | 0 | 0 | 0 | 0 | 0 | 0 | 0 | 0 | +/high |
| DTBQ | 1 | 0 | 0 | ?/very low | +/high | 0 | +/high | 0 | 0 | 0 | 0 |
| ITEQ | 1 | high | 0 | 0 | -/moderate | 0 | 0 | 0 | 0 | +/high | 0 |
| IITQ | 1 | 0 | 0 | 0 | +/high | 0 | -/high | 0 | 0 | 0 | 0 |
| ITAS | 1 | very low | 0 | 0 | -/high | 0 | 0 | 0 | 0 | +/high | 0 |
| C-ITAS-HK | 1 | moderate | +/high | ?/moderate | -/high | ^a^ | ?/moderate | 0 | 0 | +/high | 0 |
| BITQ (Turkish version) | 1 | low | 0 | -/high | +/high | ^a^ | ?/moderate | 0 | 0 | 0 | 0 |
| Ch-ASIQ | 1 | low | +/high | ?/moderate | -/high | ^a^ | 0 | 0 | 0 | 0 | 0 |
| MIAS | 1 | 0 | 0 | 0 | -/high | 0 | 0 | 0 | +/high | +/high | 0 |
| IMDSES (Brazilian version) | 1 | 0 | 0 | 0 | +/high | ^a^ | 0 | 0 | -/high | +/high | 0 |
| ITSQ | 1 | very low | 0 | 0 | +/high | 0 | -/moderate | 0 | 0 | +/high | 0 |
| OHA-Q | 1 | 0 | 0 | ?/moderate | +/high | 0 | 0 | 0 | 0 | 0 | 0 |
| DMSRQ | 1 | 0 | 0 | ?/low | +/high | ^a^ | ?/moderate | 0 | 0 | +/high | 0 |
| **Treatment compliance** | | | | | | | | | | |  |
| *Demirtas et al*. ^b^ | 1 | moderate | ?/low | ?/moderate | +/high | 0 | ?/very low | 0 | +/high | 0 | 0 |
| MMAS * | 5 | low | 0 | ?/moderate | -/high | ^a^ | +/high | 0 | ?/high | -/high | 0 |
| MMAS (Thai version) | 1 | low | 0 | ?/high | -/high | ^a^ | +/high | 0 | 0 | 0 | 0 |
| MMAS-8 (Korean version) | 1 | moderate | 0 | -/high | -/high | ^a^ | +/high | 0 | ?/high | 0 | 0 |
| MMAS-8 (Chinese version) | 1 | 0 | 0 | ?/moderate | -/high | ^a^ | +/high | 0 | 0 | +/high | 0 |
| MMAS-8 (French version) | 1 | 0 | 0 | 0 | 0 | ^a^ | 0 | 0 | 0 | -/high | 0 |
| MMAS-8 (French version) | 1 | 0 | 0 | ?/moderate | -/high | ^a^ | 0 | 0 | 0 | 0 | 0 |
| modiﬁed 4-item Morisky–Green–Levine Medication Adherence Scale | 1 | 0 | 0 | ?/very low | -/high | 0 | 0 | 0 | 0 | 0 | 0 |
| MGLS (Indonesian version) | 1 | low | 0 | 0 | -/high | ^a^ | +/high | 0 | +/high | +/high | 0 |
| Medical Prescription Knowledge questionnaire | 1 | low | +/high | 0 | ?/moderate | 0 | 0 | 0 | +/high | 0 | 0 |
| Attitude Scale | 1 | low | +/high | 0 | ?/moderate | 0 | 0 | 0 | +/high | 0 | 0 |
| BMQ-f | 1 | very low | 0 | -/high | +/high | ^a^ | 0 | 0 | 0 | 0 | 0 |
| MALMAS | 1 | 0 | 0 | 0 | -/high | 0 | ?/moderate | 0 | 0 | +/high | 0 |
| MAT OADs | 1 | 0 | +/very low | 0 | +/low | 0 | 0 | 0 | +/low | 0 | 0 |
| MAT insulin | 1 | 0 | +/very low | 0 | -/low | 0 | 0 | 0 | +/low | 0 | 0 |
| ARMS-K | 1 | very low | 0 | ?/moderate | +/high | ^a^ | 0 | 0 | 0 | +/high | 0 |
| Diabetes Medication System Rating Questionnaire Short-Form | 1 | very low | 0 | 0 | +/high | 0 | ?/very low | 0 | +/high | +/high | 0 |
| SR-4 (French version) | 1 | 0 | 0 | 0 | 0 | ^a^ | 0 | 0 | 0 | -/high | 0 |
| *Zongo et al. 1*^b^ | 1 | 0 | 0 | 0 | 0 | ^a^ | 0 | 0 | 0 | -/high | 0 |
| *Zongo et al. 2*^b^ | 1 | 0 | 0 | 0 | 0 | ^a^ | 0 | 0 | 0 | -/high | 0 |
| **Symptoms** | | | | | | | | | | |  |
| HPQ* | 2 | 0 | 0 | -/high | +/high | ^a^ | 0 | 0 | 0 | +/high | 0 |
| HPQ (Cyprus version) | 1 | 0 | 0 | +/high | +/high | ^a^ | 0 | 0 | 0 | +/high | 0 |
| HPQ | 1 | 0 | 0 | -/high | +/high | 0 | 0 | 0 | 0 | +/high | 0 |
| CHI (Filipino version) | 1 | low | 0 | 0 | -/high | ^a^ | 0 | 0 | -/moderate | 0 | 0 |
| FH-15 (Chinese version) | 1 | 0 | +/moderate | -/high | +/high | ^a^ | ?/moderate | 0 | 0 | 0 | 0 |
| K-DSC-R | 1 | 0 | 0 | +/high | -/high | ^a^ | 0 | 0 | 0 | +/high | 0 |
| DSC-R | 1 | 0 | 0 | +/high | -/high | 0 | 0 | 0 | 0 | 0 | ?/very low |
| *Naegeli et al.* ^b^ | 1 | 0 | 0 | +/high | +/high | 0 | -/high | 0 | 0 | +/high | 0 |
| FACIT-Fatigue Scale | 1 | low | +/moderate | ?/high | +/high | ^a^ | ?/very low | 0 | 0 | 0 | 0 |
| **Nutrition and physical activity** | | | | | | | | | | |  |
| Barriers to Fat Reduction Scale (Spanish version) | 1 | very low | +/very low | 0 | -/low | ^a^ | ?/very low | 0 | 0 | 0 | 0 |
| Barriers to Exercise Checklist (Spanish version) | 1 | very low | +/very low | 0 | ?/low | ^a^ | ?/very low | 0 | 0 | 0 | 0 |
| Food Habits Questionnaire (Spanish version) | 1 | very low | +/very low | 0 | ?/low | ^a^ | ?/very low | 0 | 0 | 0 | 0 |
| DDRQOL | 1 | very low | 0 | ?/high | +/high | 0 | -/high | 0 | 0 | +/high | 0 |
| DDRQOL-R | 1 | moderate | 0 | ?/moderate | +/high | 0 | -/high | 0 | +/high | 0 | 0 |
| *Sato et al.* ^b^ | 1 | moderate | 0 | 0 | +/high | 0 | -/high | 0 | +/high | 0 | 0 |
| IW-SP | 1 | low | 0 | ?/very low | +/high | 0 | +/high | 0 | 0 | +/high | 0 |
| Motiva.Diaf-DM2 questionnaire | 1 | 0 | 0 | -/high | +/high | 0 | ?/moderate | 0 | 0 | 0 | 0 |
| HAPA-based PA inventory | 1 | 0 | +/high | +/moderate | -/high | 0 | +/high | 0 | 0 | 0 | 0 |
| **Sleep** | | | | | | | | | | |  |
| STOP-Bang questionnaire | 1 | 0 | 0 | 0 | 0 | 0 | 0 | 0 | +/high | 0 | 0 |
| PROMIS - Sleep Disturbance instrument | 1 | 0 | 0 | 0 | 0 | 0 | 0 | 0 | 0 | -/high | ?/high |
| PROMIS - Sleep Related Impairment instrument | 1 | 0 | 0 | 0 | 0 | 0 | 0 | 0 | 0 | -/high | ?/high |
| **Support** | | | | | | | | | | |  |
| RSSM-Farsi | 1 | low | 0 | -/moderate | -/high | ^a^ | +/high | 0 | 0 | 0 | 0 |
| DFBC (Japanese version) | 1 | low | 0 | -/moderate | +/high | ^a^ | +/high | 0 | 0 | 0 | 0 |
| FSS-AA T2DM | 1 | low | 0 | ?/moderate | +/high | 0 | ?/moderate | 0 | 0 | +/high | 0 |
| HCCQ-P | 1 | low | +/high | -/high | +/high | ^a^ | +/high | 0 | 0 | 0 | 0 |
| The Diabetes Family Support and Conflict Scale (Turkish version) | 1 | low | +/moderate | ?/moderate | +/high | 0 | 0 | 0 | 0 | 0 | 0 |
| **Attitude/coping with diabetes** | | | | | | | | | | |  |
| SSS-J | 1 | low | ?/low | -/high | +/high | ^a^ | ?/moderate | 0 | 0 | +/high | 0 |
| DSAS-2 | 1 | very low | 0 | ?/very low | +/high | 0 | 0 | 0 | 0 | +/high | 0 |
| Relationship Consciousness of Japanese Patients with Type 2 Diabetes Mellitus Scale | 1 | low | +/moderate | ?/moderate | +/high | 0 | 0 | 0 | 0 | +/high | 0 |
| ADS* | 2 | very low | 0 | ?/very low | -/high | ^a^ | ?/very low | 0 | 0 | Very good/+ | 0 |
| ADS (Japanese version) | 1 | very low | 0 | 0 | -/high | ^a^ | ?/very low | 0 | 0 | 0 | 0 |
| ADS (Korean version) | 1 | very low | 0 | ?/very low | +/high | ^a^ | 0 | 0 | 0 | Very good/+ | 0 |
| DAAS | 1 | low | +/moderate | ?/moderate | +/high | 0 | 0 | 0 | 0 | 0 | 0 |
| IR-DAS-3 | 1 | 0 | 0 | 0 | ?/high | ^a^ | ?/very low | 0 | 0 | 0 | 0 |
| GCQ | 1 | 0 | 0 | ?/moderate | +/high | 0 | 0 | 0 | 0 | Very good/+ | 0 |
| DIAB-Q | 1 | high | 0 | +/high | -/high | 0 | -/high | 0 | 0 | Very good/+ | 0 |
| S-BRCS | 1 | very low | 0 | ?/moderate | +/high | 0 | ?/low | 0 | 0 | 0 | 0 |
| **Obstacles and problem-solving** | | | | | | | | | | |  |
| DPMD | 1 | very low | +/very low | 0 | +/low | 0 | ?/very low | 0 | 0 | -/low | 0 |
| DOQ* | 2 | low | +/moderate | 0 | -/high | 0 | 0 | 0 | +/high | +/high | 0 |
| DOQ | 1 | low | +/moderate | 0 | +/high | 0 | 0 | 0 | +/high | +/high | 0 |
| DOQ (Dutch version) | 1 | low | 0 | 0 | -/high | ^a^ | 0 | 0 | 0 | +/high | 0 |
| DOQ-30 | 1 | 0 | 0 | ?/moderate | -/high | ^a^ | 0 | 0 | 0 | 0 | 0 |
| DPSS | 1 | 0 | 0 | ?/very low | +/low | 0 | 0 | 0 | 0 | +/low | 0 |
| **Health perception** | | | | | | | | | | |  |
| IPQ-R | 1 | 0 | 0 | -/high | -/high | 0 | -/very low | 0 | 0 | 0 | 0 |
| CHES-Q | 1 | very low | 0 | -/high | 0 | 0 | ?/moderate | 0 | 0 | +/high | 0 |
| MBIPQ | 1 | 0 | 0 | 0 | 0 | ?/very low | -/high | 0 | 0 | +/high | 0 |
| DFS | 1 | low | 0 | 0 | +/high | 0 | 0 | 0 | 0 | +/high | 0 |
|  |  |  |  |  |  |  |  |  |  |  |  |

0: Measurement property was not assessed by the study

*Pooled results of different language versions of the same PROM

^a^ Only translation was done. Cross-cultural validation was not the aim of the study

^b^PROMs without proper names are labelled based on the name of the original author who developed the instrument

+, positive; -, negative; ±, inconsistent; ?, indeterminate

**Abbreviations**: WHOQOL-100: World Health Organisation quality of life questionnaire; WHOQOL-BREF/ WHOQOL-BREF 26: abbreviated World Health Organization Quality of Life; WHOQOL-BREF 26; HSQ 2.0: Health Status Questionnaire 2.0; HUI2: Health Utilities Index Mark 2; HUI3: Health Utilities Index Mark 3; EQ-5D: EuroQol 5-Dimension; EQ-5D-5L: EQ-5D with 5 level scale; EQ-5D-3L: EQ-5D with 3 level scale; PACIC: Patient Assessment of Chronic Illness Care; SF-36: 36-Item Short Form Survey; SF-12v2: Short Form-12 Health Survey version 2; MDQ: Multidimensional Diabetes Questionnaire; PDQ-11: Personal Diabetes Questionnaire; C-PDQ: Chinese version of PDQ; PRO-DM-Thai: instrument for patient-reported outcomes in Thai patients with type 2 diabetes mellitus; DQOL: Diabetes Quality-of-Life Measure; IRDQOL: Iranian diabetes quality of life; AsianDQOL: Asian Diabetes Quality of Life; DQL-BCI: Diabetes Quality of Life - Brief Clinical Inventory; DQOL-B: Diabetes Quality of Life Brief Clinical Inventory; QOLID: Quality of Life Instrument for Indian Diabetes patients; J-DQOL: Japanese version of the Diabetes Quality-Of-Life; QOL: quality of life; DMQoL: Diabetes-Mellitus Specific Quality of Life; MENQOL: Menopause-specific Quality of Life; ADDQoL-19: 19-item Audit of Diabetes-Dependent Quality of Life; CN-ADDQoL: Adaptation of the ADDQoL questionnaire to people with diabetes in China; DHP-1: Diabetes Health Profile; DHP-3D: Diabetes Health Proﬁle–3 Dimension; DHP-5D: Diabetes Health Proﬁle–5 Dimension; DCP: Diabetes Care Profile; DIMS: diabetes impact measurement scales; PGWB: Psychological General Well-Being Questionnaire; WBQ: Well-being Questionnaire; W-BQ28: 28-item Well-Being Questionnaire; CES-Depression: Center for Epidemiological Studies Depression scale; SCAD: Silverstone Concise Assessment for Depression; HADS: Hospital Anxiety and Depression Scale; DMI: Depression in the Medically Ill Questionnaire; EDS: Edinburgh Depression Scale; DCS: Depressive Cognition Scale; CUDOS-Chinese: Mandarin Chinese Version of the Clinically Useful Depression Outcome Scale; CDDS-17: Chinese version of the Diabetes Distress Scale; DDS Bahasa Indonesia: Indonesian Diabetes Distress Scale; WHO-5 well-being index: 5-item World Health Organization well-being index; PAID: Problem Areas in Diabetes; MY-PAID-20: Malaysian version of PAID; B-PAID: Brazilian version of PAID; PAID-K: Korean version of PAID; short form Korean version of PAID; IR-PAID-20: Iranian version of PAID; PHQ-9: Patient Health Questionnaire-9; SE-Type 2: self-efficacy scale for patients with type 2 diabetes mellitus; DMSES: diabetes management self-efficacy scale; K-DMSES: Korean version of DMSES; GR-DMSES Greek version of DMSES; IT-DMSES: Italian version of DMSES; CDMSS-11: Chinese version of Diabetes Medication Self-efficacy Scale; DSCAS: Diabetes Self-Care Agency Scale; SDSCA: Summary of diabetes self-care activities measure; SDSCA-G: German version of SDSCA; SDSCA-Ar: Arabic version of SDSCA; SDSCA-K: Korean version of SDSCA; INAAP-DM2: Self-care Assessment Instrument for patients with type 2 diabetes mellitus; SCI-R: Self-Care Inventory-Revised; DSSCI: Diabetes Symptom Self-Care Inventory; SUGAAR: Self-Care Utility Geriatric African-American Rating; DSES: Diabetes Self-efficacy Scale; K-DSES: Korean version of DSES; ESS: Exercise Self-efficacy Scale; D-SMART®: Diabetes Self-management Assessment Report Tool; ES-SMBPA-2D: evaluation scale for self-management behavior related to physical activity of type 2 diabetic patients; DSMS: Diabetes Self-Management Scale; DSMQ: Diabetes Self-management Questionnaire; V-DSMI: Vietnamese version of diabetes self-management instrument; DSMI-20: Diabetes Self-Management Instrument Short Form; DSMB-O: Diabetes Self-Management Behavior for Older Koreans; SMP-T2D: self-management profile for type 2 diabetes; IR-DES-28: Iranian version of Diabetes Empowerment Scale; DES-M: diabetes empowerment scale; DES-SF: Diabetes Empowerment Scale - Short Form; T2DHPS: Type 2 Diabetes and Health Promotion Scale; DHPSC: diabetes health promotion self-care scale; PAM13: Patient Activation Measure; DKQ-24: Diabetes Knowledge Questionnaire; DMKT: Diabetes Mellitus Knowledge Test; PCSD-P: Persian Version of Perceived Competence Scale for Diabetes; PDDC: measure of perceived diabetes and dietary competence; DRNK: diabetes-related nutrition knowledge questionnaire; FCCHL: Functional, Communicative, and Critical Health Literacy Scale; KHLS-DM: Korean Health Literacy Scale for Diabetes Mellitus; HLS-K: Health Literacy Scale; HLS/SNS: Health Literacy Scale/Subjective Numeracy Scale; HLS-EU-Q47: European Health Literacy Survey Questionnaire; DTSQ: Diabetes Treatment Satisfaction Questionnaire; DiabMedSat: Diabetes Medication Satisfaction measure; PTES: Perceived Therapeutic Efficacy Scale; DTBQ: Diabetic Treatment Burden Questionnaire; ITEQ: insulin treatment experience questionnaire; IITQ: inhaled insulin treatment questionnaire; ITAS: insulin treatment appraisal scale; C-ITAS-HKeq; BITQ: barriers to insulin treatment questionnaire; Ch-ASIQ: Chinese Attitudes to Starting Insulin Questionnaire; MIAS: Morisky Medication Adherence Scale adapted to specify insulin adherence; IMDSES: Insulin Management Diabetes Self-Efficacy Scale; ITSQ: Insulin Treatment Satisfaction Questionnaire; MMAS/MMAS-8: 8-item Morisky Medication Adherence Scale; MGLS: 4 items Morisky Green Levine Adherence Scale; OHA-Q: Oral Hypoglycemic Agent Questionnaire; DMSRQ: Diabetes Medication System Rating Questionnaire; BMQ-f: French version of the Beliefs about Medicines Questionnaire; MALMAS: Malaysian Medication Adherence Scale; MAT OADs: Measurement of Adherence to Drug Therapy in Diabetes Mellitus – Oral Antidiabetics; MAT insulin: Measurement of Adherence to Drug Therapy in Diabetes Mellitus – Insulin Therapy; ARMS-K: Korean version of the Adherence to Refills and Medications Scale; SR-4: self-report with 4 items; HPQ: Hypoglycemia Perspectives Questionnaire; CHI: Clarke Hypoglycemia Index; FH-15: Chinese version of the new fear of hypoglycemia scale; K-DSC-R: Korean version of Diabetes Symptom Checklist-Revised; DSC-R: Diabetes Symptom Checklist-Revised; DDRQOL: diabetes diet-related quality-of-life scale; DDRQOL-R: revised and short form versions of diabetes diet-related quality of life scale; IW-SP: Impact of Weight on Self-Perceptions Questionnaire; HAPA-based PA inventory: health action process approach (HAPA)-based PA inventory; RSSM-Farsi: Iranian version of Resources and Support for Chronic illness Self-management scale; DFBC: Diabetes Family Behavior Checklist; FSS-AA T2DM: Family Support Scale Adapted for African American Women with Type 2 Diabetes Mellitus; HCCQ-P: Persian health care climate questionnaire; SSS-J: Japanese version of the self-stigma scale; DSAS-2: Type 2 Diabetes Stigma Assessment Scale; ADS: Appraisal of Diabetes Scale; DAAS: Diabetes Adjustment Assessment Scale; IR-DAS-3: Iranian Diabetes Attitude Scale; GCQ: General Coping Questionnaire; DIAB-Q: 17-item Diabetes Intention, Attitude, and Behavior Questionnaire; S-BRCS: Spanish Brief Religious Coping Scale; DPMD: diabetes-specific measure of patient desire to participate in medical decision making; DOQ: Diabetes Obstacles Questionnaire; DOQ-30: short version of the diabetes obstacles questionnaire; DPSS: Diabetes Problem-Solving Scale; CIRS: Chronic Illness Resources Survey; IPQ-R: Revised Illness Perception Questionnaire; CHES-Q: 14-item Current Health Satisfaction Questionnaire; MBIPQ: Malay version of the brief illness perception questionnaire; DFS: 12-item diabetes fatalism scale

**Supplementary Table 7**│ **Evidence synthesis of measurement properties for each PROM (based on COSMIN Risk of Bias checklist) – Summarized table**

|  | | **Overall level of evidence for measurement properties** | | | | | | | | | |
| --- | --- | --- | --- | --- | --- | --- | --- | --- | --- | --- | --- |
| **PROM** | **No. of studies** | **PROM development** | **Content validity** | **Structural validity** | **Internal consistency** | **Cross-cultural validity\ Measurement invariance** | **Reliability** | **Measurement error** | **Criterion validity** | **Hypotheses testing for construct validity** | **Responsiveness** |
| **General impact on quality of life** | | | | | | | | | | |  |
| HSQ 2.0 (Spanish version) | 1 | very low | +/very low | 0 | -/low | ^a^ | ?/very low | 0 | 0 | 0 | 0 |
| PACIC | 3 | moderate | +/high | ?/low | +/high | 0 | +/high | 0 | ?/very low | 0 | 0 |
| **Diabetes-specific impact on quality of life** | | | | | | | | | | |  |
| PRO-DM-Thai | 1 | high | +/high | +/high | +/high | ^a^ | 0 | 0 | 0 | +/high | 0 |
| DQOL | 5 | low | +/low | -/high | ?/moderate | 0 | -/low | 0 | 0 | +/moderate | 0 |
| AsianDQOL | 3 | Very low | +/very low | ?/low | +/moderate | ?/very low | ?/very low | 0 | 0 | 0 | 0 |
| **General psychosocial impact** | | | | | | | | | | |  |
| **Diabetes-related depression** | | | | | | | | | | |  |
| CUDOS-Chinese | 1 | low | +/very low | -/high | +/high | ^a^ | +/high | 0 | +/high | +/high | 0 |
| PHQ-9 | 4 | 0 | +/high | 0 | +/high | 0 | ?/moderate | 0 | +/high | +/high | 0 |
| **Self-efficacy** | | | | | | | | | | |  |
| DMSES | 5 | very low | ?/low | ?/very low | +/moderate | 0 | +/moderate | 0 | +/moderate | +/moderate | 0 |
| Situational Self-Efficacy Scales (Spanish version) | 1 | very low | +/very low | 0 | ?/low | ^a^ | ?/very low | 0 | 0 | 0 | 0 |
| Self-Efficacy for Exercise 1 (Spanish version) | 1 | very low | +/very low | 0 | ?/low | ^a^ | ?/very low | 0 | 0 | 0 | 0 |
| Self-Efficacy for Exercise 2 (Spanish version) | 1 | very low | +/very low | 0 | ?/low | ^a^ | ?/very low | 0 | 0 | 0 | 0 |
| **Self-management** | | | | | | | | | | |  |
| DSMQ | 2 | moderate | +/moderate | 0 | +/high | ^a^ | 0 | 0 | 0 | 0 | 0 |
| DSMI-20 | 1 | low | +/high | ?/moderate | +/high | ^a^ | ?/moderate | 0 | 0 | +/high | 0 |
| CIRS (Thai version) | 1 | low | +/low | 0 | ?/moderate | ^a^ | 0 | 0 | 0 | 0 | 0 |
| **Impact of empowerment tools** | | | | | | | | | | |  |
| DES-SF | 2 | moderate | +/high | -/high | -/high | ^a^ | -/high | 0 | 0 | 0 | 0 |
| **Health-promoting lifestyle behaviours** | | | | | | | | | | |  |
| T2DHPS* | 2 | very low | +/high | ?/very low | -/high | ^a^ | ?/moderate | 0 | 0 | +/high | 0 |
| DHPSC (Chinese version) | 1 | 0 | +/high | -/high | +/high | ^a^ | +/high | 0 | 0 | 0 | 0 |
| C-PDQ | 1 | low | +/high | ?/moderate | +/high | ^a^ | +/high | 0 | +/high | +/high | 0 |
| **Health beliefs** | | | | | | | | | | |  |
| Health belief model scale (Turkish version) | 1 | 0 | +/moderate | 0 | +/high | ^a^ | +/high | 0 | 0 | +/high | 0 |
| **Knowledge/competence** | | | | | | | | | | |  |
| Diabetes Knowledge Questionnaire (Spanish version) | 1 | very low | +/very low | 0 | ?/low | ^a^ | ?/very low | 0 | 0 | 0 | 0 |
| DMKT | 1 | low | +/high | 0 | +/high | ^a^ | 0 | 0 | 0 | +/high | 0 |
| PCSD-P | 1 | low | +/high | -/high | +/high | ^a^ | +/high | 0 | 0 | 0 | 0 |
| *Miller et al.* ^b^ | 1 | very low | +/very low | 0 | ?/low | 0 | ?/very low | 0 | 0 | +/low | 0 |
| DRNK | 1 | very low | +/low | 0 | -/low | 0 | +/low | 0 | 0 | +/low | 0 |
| KHLS-DM | 1 | low | +/moderate | +/high | +/high | ^a^ | ?/very low | 0 | 0 | 0 | 0 |
| **Treatment experience** | | | | | | | | | | |  |
| C-ITAS-HK | 1 | moderate | +/high | ?/moderate | -/high | ^a^ | ?/moderate | 0 | 0 | +/high | 0 |
| Ch-ASIQ | 1 | low | +/high | ?/moderate | -/high | ^a^ | 0 | 0 | 0 | 0 | 0 |
| **Treatment compliance** | | | | | | | | | | |  |
| Medical Prescription Knowledge questionnaire | 1 | low | +/high | 0 | ?/moderate | 0 | 0 | 0 | +/high | 0 | 0 |
| Attitude Scale | 1 | low | +/high | 0 | ?/moderate | 0 | 0 | 0 | +/high | 0 | 0 |
| MAT OADs | 1 | 0 | +/very low | 0 | +/low | 0 | 0 | 0 | +/low | 0 | 0 |
| MAT insulin | 1 | 0 | +/very low | 0 | -/low | 0 | 0 | 0 | +/low | 0 | 0 |
| **Symptoms** | | | | | | | | | | |  |
| FH-15 (Chinese version) | 1 | 0 | +/moderate | +/high | +/high | ^a^ | ?/moderate | 0 | 0 | 0 | 0 |
| FACIT-Fatigue Scale | 1 | low | +/moderate | ?/high | +/high | ^a^ | ?/very low | 0 | 0 | 0 | 0 |
| **Nutrition and physical activity** | | | | | | | | | | |  |
| Barriers to Fat Reduction Scale (Spanish version) | 1 | very low | +/very low | 0 | -/low | ^a^ | ?/very low | 0 | 0 | 0 | 0 |
| Barriers to Exercise Checklist (Spanish version) | 1 | very low | +/very low | 0 | ?/low | ^a^ | ?/very low | 0 | 0 | 0 | 0 |
| Food Habits Questionnaire (Spanish version) | 1 | very low | +/very low | 0 | ?/low | ^a^ | ?/very low | 0 | 0 | 0 | 0 |
| HAPA-based PA inventory | 1 | 0 | +/high | +/moderate | -/high | 0 | +/high | 0 | 0 | 0 | 0 |
| **Support** | | | | | | | | | | |  |
| HCCQ-P | 1 | low | +/high | -/high | +/high | ^a^ | +/high | 0 | 0 | 0 | 0 |
| The Diabetes Family Support and Conflict Scale (Turkish version) | 1 | low | +/moderate | ?/moderate | +/high | 0 | 0 | 0 | 0 | 0 | 0 |
| **Attitude/coping with diabetes** | | | | | | | | | | |  |
| Relationship Consciousness of Japanese Patients with Type 2 Diabetes Mellitus Scale | 1 | low | +/moderate | ?/moderate | +/high | 0 | 0 | 0 | 0 | +/high | 0 |
| DAAS | 1 | low | +/moderate | ?/moderate | +/high | 0 | 0 | 0 | 0 | 0 | 0 |
| **Obstacles and problem-solving** | | | | | | | | | | |  |
| DPMD | 1 | very low | +/very low | 0 | +/low | 0 | ?/very low | 0 | 0 | -/low | 0 |
| DOQ | 2 | low | +/moderate | 0 | -/high | 0 | 0 | 0 | +/high | +/high | 0 |

0: Measurement property was not assessed by the study/ not applicable to be assessed

^a^ Only translation was done. Cross-cultural validation was not the aim of the study

^b^PROMs without proper names are labelled based on the name of the original author who developed the instrument

+, positive; -, negative; ±, inconsistent; ?, indeterminate

**Abbreviations**: HSQ 2.0: Health Status Questionnaire 2.0; PACIC: Patient Assessment of Chronic Illness Care; PRO-DM-Thai: instrument for patient-reported outcomes in Thai patients with type 2 diabetes mellitus; DQOL: Diabetes Quality-of-Life Measure; AsianDQOL: Asian Diabetes Quality of Life; CUDOS-Chinese: Mandarin Chinese Version of the Clinically Useful Depression Outcome Scale; PHQ-9: Patient Health Questionnaire-9; DMSES: diabetes management self-efficacy scale; GR-DMSES: Greek version of DMSES; SDSCA: Summary of diabetes self-care activities measure; SDSCA-Ar: Arabic version of SDSCA; DSMI-20: Diabetes Self-Management Instrument Short Form; CIRS: Chronic Illness Resources Survey; DES-SF: Diabetes Empowerment Scale - Short Form; PDQ-11: Personal Diabetes Questionnaire; C-PDQ: Chinese version of PDQ; DMKT: Diabetes Mellitus Knowledge Test; PCSD-P: Persian Version of Perceived Competence Scale for Diabetes; DRNK: diabetes-related nutrition knowledge questionnaire; KHLS-DM: Korean Health Literacy Scale for Diabetes Mellitus; MAT OADs: Measurement of Adherence to Drug Therapy in Diabetes Mellitus – Oral Antidiabetics; MAT insulin: Measurement of Adherence to Drug Therapy in Diabetes Mellitus – Insulin Therapy; FH-15: Chinese version of the new fear of hypoglycemia scale; HAPA-based PA inventory: health action process approach (HAPA)-based PA inventory; HCCQ-P: Persian health care climate questionnaire; DAAS: Diabetes Adjustment Assessment Scale; DPMD: diabetes-specific measure of patient desire to participate in medical decision making; DOQ: Diabetes Obstacles Questionnaire

**References:**

1. Pibernik-Okanović M. Psychometric properties of the World Health Organisation quality of life questionnaire (WHOQOL-100) in diabetic patients in Croatia. Diabetes Res Clin Pract 2001;51:133-143
2. Kolawole BA, Mosaku SK, Ikem RT. A comparison of two measures of quality of life of Nigerian clinic patients with type 2 diabetes mellitus. Afr Health Sci 2009;9:161-166
3. Sreedevi A, Cherkil S, Kuttikattu DS, Kamalamma L, Oldenburg B. Validation of WHOQOL-BREF in Malayalam and Determinants of Quality of Life Among People With Type 2 Diabetes in Kerala, India. Asia Pac J Public Health 2016;28:62s-69s
4. Jahanlou AS, Alishan Karami N. WHO quality of Life-BREF 26 questionnaire: Reliability and validity of the Persian version and compare it with Iranian diabetics quality of life questionnaire in diabetic patients. Primary Care Diabetes 2011;5:103-107
5. Reba K, Birhane BW, Gutema H. Validity and Reliability of the Amharic Version of the World Health Organization's Quality of Life Questionnaire (WHOQOL-BREF) in Patients with Diagnosed Type 2 Diabetes in Felege Hiwot Referral Hospital, Ethiopia. J Diabetes Res 2019;2019:3513159
6. Maddigan SL, Feeny DH, Johnson JA. Construct validity of the RAND-12 and Health Utilities Index Mark 2 and 3 in type 2 diabetes. Qual Life Res 2004;13:435-448
7. Morgan BS, Buscemi CP, Fajardo VP. Assessing Instruments in a Cuban American Population With Type 2 Diabetes Mellitus. Journal of Transcultural Nursing 2004;15:139-146
8. Maddigan SL, Feeny DH, Majumdar SR, Farris KB, Johnson JA. Health Utilities Index mark 3 demonstrated construct validity in a population-based sample with type 2 diabetes. J Clin Epidemiol 2006;59:472-477
9. Matza LS, Boye KS, Yurgin N. Validation of two generic patient-reported outcome measures in patients with type 2 diabetes. Health Qual Life Outcomes 2007;5:47
10. Lee WJ, Song KH, Noh JH, Choi YJ, Jo MW. Health-related quality of life using the EuroQol 5D questionnaire in Korean patients with type 2 diabetes. J Korean Med Sci 2012;27:255-260
11. Sayah FA, Qiu W, Xie F, Johnson JA. Comparative performance of the EQ-5D-5L and SF-6D index scores in adults with type 2 diabetes. Qual Life Res 2017;26:2057-2066
12. McClure NS, Sayah FA, Ohinmaa A, Johnson JA. Minimally Important Difference of the EQ-5D-5L Index Score in Adults with Type 2 Diabetes. Value Health 2018;21:1090-1097
13. Wang P, Luo N, Tai ES, Thumboo J. The EQ-5D-5L is More Discriminative Than the EQ-5D-3L in Patients with Diabetes in Singapore. Value Health Reg Issues 2016;9:57-62
14. Konerding U, Elkhuizen SG, Faubel R, Forte P, Malmström T, Pavi E, Janssen MF. The validity of the EQ-5D-3L items: an investigation with type 2 diabetes patients from six European countries. Health Qual Life Outcomes 2014;12:181
15. Koh D, Abdullah AM, Wang P, Lin N, Luo N. Validation of Brunei's Malay EQ-5D Questionnaire in Patients with Type 2 Diabetes. PLoS One 2016;11:e0165555
16. Fan J, McCoy RG, Ziegenfuss JY, Smith SA, Borah BJ, Deming JR, Montori VM, Shah ND. Evaluating the structure of the Patient Assessment of Chronic Illness Care (PACIC) survey from the patient's perspective. Ann Behav Med 2015;49:104-111
17. Aung E, Ostini R, Dower J, Donald M, Coll JR, Williams GM, Doi SA. Patient Assessment of Chronic Illness Care (PACIC) in Type 2 Diabetes: A Longitudinal Study. Eval Health Prof 2016;39:185-203
18. Gugiu PC, Coryn C, Clark R, Kuehn A. Development and evaluation of the short version of the Patient Assessment of Chronic Illness Care instrument. Chronic Illn 2009;5:268-276
19. Gugiu C, Coryn CL, Applegate B. Structure and measurement properties of the Patient Assessment of Chronic Illness Care instrument. J Eval Clin Pract 2010;16:509-516
20. Abdul-Razak S, Ramli AS, Badlishah-Sham SF, Haniff J. Validity and reliability of the patient assessment on chronic illness care (PACIC) questionnaire: the Malay version. BMC Fam Pract 2018;19:119
21. Hu J, Gruber KJ, Hsueh KH. Psychometric properties of the Chinese version of the SF-36 in older adults with diabetes in Beijing, China. Diabetes Res Clin Pract 2010;88:273-281
22. Wan EYF, Choi EPH, Yu EYT, Chin WY, Fung CSC, Chan AKC, Lam CLK. Evaluation of the internal and external responsiveness of Short Form-12 Health Survey version 2 (SF-12v2) in patients with type 2 diabetes mellitus. Qual Life Res 2018;27:2459-2469
23. Talbot F, Nouwen A, Gingras J, Gosselin M, Audet J. The assessment of diabetes-related cognitive and social factors: the Multidimensional Diabetes Questionnaire. J Behav Med 1997;20:291-312
24. Pawar SS, Thakurdesai PA. Translation and validation of hindi version of the multidimensional questionnaire (MDQ) for quality of life assessment in type 2 diabetes mellitus patients in indian population. International Journal of Pharmaceutical Sciences and Research 2013;4:1827-1832
25. Gerber B, Smith EV, Jr., Girotti M, Pelaez L, Lawless K, Smolin L, Brodsky I, Eiser A. Using Rasch measurement to investigate the cross-form equivalence and clinical utility of Spanish and English versions of a diabetes questionnaire: a pilot study. J Appl Meas 2002;3:243-271
26. Akohoue SA, Wallston KA, Schlundt DG, Rothman RL. Psychometric evaluation of the short version of the Personal Diabetes Questionnaire to assess dietary behaviors and exercise in patients with type 2 diabetes. Eat Behav 2017;26:182-188
27. Cheng L, Leung DYP, Wu Y-N, Sit JWH, Yang M-Y, Li X-M. Psychometric properties of the modified Personal Diabetes Questionnaire among Chinese patients with type 2 diabetes. Evaluation & the Health Professions 2018;41:3-24
28. Chuayruang K, Sriratanaban J, Hiransuthikul N, Suwanwalaikorn S. Development of an instrument for patient-reported outcomes in Thai patients with type 2 diabetes mellitus (PRO-DM-Thai). Asian Biomedicine 2015;9:7-19
29. Cheng AY, Tsui EY, Hanley AJ, Zinman B. Developing a quality of life measure for Chinese patients with diabetes. Diabetes Res Clin Pract 1999;46:259-267
30. Jin X, Liu GG, Gerstein H, Levine M, Steeves K, Guan H, Li H, Xie F. Item reduction and validation of the Chinese version of Diabetes Quality-of-Life Measure (DQOL). Quality of Life Research 2017;26:124-125
31. Pakpour AH, Saffari M, Burri A. Translation and validation of an Iranian version of the Diabetes Quality of Life measure. Journal of Diabetes Investigation 2012;3:471-478
32. Yildirim A, Akinci F, Gozu H, Sargin H, Orbay E, Sargin M. Translation, cultural adaptation, cross-validation of the Turkish diabetes quality-of-life (DQOL) measure. Qual Life Res 2007;16:873-879
33. Bujang MA, Ismail M, Hatta N, Othman SH, Baharum N, Lazim SSM. Validation of the Malay version of Diabetes Quality of Life (DQOL) Questionnaire for Adult Population with Type 2 Diabetes Mellitus. Malays J Med Sci 2017;24:86-96
34. Bujang MA, Adnan TH, Mohd Hatta NKB, Ismail M, Lim CJ. A Revised Version of Diabetes Quality of Life Instrument Maintaining Domains for Satisfaction, Impact, and Worry. J Diabetes Res 2018;2018:5804687
35. Goh SG, Rusli BN, Khalid BA. Development and validation of the Asian Diabetes Quality of Life (AsianDQOL) Questionnaire. Diabetes Res Clin Pract 2015;108:489-498
36. Dudzińska M, Tarach JS, Burroughs TE, Zwolak A, Matuszek B, Smoleń A, Nowakowski A. Validation of the Polish version of Diabetes Quality of Life - Brief Clinical Inventory (DQL-BCI) among patients with type 2 diabetes. Archives of Medical Science 2014;10:891-898
37. Magwood GS, Jenkins C, Zapka J. Validation of diabetes health-related quality-of-life instruments using cognitive interviewing with older African Americans. J Nurs Meas 2009;17:195-220
38. Nagpal J, Kumar A, Kakar S, Bhartia A. The development of 'Quality of Life Instrument for Indian Diabetes patients (QOLID): a validation and reliability study in middle and higher income groups. J Assoc Physicians India 2010;58:295-304
39. Sato F, Mita T, Yamamoto R, Hirose T, Ito C, Tamura Y, Yokota A, Someya Y, Uchida T, Uchino H, Kawamori R, Gosho M, Ohmura C, Kanazawa A, Watada H. Reliability and validity of the Japanese version of the Diabetes Quality-Of-Life questionnaire for Japanese patients with type 2 diabetes mellitus. Diabetology International 2014;5:21-29
40. Oobe M, Tanaka M, Fuchigami M, Sakata T. Preparation of a quality of life (QOL) questionnaire for patients with type II diabetes and prospects for its clinical application. Fukuoka Igaku Zasshi 2007;98:379-387
41. Saffari M, Lin CY, O’Garo K, Koenig HG, Sanaeinasab H, Pakpour AH. Psychometric properties of Persian Diabetes-Mellitus Specific Quality of Life (DMQoL) questionnaire in a population-based sample of Iranians. International Journal of Diabetes in Developing Countries 2019;39:218-227
42. Hasan SS, Ahmadi K, Santigo R, Ahmed SI. The validity of the Menopause-specific Quality of Life questionnaire in women with type 2 diabetes. Climacteric 2014;17:456-464
43. Khader YS, Bataineh S, Batayha W. The Arabic version of Diabetes-39: Psychometric properties and validation. Chronic Illness 2008;4:257-263
44. de Queiroz FA, Pace AE, dos Santos CB. Cross-cultural adaptation and validation of the instrument Diabetes-39 (D-39): Brazilian version for type 2 diabetes mellitus patients-stage 1. Revista Latino-Americana de Enfermagem 2009;17:708-715
45. Jannoo Z, Yap BW, Musa KI, Lazim MA, Hassali MA. An audit of diabetes-dependent quality of life in patients with type 2 diabetes mellitus in Malaysia. Qual Life Res 2015;24:2297-2302
46. Fung CS, Wan EY, Yu CL, Wong CK. Validity and reliability of the 19-item Audit of Diabetes-Dependent Quality of Life (ADDQoL-19) questionnaire in Chinese patients with type 2 diabetes mellitus in primary care. Qual Life Res 2016;25:2373-2378
47. Abbatecola AM, Spazzafumo L, Fabbietti P, Testa R, Rabini RA, Bonfigli AR, Corsonello A, Lattanzio F, Paolisso G. Diabetes-related quality of life is enhanced by glycaemic improvement in older people. Diabet Med 2015;32:243-249
48. Soon SS, Goh SY, Bee YM, Poon JL, Li SC, Thumboo J, Wee HL. Audit of Diabetes-Dependent Quality of Life (ADDQoL) [Chinese Version for Singapore] questionnaire: reliability and validity among Singaporeans with type 2 diabetes mellitus. Appl Health Econ Health Policy 2010;8:239-249
49. Kong D, Ding Y, Zuo X, Su W, Xiu L, Lin M, Rao S, Yu S. Adaptation of the Audit of Diabetes-Dependent Quality of Life questionnaire to people with diabetes in China. Diabetes Res Clin Pract 2011;94:45-52
50. Lemon SC, Rosal MC, Welch G. Measuring quality of life in low-income, Spanish-speaking Puerto Ricans with type 2 diabetes residing in the mainland U.S. Qual Life Res 2011;20:1507-1511
51. Demirci H, Cinar Y, Bayram N, Bilgel N. Quality of life in type II diabetic patients in primary health care. Dan Med J 2012;59:A4468
52. Kamarul Imran M, Ismail AA, Naing L, Wan Mohamad WB. The reliability and validity of the Malay version of the 18-item audit of Diabetes Dependent Quality of Life (the Malay ADDQOL) questionnaire. Southeast Asian J Trop Med Public Health 2007;38:398-405
53. Elasy TA, Samuel-Hodge CD, DeVellis RF, Skelly AH, Ammerman AS, Keyserling TC. Development of a health status measure for older African-American women with type 2 diabetes. Diabetes Care 2000;23:325-329
54. Meadows KA, Abrams C, Sandbaek A. Adaptation of the Diabetes Health Profile (DHP-1) for use with patients with Type 2 diabetes mellitus: psychometric evaluation and cross-cultural comparison. Diabet Med 2000;17:572-580
55. Mulhern B, Labeit A, Rowen D, Knowles E, Meadows K, Elliott J, Brazier J. Developing preference-based measures for diabetes: DHP-3D and DHP-5D. Diabet Med 2017;34:1264-1275
56. Li J, Li Z, Zhao W, Pan H, Halloran EJ. The reliability and validity of the diabetes care profile for Chinese populations. Eval Health Prof 2015;38:200-218
57. Li T-C, Lin C-C, Liu C-S, Li C-I, Lee Y-D. Validation of the Chinese version of the diabetes impact measurement scales amongst people suffering from diabetes. Quality of Life Research: An International Journal of Quality of Life Aspects of Treatment, Care & Rehabilitation 2006;15:1613-1619
58. Kolawole BA, Abodunde O, Ikem RT, Fabiyi AK. A test of the reliability and validity of a diabetes specific quality of life scale in a Nigerian hospital. Qual Life Res 2004;13:1287-1295
59. Speight J, Khagram LA, Davies MJ. Generic and diabetes-specific well-being in the AT.LANTUS Follow-on study: further psychometric validation of the W-BQ28 indicates its utility in research and clinical practice in Type 2 diabetes in the UK. Diabet Med 2012;29:e345-353
60. Rankin SH, Galbraith ME, Johnson S. Reliability and validity data for a Chinese translation of the Center for Epidemiological Studies-Depression. Psychol Rep 1993;73:1291-1298
61. McHale M, Hendrikz J, Dann F, Kenardy J. Screening for depression in patients with diabetes mellitus. Psychosomatic Medicine 2008;70:869-874
62. Zhang Y, Ting RZ, Lam MH, Lam SP, Yeung RO, Nan H, Ozaki R, Luk AO, Kong AP, Wing YK, Sartorius N, Chan JC. Measuring depression with CES-D in Chinese patients with type 2 diabetes: the validity and its comparison to PHQ-9. BMC Psychiatry 2015;15:198
63. Carter J, Cogo-Moreira H, Herrmann N, Merino D, Yang P, Shah BR, Kiss A, Reitav J, Oh PI, Swardfager W. Validity of the Center for Epidemiological Studies Depression scale in Type 2 diabetes. Journal of Psychosomatic Research 2016;90:91-97
64. Kokoszka A. Depression in Diabetes Self-Rating Scale: A screening tool. Diabetologia Doswiadczalna i Kliniczna 2008;8:43-47
65. de Cock ESA, Emons WHM, Nefs G, Pop VJM, Pouwer F. Dimensionality and scale properties of the Edinburgh Depression Scale (EDS) in patients with type 2 diabetes mellitus: The DiaDDzoB study. BMC Psychiatry 2011;11
66. Zauszniewski JA, Chung C, Krafcik K, Sousa VD. Psychometric testing of the Depressive Cognition Scale in women with Type 2 diabetes. Journal of Nursing Measurement 2001;9:61-72
67. Hsu L-F, Kao C-C, Wang M-Y, Chang C-J, Tsai P-S. Psychometric testing of a Mandarin Chinese Version of the Clinically Useful Depression Outcome Scale for patients diagnosed with type 2 diabetes mellitus. International Journal of Nursing Studies 2014;51:1595-1604
68. Ting RZ, Nan H, Yu MW, Kong AP, Ma RC, Wong RY, Loo K, So WY, Chow CC, Ko GT, Wing YK, Chan JC. Diabetes-related distress and physical and psychological health in chinese type 2 diabetic patients. Diabetes Care 2011;34:1094-1096
69. Farm BAS, Perwitasari DA, Thobari JA, Cao Q, Krabbe PFM, Postma MJ. Translation, Revision, and Validation of the Diabetes Distress Scale for Indonesian Type 2 Diabetic Outpatients with Various Types of Complications. Value Health Reg Issues 2017;12:63-73
70. Hajos TR, Pouwer F, Skovlund SE, Den Oudsten BL, Geelhoed-Duijvestijn PH, Tack CJ, Snoek FJ. Psychometric and screening properties of the WHO-5 well-being index in adult outpatients with Type 1 or Type 2 diabetes mellitus. Diabet Med 2013;30:e63-69
71. Cichoń E, Kiejna A, Kokoszka A, Gondek T, Rajba B, Lloyd CE, Sartorius N. Validation of the Polish version of WHO-5 as a screening instrument for depression in adults with diabetes. Diabetes Res Clin Pract 2020;159:107970
72. Venkataraman K, Tan LS, Bautista DC, Griva K, Zuniga YL, Amir M, Lee YS, Lee J, Tai ES, Khoo EY, Wee HL. Psychometric Properties of the Problem Areas in Diabetes (PAID) Instrument in Singapore. PLoS One 2015;10:e0136759
73. Jannoo Z, Yap BW, Khan NM, Farcomeni A. Assessing Diabetes Distress Among Type 2 Diabetes Mellitus in Malaysia Using the Problem Areas in Diabetes Scale. Value Health Reg Issues 2019;18:159-164
74. Gross CC, Scain SF, Scheffel R, Gross JL, Hutz CS. Brazilian version of the Problem Areas in Diabetes Scale (B-PAID): validation and identification of individuals at high risk for emotional distress. Diabetes Res Clin Pract 2007;76:455-459
75. Eom YS, Park HS, Kim SH, Yang SM, Nam MS, Lee HW, Lee KY, Lee S, Kim YS, Park Ie B. Evaluation of stress in korean patients with diabetes mellitus using the problem areas in diabetes-Korea questionnaire. Diabetes Metab J 2011;35:182-187
76. Lee EH, Lee YW, Lee KW, Kim YS, Nam MS. Measurement of diabetes-related emotional distress using the Problem Areas in Diabetes scale: psychometric evaluations show that the short form is better than the full form. Health Qual Life Outcomes 2014;12:142
77. Huis In TVEMJ, Makine C, Nouwen A, Karşda C, Kadioǧlu P, Karşda K, Pouwer F. Validation of the Turkish version of the problem areas in diabetes scale. Cardiovascular Psychiatry and Neurology 2011;
78. Papathanasiou A, Koutsovasilis A, Shea S, Philalithis A, Papavasiliou S, Melidonis A, Lionis C. The Problem Areas in Diabetes (PAID) scale: psychometric evaluation survey in a Greek sample with type 2 diabetes. J Psychiatr Ment Health Nurs 2014;21:345-353
79. Siaw M, Tai B, Lee J. Psychometric properties of the Chinese version of problem areas in diabetes scale (SG-PAID-c) among high-risk polypharmacy patients with uncontrolled type 2 diabetes in Singapore. Value in Health 2016;19:A901
80. Welch G, Schwartz CE, Santiago-Kelly P, Garb J, Shayne R, Bode R. Disease-related emotional distress of Hispanic and non-Hispanic type 2 diabetes patients. Ethn Dis 2007;17:541-547
81. Arzaghi SM, Mahjouri MY, Heshmat R, Khashayar P, Larijani B. Psychometric properties of the Iranian version of the Problem Areas in Diabetes scale (IR-PAID-20). Iranian Journal of Diabetes and Lipid Disorders 2011;10:1-7
82. Zhang Y, Ting R, Lam M, Lam J, Nan H, Yeung R, Yang W, Ji L, Weng J, Wing Y-K, Sartorius N, Chan JCN. Measuring depressive symptoms using the Patient Health Questionnaire-9 in Hong Kong Chinese subjects with type 2 diabetes. Journal of Affective Disorders 2013;151:660-666
83. Janssen EP, Köhler S, Stehouwer CD, Schaper NC, Dagnelie PC, Sep SJ, Henry RM, van der Kallen CJ, Verhey FR, Schram MT. The Patient Health Questionnaire-9 as a Screening Tool for Depression in Individuals with Type 2 Diabetes Mellitus: The Maastricht Study. J Am Geriatr Soc 2016;64:e201-e206
84. Udedi M, Muula AS, Stewart RC, Pence BW. The validity of the patient health Questionnaire-9 to screen for depression in patients with type-2 diabetes mellitus in non-communicable diseases clinics in Malawi. BMC Psychiatry 2019;19:81
85. Lupascu N, Timar B, Albai A, Roman D, Potre O, Timar R. Validation and cross-cultural adaptation of the depression patient’s health questionnaire - 9 in the Romanian population of patients with type 2 diabetes mellitus. Diabetes, Metabolic Syndrome and Obesity: Targets and Therapy 2019;12:841-849
86. Bijl JV, Poelgeest-Eeltink AV, Shortridge-Baggett L. The psychometric properties of the diabetes management self-efficacy scale for patients with type 2 diabetes mellitus. J Adv Nurs 1999;30:352-359
87. Sturt J, Hearnshaw H, Wakelin M. Validity and reliability of the DMSES UK: A measure of self-efficacy for type 2 diabetes self-management. Primary Health Care Research and Development 2010;11:374-381
88. Lee EH, Van Der Bijl J, Shortridge-Baggett LM, Han SJ, Moon SH. Psychometric properties of the diabetes management self-efficacy scale in Korean patients with type 2 diabetes. International Journal of Endocrinology 2015;2015
89. Fappa E, Efthymiou V, Landis G, Rentoumis A, Doupis J. Validation of the Greek Version of the Diabetes Management Self-Efficacy Scale (GR-DMSES). Adv Ther 2016;33:82-95
90. Pace AE, Gomes LC, Bertolin DC, Loureiro H, Bijl JV, Shortridge-Baggett LM. Adaptation and validation of the Diabetes Management Self-Efficacy Scale to Brazilian Portuguese. Rev Lat Am Enfermagem 2017;25:e2861
91. Messina R, Rucci P, Sturt J, Mancini T, Fantini MP. Assessing self-efficacy in type 2 diabetes management: validation of the Italian version of the Diabetes Management Self-Efficacy Scale (IT-DMSES). Health Qual Life Outcomes 2018;16:71
92. Thojampa S. Psychometric evaluation of the Thai translation of the Self-Efficacy for Diabetes Scale in delaying the progress of diabetic nephropathy in adults with T2DM. International Journal of Africa Nursing Sciences 2018;9:1-3
93. Zheng J, Wang Y, Ye X, Xiao L, Ye J, Li X, Zhong M. Validation of diabetes medication self-efficacy scale in Chinese with type 2 diabetes. Patient Preference and Adherence 2018;12:2517-2525
94. Sousa VD, Hartman SW, Miller EH, Carroll MA. New measures of diabetes self-care agency, diabetes self-efficacy, and diabetes self-management for insulin-treated individuals with type 2 diabetes. Journal of Clinical Nursing 2009;18:1305-1312
95. Toobert DJ, Hampson SE, Glasgow RE. The summary of diabetes self-care activities measure: results from 7 studies and a revised scale. Diabetes Care 2000;23:943-950
96. Kav S, Akman A, Dogan N, Tarakci Z, Bulut Y, Hanoglu Z. Turkish validity and reliability of the summary of diabetes self-care activities measure for patients with type 2 diabetes mellitus. Journal of Clinical Nursing 2010;19:2933-2935
97. Kamradt M, Bozorgmehr K, Krisam J, Freund T, Kiel M, Qreini M, Flum E, Berger S, Besier W, Szecsenyi J, Ose D. Assessing self-management in patients with diabetes mellitus type 2 in Germany: validation of a German version of the Summary of Diabetes Self-Care Activities measure (SDSCA-G). Health Qual Life Outcomes 2014;12:185
98. Adarmouch L, Sebbani M, Elyacoubi A, Amine M. Psychometric Properties of a Moroccan Version of the Summary of Diabetes Self-Care Activities Measure. J Diabetes Res 2016;2016:5479216
99. Sukkarieh-Haraty O, Howard E. Psychometric Properties of the Arabic Version of the Summary of Diabetes Self-Care Activities Instrument. Res Theory Nurs Pract 2016;30:60-69
100. Choi EJ, Nam M, Kim SH, Park CG, Toobert DJ, Yoo JS, Chu SH. Psychometric properties of a Korean version of the summary of diabetes self-care activities measure. Int J Nurs Stud 2011;48:333-337
101. Mendonça SCB, Zanetti ML, Sawada NO, Barreto IDC, Andrade JS, Otero LM. Construction and validation of the Self-care Assessment Instrument for patients with type 2 diabetes mellitus. Rev Lat Am Enfermagem 2017;25:e2890
102. Khagram L, Martin CR, Davies MJ, Speight J. Psychometric validation of the Self-Care Inventory-Revised (SCI-R) in UK adults with type 2 diabetes using data from the AT.LANTUS Follow-on study. Health Qual Life Outcomes 2013;11:24
103. García AA. The Diabetes Symptom Self-Care Inventory: development and psychometric testing with Mexican Americans. J Pain Symptom Manage 2011;41:715-727
104. McCaskill GM, Bolland KA, Burgio KL, Leeper J. Development and validation of a diabetes self-management instrument for older African-Americans. Soc Work Health Care 2016;55:381-394
105. Chang SJ, Song M, Im E-O. Psychometric evaluation of the Korean version of the Diabetes Self-efficacy Scale among South Korean older adults with type 2 diabetes. Journal of Clinical Nursing 2014;23:2121-2130
106. van der Heijden MM, Pouwer F, Pop VJ. Psychometric properties of the Exercise Self-efficacy Scale in Dutch Primary care patients with type 2 diabetes mellitus. Int J Behav Med 2014;21:394-401
107. Fain JA. Psychometric properties of the Spanish Version of the Diabetes Self-management Assessment Report Tool. Diabetes Educ 2007;33:827-832
108. Nakawatase Y, Taru C, Tsutou A, Shiotani H, Kido Y, Ohara T, Ogawa W, Miyawaki I. Development of an evaluation scale for self-management behavior related to physical activity of type 2 diabetic patients. Diabetes Care 2007;30:2843-2848
109. Thojampa S, Mawn B. Psychometric evaluation of the Thai translation of the Diabetes Self-management Questionnaire in type 2 diabetes. International Journal of Nursing Sciences 2017;4:236-238
110. Bukhsh A, Lee SWH, Pusparajah P, Schmitt A, Khan TM. Psychometric properties of the Diabetes Self-Management Questionnaire (DSMQ) in Urdu. Health Qual Life Outcomes 2017;15:200
111. Dao-Tran T-H, Anderson DJ, Chang AM, Seib C, Hurst C. Vietnamese version of diabetes self-management instrument: Development and psychometric testing. Research in Nursing & Health 2017;40:177-184
112. Lee CL, Lin CC, Anderson R. Psychometric evaluation of the Diabetes Self-Management Instrument Short Form (DSMI-20). Appl Nurs Res 2016;29:83-88
113. Seo K, Song M, Choi S, Kim SA, Chang SJ. Development of a scale to measure diabetes self-management behaviors among older Koreans with type 2 diabetes, based on the seven domains identified by the American Association of Diabetes Educators. Jpn J Nurs Sci 2017;14:161-170
114. Peyrot M, Bushnell DM, Best JH, Martin ML, Cameron A, Patrick DL. Development and validation of the self-management profile for type 2 diabetes (SMP-T2D). Health Qual Life Outcomes 2012;10:125
115. Mahjouri MY, Arzaghi SM, Heshmat R, Khashayar P, Esfahani EN, Larijani B. Psychometric properties of the Iranian version of Diabetes Empowerment Scale (IR-DES-28). Journal of Diabetes and Metabolic Disorders 2012;11:1-5
116. Hara Y, Iwashita S, Okada A, Tajiri Y, Nakayama H, Kato T, Nakao M, Tsuboi K, Breugelmans R, Ishihara Y. Development of a novel, short, self-completed questionnaire on empowerment for patients with type 2 diabetes mellitus and an analysis of factors affecting patient empowerment. Biopsychosoc Med 2014;8:19
117. Ching SM, Yee A, Lee PY, Ramachandran V, Shum KM, Ismael NF, Wan Sulaiman WA, Hoo FK, Foo YL, Lee KW, Danaee M, Tan KA. Psychometric properties of the Malay version of the diabetes empowerment scale among hospital Serdang type 2 diabetes mellitus patients using exploratory factor analysis. Health Qual Life Outcomes 2020;18:23
118. Chaves FF, Reis IA, Pagano AS, Torres HC. Translation, cross-cultural adaptation and validation of the Diabetes Empowerment Scale - Short Form. Rev Saude Publica 2017;51:16
119. Sousa MR, Almeida M, Loureiro H, Martins T. Study of the Psychometric Properties of the Diabetes Empowerment Scale Short Form (DES-SF). Portuguese Journal of Public Health 2019;
120. Saffari M, Karimi T, Koenig HG, Al-Zaben F. Psychometric evaluation of the Persian version of the Type 2 Diabetes and Health Promotion Scale (T2DHPS): a diabetes-specific measure of lifestyle. Scand J Caring Sci 2015;29:603-612
121. Yildiz E, Kavuran E. The validity and reliability of the Type 2 Diabetes and Health Promotion Scale Turkish version: A methodological study. Scandinavian Journal of Caring Sciences 2018;32:417-421
122. Wang RH, Lin LY, Cheng CP, Hsu MT, Kao CC. The psychometric testing of the diabetes health promotion self-care scale. J Nurs Res 2012;20:122-130
123. Lewis KS, Jennings AM, Ward JD, Bradley C. Health belief scales developed specifically for people with tablet-treated type 2 diabetes. Diabet Med 1990;7:148-155
124. Brown SA, Becker HA, Garcia AA, Barton SA, Hanis CL. Measuring health beliefs in Spanish-speaking Mexican Americans with type 2 diabetes: Adapting an existing instrument. Research in Nursing & Health 2002;25:145-158
125. Kartal A, Ozsoy SA. Validity and reliability study of the Turkish version of Health Belief Model Scale in diabetic patients. International Journal of Nursing Studies 2007;44:1447-1458
126. Lujan J. Testing the diabetes knowledge and health beliefs of Mexican Americans on the Texas-Mexico border. Hispanic Health Care International 2008;6:9-20
127. Laranjo L, Dias V, Nunes C, Paiva D, Mahoney B. Translation and Validation of the Patient Activation Measure in Portuguese People with Type 2 Diabetes Mellitus. Acta Med Port 2018;31:382-390
128. Chernyak N, Jülich F, Kasperidus J, Stephan A, Begun A, Kaltheuner M, Icks A. Time cost of diabetes: Development of a questionnaire to assess time spent on diabetes self-care. J Diabetes Complications 2017;31:260-266
129. Padhy M, Padiri RA, Hariharan M, Rana S. Diabetes Mellitus Knowledge Test: development, psychometric evaluation, and establishing norms for Indian population. International Journal of Diabetes in Developing Countries 2019;39:206-217
130. Matin H, Nadrian H, Sarbakhsh P, Shaghaghi A. Factorial Structure and Psychometric Analysis of the Persian Version of Perceived Competence Scale for Diabetes (PCSD-P). Behav Sci (Basel) 2019;9
131. Miller CK, Achterberg CL. Reliability and validity of a nutrition and food-label knowledge test for women with Type 2 diabetes mellitus. Journal of Nutrition Education 2000;32:43-48
132. Miller C, Edwards L. Development and validation of a shelf inventory to evaluate household food purchases among older adults with diabetes mellitus. J Nutr Educ Behav 2002;34:261-267
133. Samuel-Hodge CD, DeVellis RF, Ammerman A, Keyserling TC, Elasy TA. Reliability and validity of a measure of perceived diabetes and dietary competence in African American women with type 2 diabetes. Diabetes Educ 2002;28:979-988
134. Han CY, Zheng X, Lee LF, Chan CGB, Lee YQ, Zailani NA, Ng K, Bhaskaran K. Development of a diabetes-related nutrition knowledge questionnaire for individuals with type 2 diabetes mellitus in Singapore. Nutr Diet 2019;76:567-573
135. Finbråten HS, Guttersrud Ø, Nordström G, Pettersen KS, Trollvik A, Wilde-Larsson B. Validating the Functional, Communicative, and Critical Health Literacy Scale Using Rasch Modeling and Confirmatory Factor Analysis. J Nurs Meas 2018;26:341-363
136. Kang SJ, Sim KH, Song BR, Park J-E, Chang SJ, Park C, Lee MS. Validation of the health literacy scale for diabetes as a criterion-referenced test with standard setting procedures. Patient Education and Counseling 2018;101:1468-1476
137. Lee EH, Lee YW. First-order vs. second-order structural validity of the Health Literacy Scale in patients with diabetes. Scand J Caring Sci 2018;32:441-447
138. Luo H, Patil SP, Wu Q, Bell RA, Cummings DM, Adams AD, Hambidge B, Craven K, Gao F. Validation of a combined health literacy and numeracy instrument for patients with type 2 diabetes. Patient Educ Couns 2018;101:1846-1851
139. Ashok Kumar M, Shanmugasundaram P. Development of a validated questionnaire to assess knowledge and awareness among uncontrolled diabetic patients toward diabetes mellitus. Drug Invention Today 2019;11:801-806
140. Ashok Kumar M, Shanmugasundaram P. A validation study of questionnaire towards mobile based health applications in uncontrolled diabetic population of India (South). Diabetes Metab Syndr 2019;13:2106-2110
141. Finbråten HS, Pettersen KS, Wilde-Larsson B, Nordström G, Trollvik A, Guttersrud Ø. Validating the European Health Literacy Survey Questionnaire in people with type 2 diabetes: Latent trait analyses applying multidimensional Rasch modelling and confirmatory factor analysis. J Adv Nurs 2017;73:2730-2744
142. Kontodimopoulos N, Arvanitaki E, Aletras VH, Niakas D. Psychometric properties of the Greek Diabetes Treatment Satisfaction Questionnaire. Health Qual Life Outcomes 2012;10:17
143. Brod M, Christensen T, Kongsø JH, Bushnell DM. Examining and interpreting responsiveness of the Diabetes Medication Satisfaction measure. J Med Econ 2009;12:309-316
144. Azami G, Lam SK, Shariff-Ghazali S, Said SM, Aazami S, Mozafari M, Taghinejad H. Validation of the Iranian/Persian Version of the Perceived Therapeutic Efficacy Scale for Type 2 Diabetes. Arch Iran Med 2018;21:356-361
145. Demirtaş A, Akbayrak N. Development of an assessment scale for treatment compliance in type 2 Diabetes Mellitus in Turkish population: Psychometric evaluation. International Journal of Nursing Sciences 2017;4:244-251
146. Çinar D, Yava A. Validity and reliability of functional assessment of chronic illness treatment-fatigue scale in Turkish patients with type 2 diabetes. Endocrinol Diabetes Nutr 2018;65:409-417
147. Ishii H, Shin H, Tosaki T, Haga T, Nakajima Y, Shiraiwa T, Watanabe N, Koizumi M, Nakajima H, Okada S, Mashitani T, Mohri T, Akai Y. Reproducibility and Validity of a Questionnaire Measuring Treatment Burden on Patients with Type 2 Diabetes: Diabetic Treatment Burden Questionnaire (DTBQ). Diabetes Therapy 2018;9:1001-1019
148. Moock JH, F.; Ziegeler, D.; Kubiak, T.; Kohlmann, T. Development and testing of the insulin treatment experience questionnaire (ITEQ). Patient 2010;3:45-58
149. Rubin RR, Peyrot M. Psychometric properties of an instrument for assessing the experience of patients treated with inhaled insulin: the inhaled insulin treatment questionnaire (IITQ). Health Qual Life Outcomes 2010;8:32
150. Snoek FJ, Skovlund SE, Pouwer F. Development and validation of the insulin treatment appraisal scale (ITAS) in patients with type 2 diabetes. Health Qual Life Outcomes 2007;5:69
151. Lee KP. Translation and validation of the Insulin Treatment Appraisal Scale in Hong Kong primary care patients. J Diabetes Investig 2018;9:311-320
152. Saritas SC, Erci B, Sahin I, Timocin E. Validity study of the Turkish version of the barriers to insulin treatment questionnaire. International Journal of Diabetes in Developing Countries 2019;39:247-253
153. Fu SN, Chin WY, Wong CK, Yeung VT, Yiu MP, Tsui HY, Chan KH. Development and validation of the Chinese Attitudes to Starting Insulin Questionnaire (Ch-ASIQ) for primary care patients with type 2 diabetes. PLoS One 2013;8:e78933
154. Osborn CY, Gonzalez JS. Measuring insulin adherence among adults with type 2 diabetes. J Behav Med 2016;39:633-641
155. Dos Santos RBP, Trevisan DD, Do Nascimento RA, São-João TM, Lima MHM, Rodrigues RCM. Psychometric performance of the Brazilian version the "Insulin Management Diabetes Self-Efficacy Scale" for patient with Type 2 diabetes mellitus. Medicina (Brazil) 2018;51:121-130
156. Anderson RT, Skovlund SE, Marrero D, Levine DW, Meadows K, Brod M, Balkrishnan R. Development and Validation of the Insulin Treatment Satisfaction Questionnaire. Clinical Therapeutics: The International Peer-Reviewed Journal of Drug Therapy 2004;26:565-578
157. Sakthong P, Chabunthom R, Charoenvisuthiwongs R. Psychometric properties of the Thai version of the 8-item Morisky Medication Adherence Scale in patients with type 2 diabetes. Ann Pharmacother 2009;43:950-957
158. Wang Y, Lee J, Toh MP, Tang WE, Ko Y. Validity and reliability of a self-reported measure of medication adherence in patients with Type 2 diabetes mellitus in Singapore. Diabet Med 2012;29:e338-344
159. Lee WY, Ahn J, Kim JH, Hong YP, Hong SK, Kim YT, Lee SH, Morisky DE. Reliability and validity of a self-reported measure of medication adherence in patients with type 2 diabetes mellitus in Korea. J Int Med Res 2013;41:1098-1110
160. Wang J, Bian RW, Mo YZ. Validation of the Chinese version of the eight-item Morisky medication adherence scale in patients with type 2 diabetes mellitus. Journal of Clinical Gerontology and Geriatrics 2013;4:119-122
161. Zongo A, Guénette L, Moisan J, Grégoire JP. Predictive Validity of Self-Reported Measures of Adherence to Noninsulin Antidiabetes Medication against Control of Glycated Hemoglobin Levels. Can J Diabetes 2016;40:58-65
162. Zongo A, Guénette L, Moisan J, Guillaumie L, Lauzier S, Grégoire JP. Revisiting the internal consistency and factorial validity of the 8-item Morisky Medication Adherence Scale. SAGE Open Medicine 2016;4
163. Kristina SA, Putri LR, Riani DA, Ikawati Z, Endarti D. Validity of self-reported measure of medication adherence among diabetic patients in Indonesia. International Research Journal of Pharmacy 2019;10:144-148
164. Ishii H, Oda E. Reproducibility and validity of a satisfaction questionnaire on hypoglycemic agents: The Oral Hypoglycemic Agent Questionnaire (OHA-Q). Diabetology International 2012;3:152-163
165. Peyrot M, Harshaw Q, Shillington AC, Xu Y, Rubin RR. Validation of a tool to assess medication treatment satisfaction in patients with Type 2 diabetes: the Diabetes Medication System Rating Questionnaire (DMSRQ). Diabet Med 2012;29:1060-1066
166. Prado-Aguilar CA, Martínez YV, Segovia-Bernal Y, Reyes-Martínez R, Arias-Ulloa R. Performance of two questionnaires to measure treatment adherence in patients with Type-2 Diabetes. BMC Public Health 2009;9
167. Fall E, Gauchet A, Izaute M, Horne R, Chakroun N. Validation of the French version of the Beliefs about Medicines Questionnaire (BMQ) among diabetes and HIV patients. European Review of Applied Psychology / Revue Europeenne de Psychologie Appliquee 2014;64:335-343
168. Chung WW, Chua SS, Mei Lai PS, Morisky DE. The Malaysian Medication Adherence Scale (MALMAS): Concurrent validity using a clinical measure among people with type 2 diabetes in Malaysia. PLoS ONE 2015;10
169. Gomes-Villas Boas LC, de Lima MLSAP, Pace AE. Adherence to treatment for diabetes mellitus: Validation of instruments for oral antidiabetics and insulin. Revista Latino-Americana de Enfermagem 2014;22:11-18
170. Kim C-J, Park E, Schlenk EA, Kim M, Kim DJ. Psychometric evaluation of a Korean version of the Adherence to Refills and Medications Scale (ARMS) in adults with type 2 diabetes. The Diabetes Educator 2016;42:188-198
171. Peyrot M, Xu Y, Rubin RR. Development and validation of the Diabetes Medication System Rating Questionnaire-Short Form. Diabet Med 2014;31:1237-1244
172. Kawata AK, Wilson H, Ong SH, Kulich K, Coyne K. Development and Psychometric Evaluation of the Hypoglycemia Perspectives Questionnaire in Patients with Type 2 Diabetes Mellitus. Patient 2016;9:395-407
173. De Mesa UR, Anonuevo-Cruz MC, Nicodemus N, Reyes NG. Development and validation of a questionnaire evaluating impaired hypoglycemia awareness among adult filipino patients with type 2 diabetes mellitus. Journal of the ASEAN Federation of Endocrine Societies 2017;32:158-164
174. Liu YQ, Xiong SQ, Sang M, Li YF, Anarte Ortiz MT, Xing QL, Xu HM, Jin CD. Reliability and validity of the Chinese version of the new fear of hypoglycemia scale: FH-15. International Journal of Nursing Sciences 2018;5:343-351
175. Lee EH, Lee KW, Song R, Snoek FJ, Moon SH. Psychometric evaluation of the Korean version of the Diabetes Symptom Checklist-Revised (DSC-R) for patients with type 2 diabetes. Health Qual Life Outcomes 2014;12:77
176. Arbuckle RA, Humphrey L, Vardeva K, Arondekar B, Danten-Viala M, Scott JA, Snoek FJ. Psychometric evaluation of the Diabetes Symptom Checklist-Revised (DSC-R)--a measure of symptom distress. Value Health 2009;12:1168-1175
177. Naegeli AN, Stump TE, Hayes RP. A psychometric evaluation of the diabetes symptom checklist-revised (DSC-R) cognitive distress, fatigue, hyperglycemia, and hypoglycemia subscales in patients with type 1 and type 2 diabetes. Diabetes, Metabolic Syndrome and Obesity: Targets and Therapy 2010;3:27-30
178. Sato E, Suzukamo Y, Miyashita M, Kazuma K. Development of a diabetes diet-related quality-of-life scale. Diabetes Care 2004;27:1271-1275
179. Sato E, Ochiai R, Shibayama T, Nishigaki M, Abe Y, Sawa T, Suzukamo Y, Kazuma K. Reliability and validity of revised and short form versions of diabetes diet-related quality of life scale. Diabetology International 2017;8:181-192
180. Hayes RP, DeLozier AM. Reliability, validity, and responsiveness of the Impact of Weight on Self-Perceptions Questionnaire (IW-SP) in individuals with type 2 diabetes and obesity. Diabetes Technol Ther 2015;17:210-214
181. Payo RM, Mendez XG, Cano CP, Alvarez JS. Development and validation of a questionnaire for assessing the characteristics of diet and physical activity in patients with type 2 diabetes. Psicothema 2018;30:116-122
182. Rohani H, Eslami AA, Ghaderi A, Jafari-Koshki T, Sadeghi E, Bidkhori M, Raei M. Validation and psychometric evaluation of physical activity belief scale among patients with type 2 diabetes mellitus: an application of health action process approach. Health Promot Perspect 2016;6:71-79
183. Teng Y, Wang S, Wang N, Muhuyati. STOP-Bang questionnaire screening for obstructive sleep apnea among Chinese patients with type 2 diabetes mellitus. Archives of Medical Science 2018;14:971-978
184. Donovan LM, Yu L, Bertisch SM, Buysse DJ, Rueschman M, Patel SR. Responsiveness of Patient-Reported Outcomes to Treatment Among Patients With Type 2 Diabetes Mellitus and OSA. Chest 2020;157:665-672
185. Shakibazadeh E, Rashidian A, Larijani B, Shojaeezadeh D. Psychometric properties of the Iranian version of Resources and Support for Chronic illness Self-management scale in patients with type 2 diabetes. International Journal of Preventive Medicine 2012;3:84-90
186. Hara Y, Iwashita S, Ishii K, Inada C, Okada A, Tajiri Y, Nakayama H, Kato T, Nishida K, Ogata Y, Omori H, Morinaga T, Yamaguchi M, Nakao M, Tsuboi K, Breugelmans R, Ishihara Y. The reliability and validity of the Japanese version of the Diabetes Family Behavior Checklist (DFBC) for assessing the relationship between type 2 diabetes mellitus patients and their families with respect to adherence to treatment regimen. Diabetes Res Clin Pract 2013;99:39-47
187. Littlewood K, Cummings DM, Lutes L, Solar C. Psychometric Properties of the Family Support Scale Adapted for African American Women with Type 2 Diabetes Mellitus. Ethn Dis 2015;25:193-199
188. Matin H, Nadrian H, Jahangiry L, Sarbakhsh P, Shaghaghi A. Psychometric properties of the Persian health care climate questionnaire (HCCQ-P): Assessment of type 2 diabetes care supportiveness in Iran. Patient Preference and Adherence 2019;13:783-793
189. Sofulu F, Unsalavdal Rn E, Arkan Rn B. Validity and reliability of the diabetes family support and conflict scale in Turkish. Acta Medica Mediterranea 2017;33:107-114
190. Kato A, Takada M, Hashimoto H. Reliability and validity of the Japanese version of the self-stigma scale in patients with type 2 diabetes. Health Qual Life Outcomes 2014;12:179
191. Browne JL, Ventura AD, Mosely K, Speight J. Measuring the Stigma Surrounding Type 2 Diabetes: Development and Validation of the Type 2 Diabetes Stigma Assessment Scale (DSAS-2). Diabetes Care 2016;39:2141-2148
192. Koike M, Inagaki M, Tasaki K, Matsui K, Horiguchi T, Oda A, Susanto T. Validation of the relationship consciousness of Japanese Patients with type 2 diabetes scale. International Journal of Nursing Sciences 2019;6:31-37
193. Hara Y, Koyama S, Morinaga T, Ito H, Kohno S, Hirai H, Kikuchi T, Tsuda T, Ichino I, Takei S, Yamada K, Tsuboi K, Breugelmans R, Ishihara Y. The reliability and validity of the Japanese version of the Appraisal of Diabetes Scale for type 2 diabetes patients. Diabetes Res Clin Pract 2011;91:40-46
194. Lee EH, Lee YW, Lee KW, Nam M, Kim YS, Han SJ. A Korean version of the Appraisal of Diabetes Scale (ADS-K): psychometric evaluation with a population of Koreans with type 2 diabetes. J Transcult Nurs 2015;26:270-278
195. Ebrahimi H, Karimi Moonaghi H, Asghari Jafarabadi M, Namdar Areshtanab H, Jouybari L. Development and Preliminary Validation of Diabetes Adjustment Assessment Scale (DAAS): a New Measure of Adjustment with Type 2 Diabetes. J Caring Sci 2016;5:145-152
196. Mahjouri MY, Arzaghi SM, Qorbani M, Esfahani EN, Larijani B. Evaluation of psychometric properties of the third version of the Iranian Diabetes Attitude Scale (IR-DAS-3). Iranian Journal of Diabetes and Lipid Disorders 2011;10:1-6
197. Persson L-O, Erichsen M, Wandell P, Gafvels C. Psychometric evaluation of a coping questionnaire in two independent samples of people with diabetes. Stress and Health: Journal of the International Society for the Investigation of Stress 2013;29:286-296
198. Traina SB, Mathias SD, Colwell HH, Crosby RD, Abraham C. The diabetes intention, attitude, and behavior questionnaire: Evaluation of a brief questionnaire to measure physical activity, dietary control, maintenance of a healthy weight, and psychological antecedents. Patient Preference and Adherence 2016;10:213-222
199. Martinez NC, Sousa VD. Cross-cultural validation and psychometric evaluation of the Spanish Brief Religious Coping Scale (S-BRCS). Journal of Transcultural Nursing 2011;22:248-256
200. Golin CE, DiMatteo MR, Leake B, Duan N, Gelberg L. A diabetes-specific measure of patient desire to participate in medical decision making. Diabetes Educ 2001;27:875-886
201. Hearnshaw H, Wright K, Dale J, Sturt J, Vermeire E, van Royen P. Development and validation of the Diabetes Obstacles Questionnaire (DOQ) to assess obstacles in living with Type 2 diabetes. Diabet Med 2007;24:878-882
202. Vandekerckhove M, Vermeire E, Weeren A, Van Royen P. Validation of the Diabetes Obstacles Questionnaire (DOQ) to assess obstacles in living with type 2 diabetes in a Belgian population. Prim Care Diabetes 2009;3:43-47
203. Pilv L, Vermeire E, Rätsep A, Moreau A, Nikolić D, Petek D, Yaman H, Oona M, Kalda R. Development and validation of the short version of the diabetes obstacles questionnaire (DOQ-30) in six European countries. Eur J Gen Pract 2016;22:16-22
204. Hill-Briggs F, Yeh H-C, Gary TL, Batts-Turner M, D'Zurilla T, Brancati FL. Diabetes Problem-Solving Scale development in an adult, African American sample. The Diabetes Educator 2007;33:291-299
205. Manit A, Tuicomepee A, Jiamjarasrangsi W, Taneepanichskul S. Development of needs and resources for self-management assessment instrument in Thais with type 2 diabetes: cross-cultural adaptation. J Med Assoc Thai 2011;94:1304-1313
206. Abubakari A-R, Jones MC, Lauder W, Kirk A, Devendra D, Anderson J. Psychometric properties of the Revised Illness Perception Questionnaire: Factor structure and reliability among African-origin populations with type 2 diabetes. International Journal of Nursing Studies 2012;49:672-681
207. Traina SB, Colwell HH, Crosby RD, Mathias SD. Pragmatic measurement of health satisfaction in people with type 2 diabetes mellitus using the Current Health Satisfaction Questionnaire. Patient Relat Outcome Meas 2015;6:103-115
208. Chew BH, Vos RC, Heijmans M, Shariff-Ghazali S, Fernandez A, Rutten G. Validity and reliability of a Malay version of the brief illness perception questionnaire for patients with type 2 diabetes mellitus. BMC Med Res Methodol 2017;17:118
209. Egede LE, Ellis C. Development and psychometric properties of the 12-item diabetes fatalism scale. J Gen Intern Med 2010;25:61-66
